# Longitudinal single-cell and TCR repertoire profiling characterizes clonal entrapment in patients with pMMR/MSS locally advanced rectal cancer

**DOI:** 10.1038/s41421-026-00900-w

**Published:** 2026-06-30

**Authors:** Zhichun Tang, Mingxuan Zhu, Shidong Zhao, Dazhi Pang, Yancheng Cui, Guole Lin, Aiwen Wu, Yilin Lin, Xinyang Jiang, Wei Zhang, Nan Xiong, Changjiang Yang, Caihong Wang, Shan Wang, Yingjiang Ye, Fan Bai, Zhanlong Shen

**Affiliations:** 1 Biomedical Pioneering Innovation Center (BIOPIC) and School of Life Sciences, Peking-Tsinghua Center for Life Sciences (CLS), State Key Laboratory of Metabolic Dysregulation & Prevention and Treatment of Esophageal Cancer, Peking University, Beijing, China; 2 Department of Gastroenterological Surgery, Peking University People’s Hospital, Beijing, China; 3 Laboratory of Surgical Oncology, Beijing Key Laboratory of Precision Diagnosis, Treatment and Translational Research for Colorectal Cancer, Peking University People’s Hospital, Beijing, China; 4 School of Basic Medical Sciences, Tsinghua University, Beijing, China; 5 Department of General Surgery, Peking Union Medical College Hospital, Chinese Academy of Medical Sciences & Peking Union Medical College, Beijing, China; 6 State Key Laboratory of Holistic Integrative Management of Gastrointestinal Cancers, Beijing Key Laboratory of Carcinogenesis and Translational Research, Unit III, Gastrointestinal Cancer Center, Peking University Cancer Hospital & Institute, Beijing, China; 7 Department of Thoracic Surgery, the First Affiliated Hospital of Fujian Medical University, Fuzhou, Fujian China; 8 Vanke School of Public Health, Tsinghua University, Beijing, China; 9 Peking University Beijing-Tianjin-Hebei Biomedical Pioneering Innovation Center, Tianjin, China; 10 Beijing Advanced Center of Cellular Homeostasis and Aging-Related Diseases, Institute of Advanced Clinical Medicine, Peking University, Beijing, China

**Keywords:** Colorectal cancer, Cancer therapeutic resistance

## Abstract

Elevated intratumoral immune inflammation prior to treatment is typically associated with better outcomes in hot tumors treated with immune checkpoint inhibitors (ICIs). However, we observed a paradox in pMMR/MSS locally advanced rectal cancer (LARC) patients, where a subset with elevated baseline immune inflammation exhibited worse outcomes after combined radiotherapy and ICI treatment compared with patients with minimal immune inflammation. To investigate this counterintuitive finding, we performed paired scRNA-seq and scTCR-seq on longitudinally collected samples, including tumor biopsies (pre-treatment, post-radiotherapy, and post-immunotherapy) and peripheral blood mononuclear cells (pre-treatment and post-immunotherapy), from 20 pMMR/MSS LARC patients treated with sequential radiotherapy and ICI therapy (NCT06493240). We propose the concept of clonal entrapment to explain this phenomenon. Specifically, our profiling results reveal that increased *HLA-DQA2* expression in dendritic cells and upregulated *GDF15* expression in treatment-resistant tumor cells correlate with the restricted expansion of novel tumor-reactive TCR clonotypes. Consequently, the immune response is limited primarily by pre-existing TCR clonotypes within the tumor, especially those partially expanded under chronic inflammation, leading to the expansion of TCR clonotypes derived mainly from pre-treatment CD8^+^ T cell pools following ICI therapy. By identifying this feature of the pMMR/MSS LARC microenvironment, our study provides a high-resolution framework for understanding resistance to sequential radiotherapy and ICI therapy.

## Introduction

Colorectal cancer (CRC) is the third most commonly diagnosed malignancy and the second leading cause of cancer-related mortality worldwide^1,2^. In China, 69.6% of rectal cancer cases are diagnosed as locally advanced rectal cancer (LARC), defined as T3–T4 or N-positive stages^3,4^. Although immune checkpoint inhibitors (ICIs), such as anti-PD-1 therapy, have been widely reported to induce high clinical complete response rates in LARC patients with mismatch repair-deficient or microsatellite instability-high (dMMR/MSI-H) tumors^5–9^, more than 85% of patients with proficient mismatch repair or microsatellite stable (pMMR/MSS) LARC tumors do not benefit from immunotherapy alone^10–14^. This lack of response is attributed primarily to a lower tumor neoantigen load and limited immune infiltration^15–17^. As a result, recent clinical strategies have focused on using radiotherapy to convert these “cold” pMMR/MSS tumors into immune-inflamed “hot” tumors, followed by subsequent immunotherapy, which has demonstrated promising therapeutic efficacy^18–21^. However, clinical studies have reported that even after combined radiotherapy and immunotherapy, some pMMR/MSS tumors still develop resistance^22,23^.

There has been extensive research on the dynamic changes in tumor-specific CD8^+^ T cells in hot tumors before and after ICI therapy Two main theories have emerged: Clonal replacement^24^ and clonal revival^25^. The clonal replacement theory suggests that, following ICI treatment, the tumor microenvironment (TME) of basal and squamous cell carcinomas contains primarily newly recruited TCR clones from the periphery, which are absent in the TME prior to treatment. This implies a limited ability of pre-existing T cells to be revived within the tumor. On the other hand, the clonal revival theory posits that after ICI therapy, T cell clones already present in the non-small cell lung cancer TME are reactivated, expanding alongside newly recruited clones from the periphery to exert anti-tumor effects.

Subsequent studies have further explored these theories in other hot tumors, such as melanoma^26,27^, head and neck squamous cell carcinoma^28,29^, renal cell carcinoma^30^, and even MSI-H CRC^31^, attempting to identify which mechanism correlates most significantly with improved ICI treatment outcomes. However, a gap remains in understanding how the dynamic changes in tumor-specific CD8^+^ T cells in cold tumors after ICI therapy are related to prognosis, largely because of the ineffectiveness of ICI treatments alone in cold tumors. With the recent increase in combining radiotherapy with ICI therapy, there is an urgent need to update these theories to account for the enhanced immunogenicity and responses observed in such cases.

In this study, we collected paired tumor tissue and peripheral blood samples from 20 pMMR/MSS LARC patients who underwent combined radiotherapy and ICI therapy. Tumor tissue samples were obtained before treatment, after radiotherapy, and after ICI therapy, along with peripheral blood samples taken before and after treatment. On the basis of the scRNA-seq and scTCR-seq results from these samples, we propose the concept of clonal entrapment to explain a paradoxical phenomenon observed in both LARC bulk RNA-seq and scRNA-seq cohorts: Some patients with pre-existing localized chronic immune inflammation before treatment exhibit poorer outcomes with combined radiotherapy and immunotherapy than those with lower baseline levels of inflammation. Clonal entrapment refers to a phenomenon in which the TCR clones expanded by subsequent ICI therapy are observed to be derived predominantly from the pre-existing CD8^+^ T cell pool — particularly those that had already undergone partial expansion under the chronic state of inflammation, which may contribute to the suboptimal efficacy of ICI treatment.

## Results

### Establishment and characterization of the treatment cohort

**Fig. 1:**
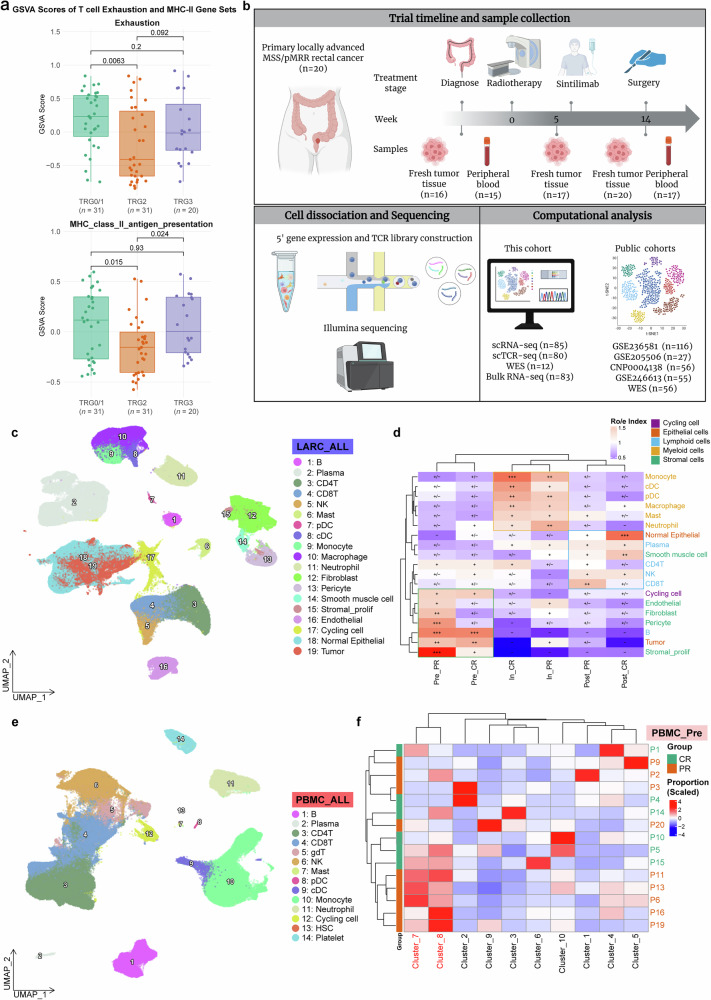
Single-cell atlas of tumor and peripheral cellular dynamics during the treatment of patients with LARC. **a** Boxplots displaying GSVA scores for the T-cell exhaustion gene set (comprising *PDCD1*, *HAVCR2*, *CTLA4*, *TIGIT*, and *CXCL13*) and the MHC-II antigen presentation gene set (GO:0019886) across different TRG groups in the CRC bulk RNA-seq cohort (*n* = 82 after quality control) at baseline. *P*-values were calculated using the Wilcoxon rank-sum test. **b** Schematic diagram showing the study design, treatment timeline, sample collection points, and data types analyzed. **c** UMAP plot depicting the distribution of major cell types across 238,680 cells from all LARC tumor samples. **d** Heatmap showing the distributions of major cell types between the CR and PR groups at baseline (Pre), post-radiotherapy (In), and post-immunotherapy (Post). The *Ro/e* index (see Methods for calculation) is represented using the symbols +++ (> 2), ++ (> 1.5), + (> 1), and +/- ( > 0.5). **e** UMAP plot showing the distribution of major cell types across 243,609 PBMCs from all peripheral blood samples. **f** Heatmap showing the enrichment of each co-occurrence cluster in the pre-treatment samples.

Between 2018 and 2019, we collected primary tumor tissue samples from 83 patients with LARC prior to treatment and performed bulk RNA-seq. All patients received radiotherapy-based regimens, and their tumor regression grades (TRGs) were documented. The clinical characteristics of these patients are summarized in Supplementary Table S1. Among the 49 patients with known microsatellite status, only one had dMMR/MSI-H, while the remainder had pMMR/MSS. Compared with patients with an intermediate treatment response (TRG 2), those with the poorest response (TRG 3) and those with the best response (TRG 0/1) displayed similarly elevated baseline T cell exhaustion scores and MHC class II antigen presentation scores (Fig. 1a), although deconvolution analysis did not indicate exclusive T cell enrichment in either the TRG 0/1 or TRG 3 samples (Supplementary Fig. S1d). Given that the tumor cells in the TRG 0/1 group were largely eradicated by radiotherapy, we focused on understanding why patients in the TRG 3 group had the poorest therapeutic response, which was worse than that in the TRG 2 group wherein minimal to no immune inflammation was detected, despite exhibiting localized immune inflammation prior to treatment. Similarly, bulk RNA-seq was performed on tumor tissue samples from an independent cohort of 114 treatment-naïve patients with LARC^32^, and among the three pMMR/MSS subtypes classified on the basis of immune inflammation, the IG2 subtype — with intermediate immune inflammation — exhibited a worse trend in prognosis than the IG1 subtype, although the degree of immune inflammation was lowest in patients with the IG1 subtype (Supplementary Fig. S1a, b). At baseline, increased T cell exhaustion and MHC class II antigen presentation are associated with improved responses to ICI therapies^33–36^. This raises the question of whether patients in the TRG 3 group may benefit from immunotherapy. However, since ICI-based immunotherapy is not currently a standard first-line treatment for patients with MSS CRC, the aim of our study was to explore the potential benefits of combining radiotherapy with immunotherapy to improve treatment efficacy and to identify potential predictive biomarkers for this therapeutic strategy.

Herein, we monitored the complete treatment course of 22 patients diagnosed with LARC who received care at Peking University People’s Hospital between January 2023 and August 2024. Among these patients, 20 underwent long-term preoperative radiotherapy combined with anti-PD-1 immunotherapy, while the remaining two received chemotherapy alone without immunotherapy. All tumors were confirmed to be mismatch repair-proficient and microsatellite-stable (pMMR/MSS) on the basis of immunohistochemical (IHC) staining for mismatch repair (MMR) proteins. All patients exhibited tumor regression following treatment. Patients with a tumor regression grade (TRG) of 0 were classified into the complete response (CR) group (*n* = 7), while the remaining patients were assigned to the partial response (PR) group (*n* = 15). No treatment-related or immune-related adverse events or perioperative or 30-day postoperative surgical complications were observed. Sphincter-preserving surgery was achieved in 18/20 patients (90%). Detailed clinical information for all patients is summarized in Supplementary Table S2.

Of the 20 patients who received the combined radiotherapy and anti-PD-1 immunotherapy regimen, we obtained consent from 18 patients and collected a range of samples. These included tumor tissue and peripheral blood samples collected before treatment (LARC_Pre and PBMC_Pre, respectively), tumor tissue samples after radiotherapy (LARC_In), and tumor tissue and peripheral blood samples following immunotherapy (LARC_Post and PBMC_Post, respectively). In total, we collected 85 paired fresh samples, which were subsequently subjected to scRNA-seq and scTCR-seq. Our primary analysis focuses on these samples to address two key questions: (1) What pre-treatment factors contributed to the differing clinical responses between patients who achieved a CR and those who achieved a PR, and did these differences diminish or disappear as treatment progressed? (2) Why did patients in the PR group retain residual tumor cells after radiotherapy and immunotherapy, and what are the mechanisms underlying their resistance?

To assess whether our findings in patients with pMMR/MSS CRC extended to immune-responsive tumors, we analyzed two independent cohorts of patients with dMMR/MSI-H CRC who received anti-PD-1 monotherapy. The first cohort consisted of 22 patients for whom processed scRNA-seq data were available in GSE236581^31^. The second cohort consisted of 19 patients for whom processed scRNA-seq data were available in GSE205506^37^. Moreover, we performed whole-exome sequencing (WES) on tumor tissue samples from three patients (P8, P9, and P10) at three time points: Before treatment (Pre), after radiotherapy (In), and after immunotherapy (Post). For each of these samples, the WES results from the corresponding PBMC_Post samples were used as controls. We also analyzed a published WES dataset comprising 56 paired LARC tumor tissue samples collected before and after chemoradiotherapy for validation (Fig. 1b).

As mentioned earlier, for the two patients in this study who received chemotherapy alone, we collected tumor and peripheral blood samples pre- and post-chemotherapy for scRNA-seq. Through further integration with scRNA-seq data from 29 rectal cancer patients who received neoadjuvant chemotherapy^38^, we explored the following question: (3) Were there differences in tumor microenvironment remodeling after radiotherapy and chemotherapy administration, and which approach was more suitable for combination with subsequent immunotherapy?

### Single-cell atlas of tumor and peripheral cellular dynamics during treatment of patients with LARC

We first constructed a single-cell atlas of tumor and peripheral cellular dynamics during treatment of patients with LARC. After obtaining the scRNA-seq data for the 85 samples mentioned above, we performed rigorous quality control (see Methods for details) and excluded 5 sequencing samples with low quality. After removing low-quality cells and doublets, we retained a total of 482,289 cells, comprising 238,680 from LARC tissue and 243,609 PBMCs. The cells from LARC tissue were categorized into four major groups: lymphocytes (B cells, plasma cells, CD4^+^ T cells, CD8^+^ T cells, γδ T cells and NK cells), myeloid cells (mast cells, plasmacytoid dendritic cells (pDC), conventional dendritic cells (cDC), monocytes, macrophages, and neutrophils), stromal cells (fibroblasts, smooth muscle cells, and endothelial cells), and epithelial cells (normal epithelial and tumor cells), along with a group of proliferating cells (cycling cells) (Fig. 1c; Supplementary Fig. S1c). The effects of different treatment regimens on TME remodeling in LARC tissue were sufficiently pronounced to overshadow the differences in the TME between patients who achieved a CR and patients who achieved a PR at various stages of treatment. Specifically, patients in the CR and PR groups exhibited predominant enrichment of stromal and tumor cells in the TME prior to treatment. Radiotherapy reduced the abundances of stromal and tumor cells and increased the proportion of innate immune cells, particularly myeloid cells, within the TME. This finding is consistent with previous studies showing that radiotherapy can activate the innate immune system by inducing immunogenic cell death (ICD) and releasing tumor-associated antigens^39,40^. Following immunotherapy, the TME in the CR and PR groups shifted toward a state with greater immune infiltration. There were notable increases in the abundances of T lymphocytes and plasma cells, indicating a transition from a “cold” tumor to a “hot” tumor, a hallmark of immune activation and increased adaptive immune responses (Fig. 1d).

Given that changes in TME cell composition after radiotherapy and immunotherapy were similar between the CR and PR groups, we sought to determine whether pre-existing differences could explain the variation in treatment response. Our analysis revealed that, prior to treatment, there was no significant difference in the proportion of immune cells in LARC tissue between the two groups, except for a slightly greater proportion of neutrophils in the CR group (*P* = 0.092) (Supplementary Figs. S1e, S2a). This finding suggests that differences in treatment response may have arisen from differences in molecular expression that affect the function of similar cell populations in the two groups. Furthermore, we categorized all the PBMCs into 14 major cell types and 59 subtypes (Fig. 1e; Supplementary Fig. S2b). On the basis of the correlations among cell proportions before treatment, we grouped these 59 subtypes into 10 co-occurrence clusters (Supplementary Fig. S2c). Cluster 7 and Cluster 8 were enriched in the PR group before treatment and were composed primarily of activated lymphocytes, including CD4T_MHCII and tTreg_MHCII cell subtypes expressing high levels of MHC-II molecules^41^, the CD8T_*HAVCR2* cell subtype expressing the exhaustion marker *HAVCR2*, and plasma cells (Fig. 1f). These findings suggest that pre-existing differences existed between the PR and CR groups prior to treatment, with patients in the PR group potentially exhibiting a state of sustained immune activation, which may be associated with systemic chronic inflammation.

### Lower *HLA-DQA2* expression in dendritic cells at baseline is associated with better treatment responses in patients with LARC

**Fig. 2:**
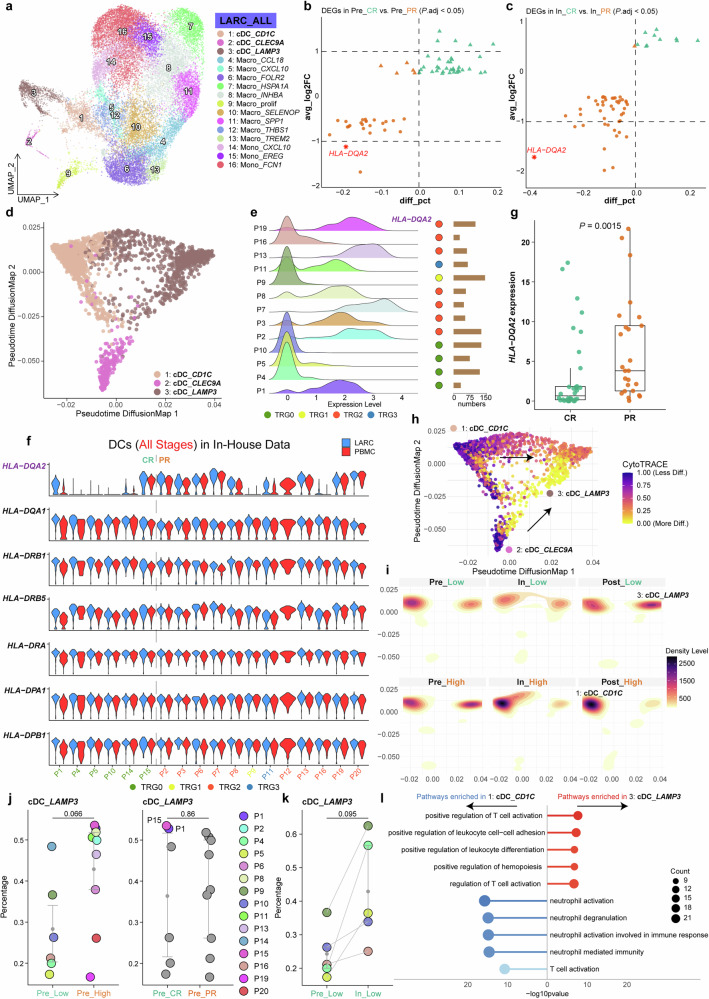
Lower baseline *HLA-DQA2* expression in dendritic cells is associated with better treatment responses in patients with LARC. **a** UMAP projection of macrophages, monocytes, and cDCs from all LARC samples. Each dot represents a single cell and is colored according to its cell type. **b**, **c** Volcano plots showing significantly upregulated or downregulated genes (*P* < 0.05) in the CR group compared with the PR group before treatment (**b**) and after radiotherapy (**c**), along with corresponding avg_log_2_FC and diff_pct values (diff_pct: the difference in the proportion of cells expressing each gene between the two groups). **d** Diffusion map illustrating the developmental trajectory of DCs, with the colors of each DC subset consistent with those in **a**. **e** Ridge plot showing *HLA-DQA2* expression in cDCs across post-radiotherapy LARC samples, with sample groupings and total cDC counts indicated on the right. **f** Violin plot showing the expression of MHC-II molecules, including *HLA-DQA2*, in cDCs from LARC tissue (blue) and peripheral blood (red) at all treatment stages for each patient in this dataset. CR patients are shown on the left, and PR patients are shown on the right. **g** Boxplot showing *HLA-DQA2* expression in DCs from individual patients in the CR and PR groups; *P* values were calculated using the Wilcoxon rank-sum test. **h** Diffusion map illustrating the CytoTRACE scores of individual DCs, with higher scores indicating later positions along the developmental trajectory and arrows denoting the developmental direction. **i** Density plot showing the distribution of DC subtypes in the low- and high-*HLA-DQA2* groups across treatment stages, with coordinates matched to those in **d**, **h**. **j** Boxplots comparing the proportions of the cDC_*LAMP3* subtype between the low- and high-*HLA-DQA2* groups (left) and between the CR and PR groups (right) at baseline, with each dot representing one patient; *P* values were calculated using the Wilcoxon rank-sum test. **k** Boxplot showing changes in cDC_*LAMP3* subtype proportions before and after radiotherapy in low-*HLA-DQA2* patients, with patient colors matched to those in **j**; *P* values were calculated using the Wilcoxon signed-rank test. **l** Enrichment analysis showing the top five upregulated pathways in the cDC_*LAMP3* (red) and cDC_*CD1C* (blue) subtypes.

Since myeloid cells, such as macrophages, monocytes, and DCs, are among the first innate immune populations to accumulate in the TME after radiotherapy, we extracted these cells from patients at all LARC stages and subdivided them into 16 subtypes to study their functions (Fig. 2a; Supplementary Fig. S3a). Similarly, monocytes and DCs from PBMCs were subdivided into 11 subtypes for detailed analysis (Supplementary Fig. S3c). However, the proportions of these subtypes were largely consistent between the CR and PR groups before treatment (*P* > 0.05), suggesting that no single subtype predominantly contributed to the effects of combined radiotherapy and immunotherapy. We therefore compared the differences in gene expression between these subtypes in the CR and PR groups at each treatment stage. In addition to identifying differentially expressed genes (DEGs) with *P*_adj_ < 0.05, we incorporated diff_pct (the difference in the proportion of cells expressing a gene between the CR and PR groups) to better evaluate DEGs and mitigate the influence of individual patient variation on avg_log_2_FC. Compared with those in the CR group, the avg_log_2_FC and diff_pct of *HLA-DQA2* expression were greater before treatment and after radiotherapy in the PR group (Fig. 2b, c).

Unlike *HLA-DQA1*, which is polymorphic, *HLA-DQA2* is considered a non-polymorphic gene and is expressed primarily in antigen-presenting cells such as DCs^42^. Therefore, we extracted all DCs (Fig. 2d) from LARC tissues of all patients following radiotherapy and measured *HLA-DQA2* expression to exclude the possibility that differences in *HLA-DQA2* expression between the CR and PR groups were driven by a single patient. The results revealed that patients P4, P5, P10, and P14 (with a TRG of 0) and patient P9 (with a TRG of 1) exhibited low *HLA-DQA2* expression. This pattern was not attributable to a small number of captured DCs, as these patients contributed a greater number of DCs (Fig. 2e). We also observed that patients P1 and P15, both of which had high *HLA-DQA2* expression, achieved CR. These findings are consistent with our previous observations from the bulk RNA-seq cohort, where a group of patients with higher pre-treatment immune activity achieved a TRG of 0 (Fig. 1a; Supplementary Fig. S1b). We were then concerned with why a greater number of patients with high *HLA-DQA2* expression, compared with those with low expression, do not derive maximal benefit from combined radiotherapy and immunotherapy. Considering that treatment-induced inflammation can sometimes increase the expression of MHC-II molecules, we next assessed the expression of *HLA-DQA2* and other MHC-II molecules in DCs from LARC tissues and PBMCs at all treatment stages for each patient. LARC tissues from patients with low *HLA-DQA2* expression consistently exhibited this pattern before treatment, after radiotherapy, and after immunotherapy, as did their PBMCs before and after treatment (Fig. 2f). This suggested that low *HLA-DQA2* expression was unlikely the result of sequencing dropout in a single sample, as the likelihood of dropout co-occurring across all five samples from the same patient is extremely low. Instead, this likely reflected an intrinsic patient characteristic that may have contributed to a greater likelihood of achieving a clinical CR.

Given the limited sample size of our cohort, we validated our findings using data from two additional Chinese cohorts of dMMR/MSI-H CRC patients who received anti-PD-1 monotherapy. scRNA-seq data from CRC tissue and peripheral blood samples collected before, during, and after treatment were reported for the cohort in the study by Zhang et al.^31^. In contrast, Deng et al. provided scRNA-seq data from CRC tissue samples collected before and after treatment^37^. Patients with low *HLA-DQA2* expression were more likely to experience favorable outcomes (*P* = 0.0015) (Fig. 2g; Supplementary Fig. S3d, e). Moreover, data from the Zhang et al. dataset confirmed that low *HLA-DQA2* expression in DCs from these patients was not attributable to low cell counts and did not vary by tissue type (CRC vs. peripheral blood) or sampling time point (Supplementary Fig. S3f). This consistency across cohorts and sampling conditions reinforces the reliability of our findings and suggests that low *HLA-DQA2* expression may serve as a robust biomarker for predicting favorable responses to immunotherapy.

Although the function of *HLA-DQA2* in DCs is not fully understood, recent studies have highlighted its potential role in predicting the outcomes of individuals after SARS-CoV-2 infection. Specifically, increased *HLA-DQA2* expression is associated with milder symptoms or even asymptomatic cases after SARS-CoV-2 infection^43,44^. We performed pseudotemporal trajectory analysis on all the DCs (Fig. 2d) and found that cDC_*LAMP3*, a mature DC subtype, was located at the end of the developmental trajectory (Fig. 2h). This subset was also enriched in pre-treatment samples from patients with high *HLA-DQA2* expression (*P* = 0.066) (Fig. 2i, j), including those in the CR group (P1 and P15). This explains why there was no significant difference in the proportion of the cDC_*LAMP3* subset between the CR and PR groups at baseline. In contrast, a greater proportion of patients with low *HLA-DQA2* expression had a higher proportion of the immature DC subtype cDC_*CD1C* before treatment (*P* = 0.05) (Supplementary Fig. S3b). However, these cells rapidly matured and became activated after radiotherapy and then enriched after ICI therapy, with functional characteristics such as increased positive regulation of T cell activation, positive regulation of leukocyte cell–cell adhesion, and differentiation. In contrast, the DCs from patients with high *HLA-DQA2* expression formed a TME dominated by the cDC_*CD1C* subtype after combined radiotherapy and immunotherapy and played a greater role in neutrophil activation (Fig. 2i, k, l). This dynamic change in DC behavior may be because although higher *HLA-DQA2* expression increases immune responses to viral infections, it may contribute to chronic or dysregulated immune activation in the context of cancer, making it difficult to benefit from immunotherapy and leading to distinct outcomes in these two types of patients.

These findings may also help explain our earlier observation that patients in the PR group paradoxically exhibited stronger baseline immune inflammation in their peripheral blood, which could be driven by an increased antigen presentation capacity associated with high *HLA-DQA2* expression (Fig. 1f), and this activated MHC-II antigen presentation pathway may reflect a strong positive correlation with Treg activity among all CD4^+^ T cells (Supplementary Fig. S3i). Integration of multiple CRC scRNA-seq cohorts revealed that *HLA-DQA2* is more highly expressed in DCs within the TME of patients with metastatic CRC, while it is expressed at lower levels in the peripheral blood of healthy individuals (Supplementary Fig. S3g). In contrast, *HLA-DQA1* and *HLA-DRB1* are highly expressed in the DCs of normal adjacent cancer tissues and are consistently highly expressed in nearly all patients (Supplementary Fig. S3h). These findings address the first question: Low baseline *HLA-DQA2* expression is associated with better treatment response, and this hallmark does not disappear as treatment progresses.

### Persistent expansion of pre-existing tumor-infiltrating CD8^+^ T cell clones leads to a suboptimal treatment response

**Fig. 3:**
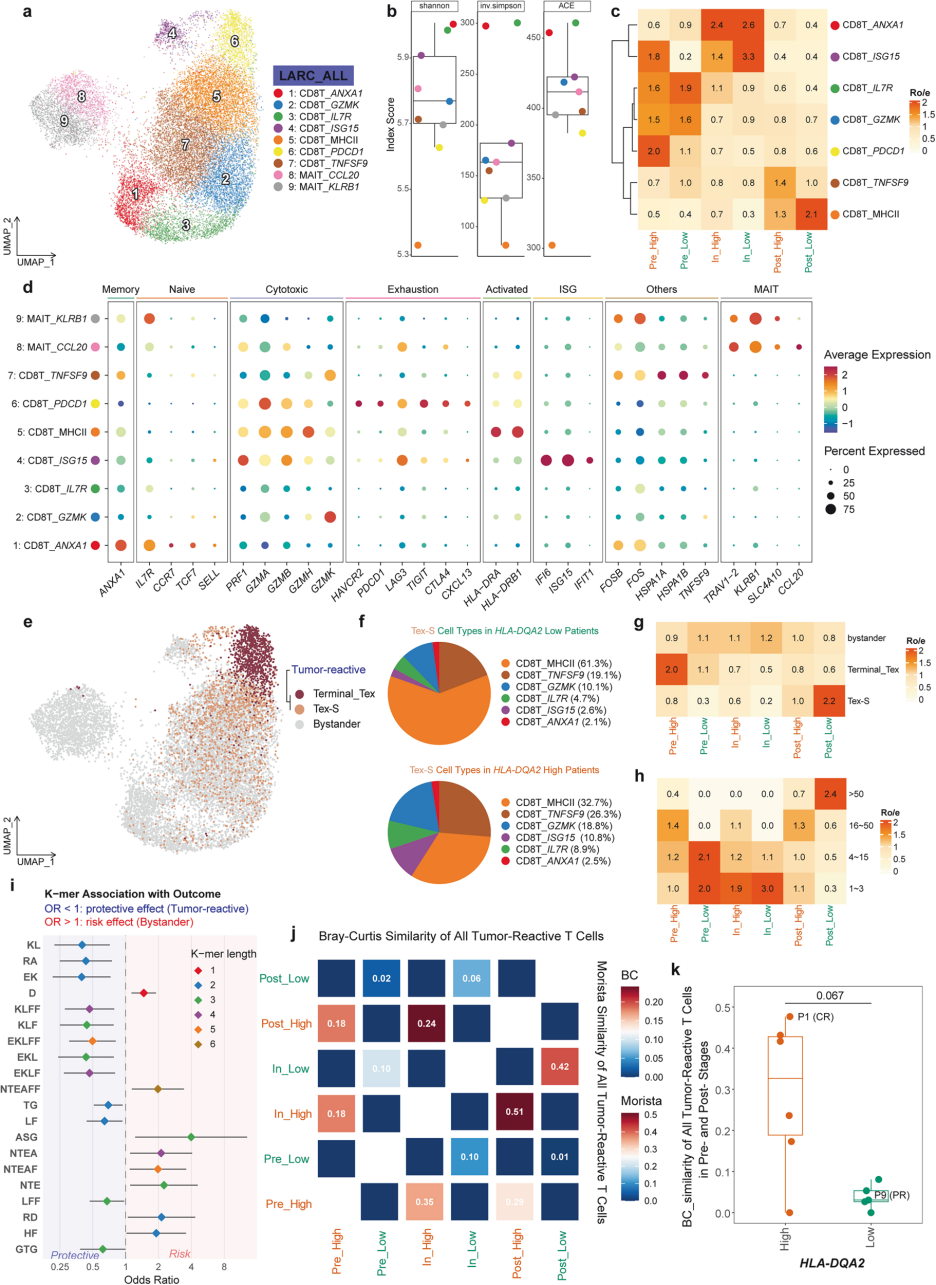
Persistent expansion of pre-existing tumor-infiltrating CD8^+^ T cell clones drives a suboptimal treatment response. **a** UMAP projection of CD8^+^ T cells from all LARC samples. Each dot represents a single cell and is colored according to its cell type. **b** Box plots showing TCR clonotype diversity among CD8^+^ T cell subtypes. The colors correspond to the subsets shown in **a**. **c** Heatmap showing the distributions of CD8^+^ T cells in the low- and high-*HLA-DQA2* groups at baseline (Pre), post-radiotherapy (In), and post-immunotherapy (Post), with higher *Ro/e* index values (see Methods) indicating stronger enrichment tendencies. **d** Bubble plot showing the expression of marker genes in CD8^+^ T cell subtypes. **e** UMAP projection of terminally exhausted CD8^+^ T (Tex) cells, Tex-shared CD8^+^ T cells (Tex-S cells; sharing TCR clonotypes with Tex cells), and bystander CD8^+^ T cells. **f** Pie charts showing the distribution of CD8^+^ T cell subtypes among Tex-S cells in the low-*HLA-DQA2* (top) and high-*HLA-DQA2* (bottom) groups. **g** Heatmap showing the distributions of Tex, Tex-S, and bystander CD8^+^ T cells in the low- and high-*HLA-DQA2* groups at baseline (Pre), post-radiotherapy (In), and post-immunotherapy (Post). **h** Heatmap showing the distributions of TCR clonotypes of different sizes across the six groups, with “>50” indicating clonotypes shared by at least 50 CD8^+^ T cells. **i** Lollipop plot showing k-mer enrichment in tumor-reactive CD8^+^ T cells (protective effect) and bystander CD8^+^ T cells (risk effect). **j** Heatmap showing the Bray–Curtis (BC) similarity index (upper left) and Morisita index (lower right) of tumor-reactive CD8^+^ T cells in LARC tissues across the Pre, In, and Post groups with low and high *HLA-DQA2* expression. **k** Boxplot showing paired differences in the Bray–Curtis similarity index of tumor-reactive CD8^+^ T cells before and after treatment in patients with low and high *HLA-DQA2* expression; *P* values were calculated using the Wilcoxon rank-sum test.

We next systematically delineated the dynamic clonal evolution of CD8^+^ T cells during sequential radiotherapy and immunotherapy in patients with low *HLA-DQA2* expression (CR group) versus those with high expression (PR group). CD8^+^ T cells from patients with all LARC stages were classified into nine subtypes. Among them, the CD8T_*PDCD1* subtype highly expressed exhaustion markers such as *HAVCR2*, *PDCD1*, *LAG3*, *TIGIT*, *CTLA4*, and *CXCL13* and was therefore defined as a terminally exhausted CD8^+^ T cell (Tex) subtype (Fig. 3a, d). In contrast, the CD8T_MHCII subtype exhibited high expression of activation markers, including MHC-II molecules and various granzymes, and was thus defined as an activated CD8^+^ T cell subtype. We reconstructed TCR sequences from all the samples using TCR sequencing data and found that the clonal diversity and richness of the naïve subtypes, CD8T_*ANXA1* and CD8T_*IL7R*, were greatest (Fig. 3b). In contrast, activated CD8^+^ T cells (CD8T_MHCII) displayed the lowest diversity, which was even lower than that of terminally exhausted CD8^+^ T cells (CD8T_*PDCD1*). This may be attributed to CD8T_MHCII being the most significantly expanded subtype in both groups following immunotherapy, representing a highly activated population associated with the immune response in this treatment context. Furthermore, we observed that the Tex subtype was enriched primarily in baseline samples from the high-*HLA-DQA2* expression group, whereas the activated CD8T_MHCII subtype following immunotherapy was enriched predominantly in the group with low *HLA-DQA2* expression (Fig. 3c). This pattern was also detected at the individual patient level, where *HLA-DQA2* expression in DCs inversely correlated with the proportion of CD8T_MHCII cells (R = -0.55; *P* = 0.044) and positively correlated with that of CD8T_*TNFSF9* cells (R = 0.69; *P* = 0.007) (Supplementary Fig. S4a, b).

Considering that bystander CD8^+^ T cells can also undergo expansion following immunotherapy, we focused on tumor-reactive CD8^+^ T cells, which are thought to share TCR clonotypes with the Tex subset^25,45^. We defined these non-terminally exhausted tumor-reactive CD8^+^ T cells as Tex-Shared (Tex-S) cells, which included precursor exhausted CD8^+^ T (Tpex) cells as well as some early-stage CD8^+^ T cells (Fig. 3e). Together, Tex and Tex-S cells constitute the tumor-reactive CD8^+^ T cell population. Although the composition of Tex-S differed between the two groups—primarily because of the enrichment of CD8T_MHCII in the group with low *HLA-DQA2* expression and CD8T_*TNFSF9* in the group with high *HLA-DQA2* expression — CD8T_MHCII remained the main contributor to Tex-S in both groups and likely represents Tpex (Fig. 3f). As expected, the proportion of Tex-S cells increased significantly following immunotherapy in the low-*HLA-DQA2* expression group (*P* < 0.0001), whereas in the high-expression group, the proportion of Tex-S cells remained relatively stable across the baseline, post-radiotherapy, and post-immunotherapy stages, with the abundance of Tex cells decreasing after treatment (Fig. 3g; Supplementary Fig. S4c). Similarly, hyperexpanded clonotypes were also enriched in the low-expression group after therapy (Fig. 3h). Collectively, these results indicate that clonal expansion is more pronounced in patients with low *HLA-DQA2* expression.

We next performed an in-depth analysis of paired scTCR-seq data. TCR k-mer analysis was conducted to examine the CDR3 sequence features across different CD8^+^ T cell populations. Tumor-reactive CD8^+^ T cells were enriched in short k-mers, such as KL, RA, and EK, which contain hydrophobic and charged residues, reflecting flexible motifs likely involved in tumor antigen recognition. In contrast, bystander CD8^+^ T cells were enriched in longer k-mers, including NTEAFF, NTEA, and NTEAF, which often contain polar or aromatic residues, suggesting that these motifs are structural or public and lack direct tumor specificity (Fig. 3i) (see Methods).

Additionally, tumor-reactive CD8^+^ T cells in the group with low *HLA-DQA2* expression exhibited lower TCR sequence similarity between pre-treatment and post-immunotherapy time points, as measured by both the Bray‒Curtis similarity index (0.02) and the Morisita index (0.01) (Fig. 3j) (see Methods for the calculations). In contrast, in the group with high *HLA-DQA2* expression, tumor-reactive CD8^+^ T cells after immunotherapy were more likely to be derived from tumor-reactive cells already present in the pre-treatment TME, likely reflecting their prior local expansion and proportional dominance before therapy. This pattern was validated at the individual patient level (*P* = 0.067) (Fig. 3k). Notably, P1, a CR patient with high *HLA-DQA2* expression, displayed a relatively similar TCR composition before and after treatment, whereas P9, a PR patient with low *HLA-DQA2* expression, showed markedly different TCR repertoires before and after therapy. These observations suggest that stratification on the basis of *HLA-DQA2* expression may better capture the underlying biology, given that numerous factors influencing whether a patient achieves CR or PR cannot be fully accounted for. Interestingly, we observed a similar phenomenon in an independent cohort of triple-negative breast cancer (TNBC) patients treated with combined radiotherapy and immunotherapy^46^ (Supplementary Fig. S4d).

### Tumor-reactive CD8^+^ T cells derived from PBMCs exhibit an enhanced effector function

**Fig. 4:**
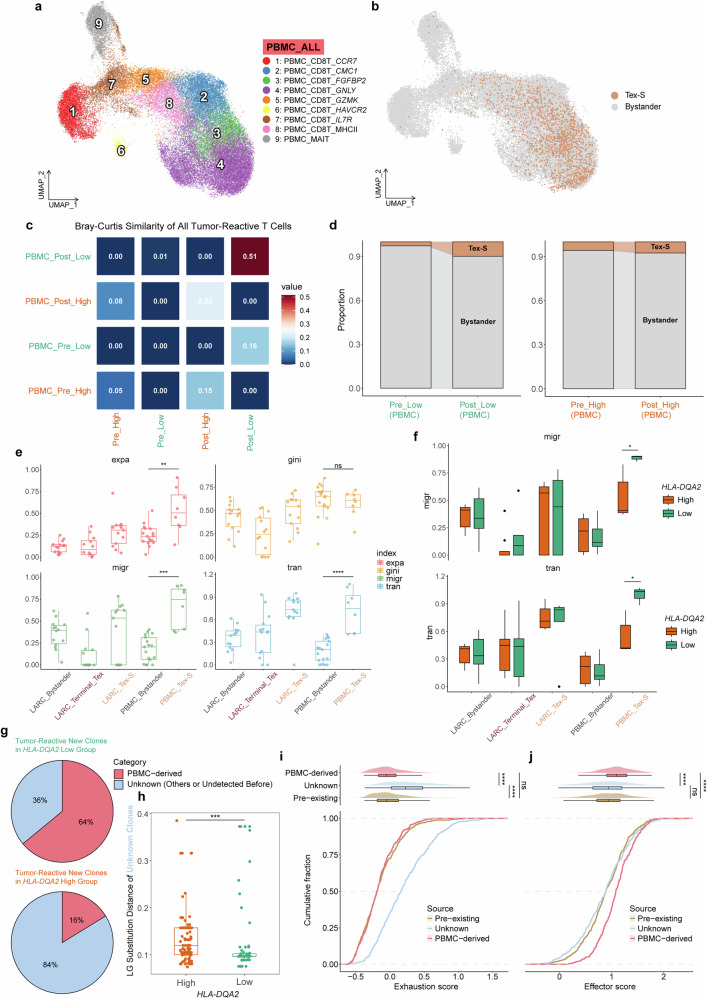
Tumor-reactive CD8^+^ T cells derived from PBMCs exhibit enhanced effector function. **a** UMAP projection of CD8^+^ T cells from all the PBMC samples. **b** UMAP projection of Tex-shared CD8^+^ T cells (Tex-S cells; sharing the same TCR clonotypes as Tex cells), and bystander CD8^+^ T cells from all the PBMC samples. **c** Heatmap showing the Bray–Curtis similarity indices between tumor-reactive CD8^+^ T cells from LARC tissues and PBMCs in the low- and high-*HLA-DQA2* groups before and after treatment. **d** Bar plot showing changes in the proportions of tumor-reactive CD8^+^ T cells in peripheral blood across the Pre and Post groups with low and high *HLA-DQA2* expression. **e** Boxplots showing the expansion (expa), Gini, migration (migr), and transition (tran) indices of Tex, Tex-S, and bystander CD8^+^ T cells from LARC tissues and PBMCs, with significance assessed for PBMC-derived Tex-S cells and bystander CD8^+^ T cells. **P < 0.01, ***P < 0.001, ****P < 0.0001, as determined as determined by the Wilcoxon rank-sum test. **f** Boxplots comparing the migration (migr) and transition (tran) indices of tumor-reactive and bystander CD8^+^ T cells from LARC tissues and PBMCs between the low- and high-*HLA-DQA2* groups. *P < 0.05, as determined as determined by the Wilcoxon rank-sum test. **g** Pie charts showing the origins of newly emerged tumor-reactive TCR clonotypes in the low-*HLA-DQA2* (top) and high-*HLA-DQA2* (bottom) groups, with PBMC-derived clonotypes shown in red and Unknown (other tissue sources or not captured by sequencing) clonotypes shown in blue. **h** Boxplots comparing Le and Gascuel substitution distances (see Methods) between Unknown TCR clones and PBMC clones in the low- and high-*HLA-DQA2* groups. ***P < 0.001, as determined as determined by the Wilcoxon rank-sum test. Boxplots and cumulative density plots comparing exhaustion scores (**i**) and effector scores (**j**) among Preexisting, Unknown, and PBMC-derived tumor-reactive CD8^+^ T cells. ****P < 0.0001, as determined as determined by the Wilcoxon rank-sum test and FDR correction.

As mentioned previously, in the group with high *HLA-DQA2* expression, tumor-reactive CD8^+^ T cells that expanded following immunotherapy originated primarily from the pre-treatment TME, whereas in the low-expression group, post-treatment tumor-reactive CD8^+^ T cells showed low similarity to pre-treatment TCR clonotypes. We therefore hypothesized that these new TCR clonotypes in the low-expression group were derived primarily from PBMCs. CD8^+^ T cells from PBMCs were classified into nine subtypes, and paired scTCR-seq analysis allowed us to identify potential tumor-reactive Tex-S CD8^+^ T cells in the peripheral blood whose composition was similar between the two groups (Fig. 4a, b; Supplementary Fig. S4e). As expected, in the low-*HLA-DQA2* expression group, tumor-reactive CD8^+^ T cells in the post-treatment TME displayed high similarity to Tex-S cells in PBMCs (Bray‒Curtis similarity index = 0.51), even though the baseline similarity was essentially negligible (Bray‒Curtis similarity index = 0.00) (Fig. 4c). This likely reflects the scarcity of exhausted CD8^+^ T cells and the lack of pre-existing tumor-reactive CD8^+^ T cells in the TME of the low-expression group before treatment. In contrast, Tex-S cells among PBMCs from the high-expression group remained relatively stable before and after therapy (Fig. 4d).

Next, we applied STARTRAC^47^ to monitor the cross-tissue migration capacity of tumor-reactive CD8^+^ T cells before and after therapy in the two groups. Compared with bystander CD8^+^ T cells, these tumor-reactive Tex-S cells exhibited greater expansion, migration, and transition indices (*P* < 0.01) in PBMCs (Fig. 4e). Notably, at the individual patient level, Tex-S cells in the low-*HLA-DQA2* expression group displayed significantly higher migration and transition indices than those in the high-expression group (*P* < 0.05) (Fig. 4f), even though their expansion indices were comparable (Supplementary Fig. S4f). These findings suggest that although peripheral CD8^+^ T cells in the low-expression group highly expressed various naïve markers prior to treatment (Supplementary Fig. S4g), they acquired a stronger ability to migrate into the tissue microenvironment and differentiate into tumor-reactive CD8^+^ T cells during therapy. In contrast, CD8^+^ T cells from patients in the high-expression group, despite exhibiting greater baseline peripheral immune inflammation, demonstrated a weaker response and mobilization capacity upon treatment.

On the basis of the origin of tumor-reactive CD8^+^ T cells, we classified all post-immunotherapy tumor-reactive CD8^+^ T cells in the TME into three groups: Pre-existing, PBMC-derived, and Unknown. Pre-existing cells are CD8^+^ T cells carrying TCR clonotypes already present in the pre-treatment TME, whereas PBMC-derived cells are CD8^+^ T cells carrying TCR clonotypes detected in PBMCs either before or after treatment (Fig. 4g). The Unknown category includes potential contributions from other tissues and TCR clonotypes that were not captured in the pre-treatment TME or PBMCs. To characterize these Unknown clones, we employed the Le and Gascuel substitution model to measure their evolutionary distance from known PBMC clones. Unknown clones in the high-*HLA-DQA2* expression group exhibited significantly greater evolutionary distances from PBMCs (*P* < 0.001) (Fig. 4h), suggesting that they are more likely derived from other tissues or TCR clonotypes not captured in the pre-treatment TME. In contrast, Unknown clones in the low-expression group were more likely to represent TCR clonotypes not detected in PBMCs. We then compared the functional profiles of the three groups of TCR origin and found that, although Pre-existing and PBMC-derived tumor-reactive CD8^+^ T cells displayed comparable exhaustion scores (Fig. 4i), PBMC-derived tumor-reactive CD8^+^ T cells exhibited significantly higher effector scores, indicating stronger antitumor activity (*P* < 0.0001) (Fig. 4j).

Overall, these observations suggest that when the TCR clonotypes that expand significantly after immunotherapy are derived primarily from pre-treatment intratumoral CD8^+^ T cell pools, particularly those already partially expanded under the chronic state of inflammation, as observed in patients with high *HLA-DQA2* expression, they may contribute to an immune-tolerant state. This may be because the tumor antigens recognized by these pre-existing TCR clonotypes have already triggered immune evasion or are suboptimal for eliciting a robust cytotoxic response, thereby limiting their effectiveness in tumor clearance. This mechanism may also explain the poor response of pMMR/MSS CRC patients to immunotherapy alone, in which expansion is limited mainly to ineffective pre-existing CD8^+^ T cell clones, resulting in an insufficient generation of novel, tumor-reactive populations. We define this phenomenon as clonal entrapment, a state in which, during sequential radiotherapy and immunotherapy, pre-existing local inflammation drives the post-treatment expansion of TCR clonotypes that originate predominantly from the pre-treatment TME, whereas chronic inflammation in PBMCs restricts the migration and transition of potentially tumor-reactive peripheral CD8^+^ T cells, ultimately resulting in suboptimal treatment responses.

### Residual and treatment-resistant tumor cells exhibit increased GDF15 expression

Next, we sought to investigate the second question: whether resistant tumor cells use specific mechanisms to suppress the infiltration of peripheral CD8^+^ T lymphocytes, thereby exacerbating clonal entrapment. First, we calculated a Tumor_score and a Normal_score for each epithelial cell and subtracted the Normal_score from the Tumor_score (Methods). Moreover, we used the R package inferCNV to infer copy number variations (CNVs) in each epithelial cell. Tumor cells were defined as those exhibiting high tumor scores in conjunction with apparent CNVs (Supplementary Fig. S5a, b). Despite removing batch effects prior to epithelial cell integration, these tumor cells still exhibited substantial inter-patient heterogeneity. To assess the overall chromosomal instability (CIN) status of each sample quantitatively, we integrated a published WES dataset of LARC patients^48^ and calculated the fraction of the genome altered (FGA) both before and after treatment. Interestingly, tumors that exhibited decreased CIN levels after treatment generally had higher baseline CIN levels prior to therapy (*P* < 0.001), suggesting a potential adaptive mechanism in which tumor cells with a higher CIN were selectively eliminated, while those with a lower CIN, which are potentially resistant to therapy, persisted (Supplementary Fig. S5c–e). Although higher levels of tumor aneuploidy have been associated with worse prognosis and immune evasion in several cancers, such as melanoma, following immunotherapy^49,50^, findings suggest that in the context of combined radiotherapy and immunotherapy, patients with lower immunogenicity and greater aneuploidy may actually have better outcomes^51^.

**Fig. 5:**
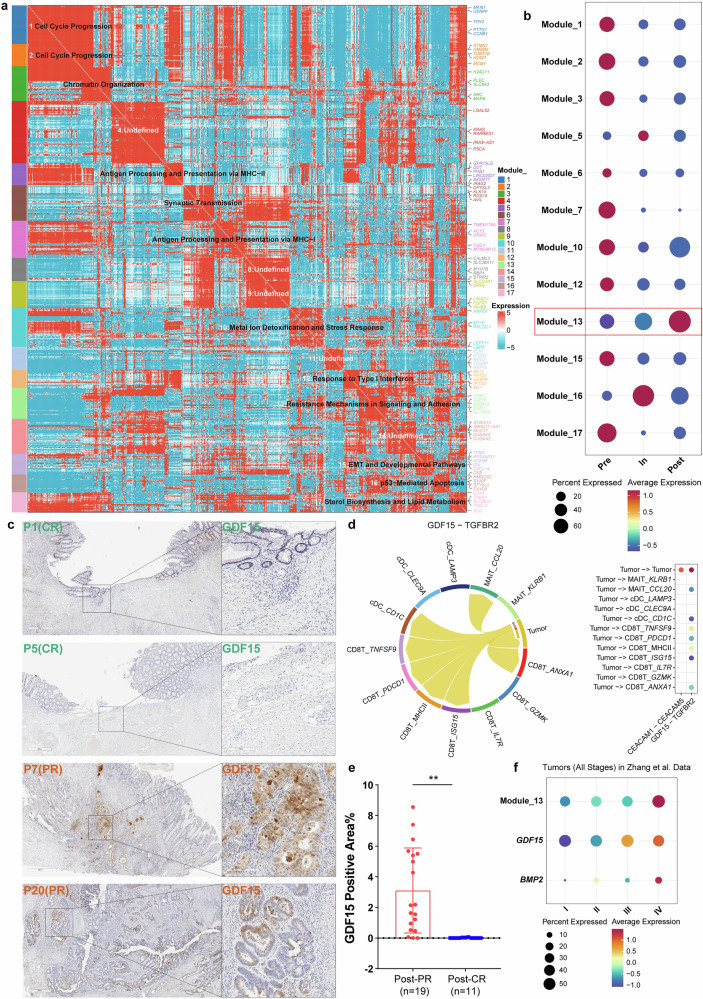
Residual and treatment-resistant tumor cells exhibit upregulated GDF15 expression. **a** Heatmap showing the 17 coexpression gene modules identified in tumor cells. Each module and its representative genes are labeled. **b** Bubble plot showing module expression in tumor cells before treatment (Pre), after radiotherapy (In), and after immunotherapy (Post). **c** GDF15 staining of tumor tissues acquired after immunotherapy from representative patients who achieved a CR (P1 and P5) and patients who achieved a PR (P7 and P20). Scale bars: 700 μm (left), 200 μm (right). **d** Chord diagram (left) and bubble plot (right) showing cell–cell interactions between tumor cells, DC subtypes, and CD8^+^ T cell subtypes. **e** The GDF15-positive area (%) determined by IHC staining was quantified using ImageJ. The data are presented as the mean ± SD. **P < 0.01, two-tailed unpaired Student’s *t*-test. **f** Bubble plot showing the expression of Module_13 and its representative genes, *GDF15* and *BMP2*, in tumor cells from public datasets. I–IV represent sampling time points from pre-treatment (I) to post-immunotherapy (IV).

Gene co-expression modules in tumor cells were then analyzed using Hotspot^52^. Ultimately, we identified 17 gene expression modules and selected genes from each module with a Z score greater than 150, which reflect the autocorrelation strength between the gene and the module, for enrichment analysis. We displayed the top three significantly enriched pathways to define the function of each module. Five modules (Module_4, Module_8, Module_9, Module_11, and Module_14) were excluded from further discussion because the enriched pathways were not statistically significant (*P*.adjust > 0.05) or because no pathways were enriched (Fig. 5a; Supplementary Fig. S6). Among the remaining 12 modules, nine were detected in pre-treatment tumor cells only. These included Module_1 (*MKI67* and *PTTG1*) and Module_2 (*MCM7* and *STMN1*), which are associated with cell cycle progression; Module_3 (*H2AC11* and *H3C10*), which is associated with chromatin organization; and the well-characterized Metal module (Module_10), Interferon module (Module_12), and pEMT module (Module_15)^53^, all of which were no longer detectable after radiotherapy (Fig. 5b).

Following the completion of radiotherapy, the expression of two modules, antigen processing and presentation via MHC-II (Module_5) and p53-mediated apoptosis (Module_16), was upregulated in tumor cells. These findings suggest that radiotherapy activated the p53 pathway by inducing DNA damage, triggering programmed cell death in tumor cells and inducing an immune-inflamed response that increased MHC-II expression in tumor cells. This increase in immunogenicity may synergize with subsequent immunotherapy to promote anti-tumor effects. However, following immunotherapy, the expression of these two modules decreased in the remaining tumor cells, whereas Module_13 emerged as the dominant module. Since the expression of Module_13 increased after radiotherapy, we defined it as a drug resistance gene module expressed by tumor cells. Enrichment analysis of genes within this module revealed that tumor cells may adapt to treatment pressure by promoting angiogenesis (placenta blood vessel development), maintaining cell adhesion (hemidesmosome assembly), activating pro-survival signaling pathways (response to peptide hormone), acquiring stem cell-like properties (endoderm formation), and regulating their proliferation (regulation of epithelial cell proliferation) (Fig. 5b; Supplementary Fig. S6).

Here, we found that growth differentiation factor 15 (GDF15) and CEACAM1 were the two proteins with the highest Z-scores among those involved in intercellular communication within Module_13 (Fig. 5a). Notably, cell–cell communication analysis revealed that only GDF15 interacted with immature cDC_*CD1C* cells as well as multiple CD8^+^ T cell subtypes (Fig. 5c, d). Recently, GDF15 was identified as a key mediator of immunotherapy failure in patients with non-small cell lung cancer and urothelial cancer^54^, and it may similarly contribute to treatment resistance in patients with LARC. Tumor cell-derived GDF15 inhibits DC maturation, suppresses T lymphocyte activation and recruitment, and contributes to poor prognosis^55^. We collected tissue samples from the LARC cohort (*n* = 20) in our study and from an additional cohort at Peking University Cancer Hospital (*n* = 10) following radiotherapy combined with immunotherapy. GDF15 was highly expressed in residual tumor cells from the PR group only (*P* < 0.01) (Fig. 5e). Moreover, we observed a similar pattern in the dataset from Zhang et al.: as treatment progressed, the expression of *GDF15* and *BMP2* (which is also a member of the transforming growth factor-beta (TGF-β) superfamily) and that of Module_13 steadily increased in the remaining tumor cells, supporting their potential role in the development of resistance to immunotherapy (Fig. 5f).

### GDF15 inhibits CD8^+^ T cell infiltration and reduces the efficacy of combined radiotherapy and immunotherapy

**Fig. 6:**
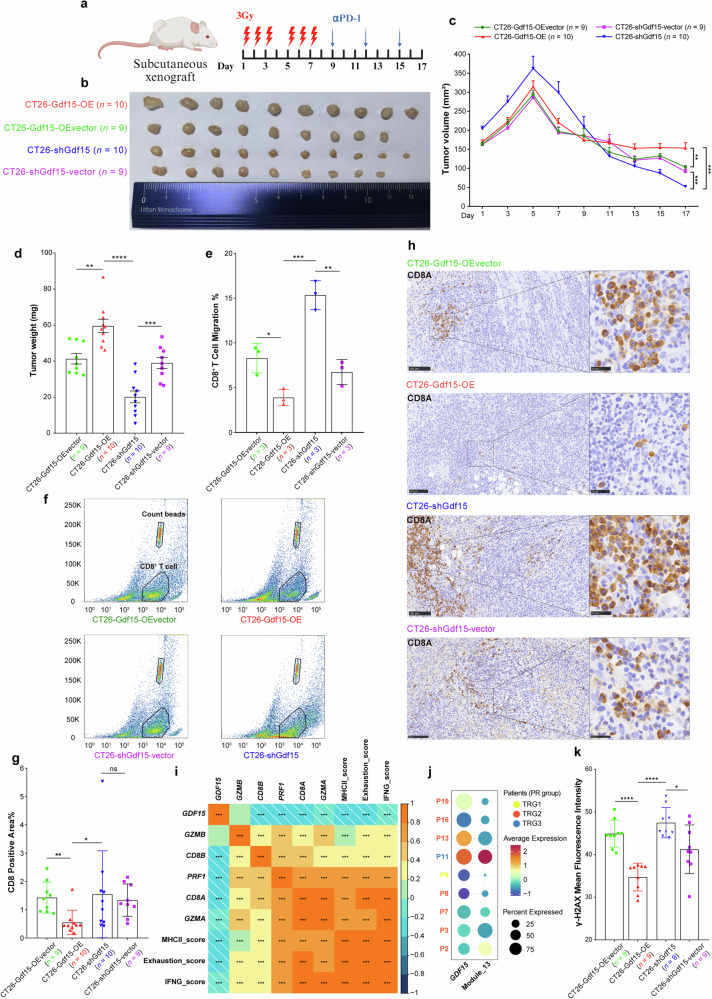
GDF15 inhibits CD8^+^ T cell infiltration and reduces the efficacy of combined radiotherapy and immunotherapy. **a** Schematic of the subcutaneous xenograft tumor model and treatment schedule combining long-term radiotherapy with immunotherapy. **b** Macroscopic view of subcutaneous xenograft tumors after combined long-term radiotherapy and immunotherapy. **c** Growth curves of subcutaneous xenografts in mice during treatment. The data are shown as the mean ± SEM. ****P < 0.0001, as determined by the Wilcoxon rank-sum test. **d** Tumor weights of the subcutaneous xenografts after treatment. The data are shown as the mean ± SEM. **P < 0.01, ***P < 0.001, ****P < 0.0001, two-tailed unpaired Student’s *t*-test. **e** CD8^+^ T-cell migration (%) in Transwell assays using conditioned media from CT26-Gdf15-OE cells, CT26-shGdf15 cells, and their respective controls. **f** Representative flow cytometry density plots of lower-chamber events (x-axis: SSC-A; y-axis: FITC-A) gated on CFSE^+^ CD8^+^ T cells; counting beads were used for normalization. **g** The CD8A-positive area (%) determined by IHC staining was quantified using ImageJ. The data are shown as the mean ± SD. ns, *P* > 0.1, *P < 0.1, **P < 0.01, two-tailed unpaired Student’s *t*-test. **h** CD8A staining of mouse tumor tissues from the CT26-Gdf15-OE, CT26-shGdf15, or their respective control groups after treatment. Scale bars: 100 μm (left), 25 μm (right). **i** Heatmap showing correlations between *GDF15* expression and CD8^+^ T cell marker genes, along with related GSVA scores, in the CRC bulk RNA-seq public dataset. ***P < 0.001, Spearman correlation analysis. **j** Bubble plot showing the expression of *GDF15* and Module_13 in tumor cells from each patient in the PR group after treatment. **k** The mean fluorescence intensity of three cell lines in nine different regions (100× magnification) was quantified using ImageJ. The data are shown as the mean ± SD. *P < 0.1, ****P < 0.0001, two-tailed unpaired Student’s *t*-test.

To investigate the role of GDF15 in mediating LARC tumor resistance, we engineered the murine CRC cell line CT26 to overexpress or knockdown GDF15 (CT26-Gdf15-OE and CT26-shGdf15, respectively) (Supplementary Fig. S7a–c). Using these cell lines, we established a subcutaneous xenograft tumor model in mice and administered a long-term radiotherapy regimen combined with immunotherapy (Fig. 6a). Sequential radiotherapy and immunotherapy were effective across all three groups of mice (Fig. 6b, c). However, tumors in the GDF15 overexpression group exhibited the most significant resistance to treatment (*P* < 0.001), whereas tumors with GDF15 knockdown demonstrated the most pronounced regression after combination therapy (Fig. 6d). Furthermore, immunohistochemistry revealed that, compared with that in the other groups, the GDF15 expression in residual tumor cells in the GDF15-overexpressing group remained high (Supplementary Fig. S7d, e).

Previous studies and our analysis of public CRC datasets revealed that high expression of GDF15 is significantly negatively correlated with CD8^+^ T cell infiltration in the TME and with the expression of various effector and exhaustion markers in CD8^+^ T cells (Fig. 6i). Consistent with these findings, our CFSE-based Transwell migration assay demonstrated that tumor-derived GDF15 suppressed CD8^+^ T-cell chemotaxis (Fig. 6e, f; Supplementary Fig. S7f). To further investigate this, we examined CD8^+^ T cell infiltration in tumor tissues from different groups of mice following treatment. Compared with the control group, the GDF15 overexpression group exhibited significantly lower CD8⁺ T cell infiltration (*P* < 0.01) (Fig. 6g, h). Although some mice with GDF15-knockdown tumors exhibited increased CD8^+^ T cell infiltration, overall, there was no significant difference between the GDF15 knockdown and control groups. One possible explanation is that the GDF15 knockdown group had the lowest tumor burden after treatment, resulting in a reduced inflammatory response in the TME. Alternatively, these tumor cells may retain additional resistance mechanisms. Notably, in the PR group, the TRG 3 patient (P11) presented the highest expression of GDF15 and Module_13, whereas the TRG 1 patient (P09) presented lower expression levels of both (Fig. 6j). These findings suggest that in patients such as P09, other tumor resistance mechanisms or interacting pathways may be involved, which warrants further investigation.

In vitro, compared with overexpressing cells, cells with knockdown of GDF15 presented a significant increase in the expression of the DNA damage marker γ-H2AX following exposure to 4 Gy of radiation (*P* < 0.0001) (Fig. 6k; Supplementary Fig. S7g), suggesting impaired DNA repair mechanisms. This impaired DNA repair capacity may lead to genomic instability, which is often associated with the accumulation of CNVs. This result is particularly relevant in light of our earlier findings, in which resistant tumor cells exhibited decreased CNV levels and increased GDF15 expression. The reduction in CNV levels in resistant cells may reflect a more stable genome, potentially maintained by GDF15, as tumor cells themselves may also express the GDF15 receptor TGFBR2, similar to CD8^+^ T cells (Fig. 5d).

To support the role of GDF15 in mediating resistance to radiotherapy, we analyzed two independent public LARC datasets. The GSE209746 dataset includes bulk RNA-seq data from the tumor tissues of 85 MSS LARC patients prior to radiotherapy^32^. We found that the patients with the poorest treatment response (TRG 3) exhibited the highest baseline expression of *GDF15* (Supplementary Fig. S7h). Additionally, recently published single-cell sequencing data on tumor tissues from MSS LARC patients before and after radiotherapy^56^ revealed that residual tumor cells post-radiotherapy had significantly increased expression of *GDF15* (*P* < 0.0001) (Supplementary Fig. S7i).

**Fig. 7:**
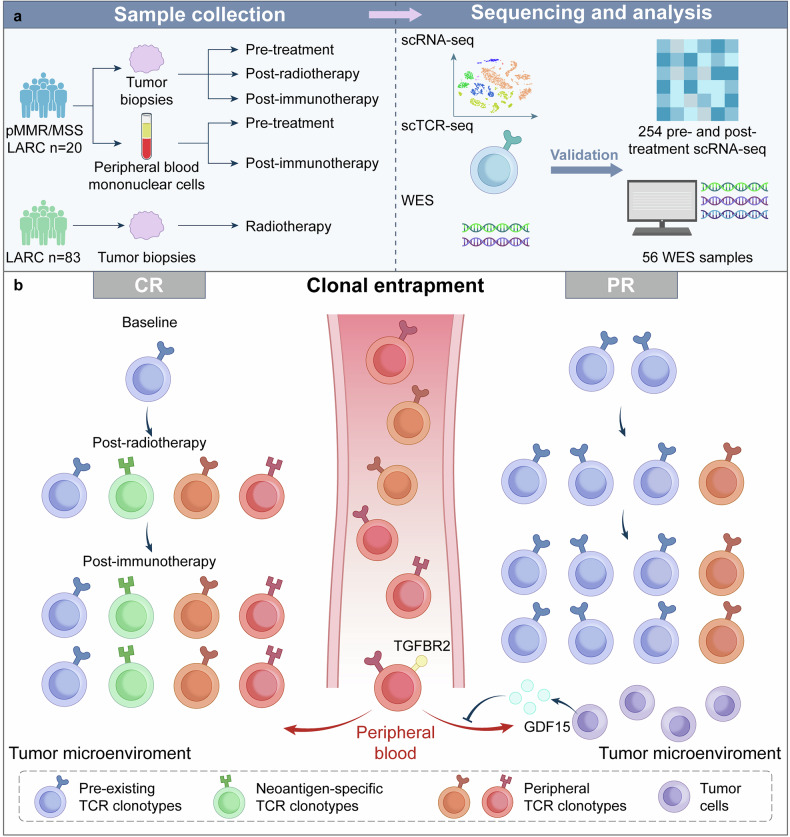
Single-cell atlas and mechanism of clonal entrapment. **a** Schematic diagram of the in-house data used in this study (left) and the public datasets used for validation (right). **b** Schematic diagram of the specific mechanism of clonal entrapment.

In summary, we established high-resolution, longitudinal single-cell transcriptomic atlases of tumor tissues and peripheral blood from LARC patients undergoing sequential radiotherapy and immunotherapy. Our profiling led to the identification that clonal entrapment is a key feature associated with limited therapeutic efficacy in patients with pMMR/MSS tumors — a state characterized by high baseline *HLA-DQA2* expression in DCs and *GDF15* expression in resistant tumor cells. Specifically, patients with elevated *HLA-DQA2* expression exhibit a pre-existing chronic inflammatory microenvironment characterized by the accumulation of mature DCs and terminally exhausted CD8^+^ T cells. This hyperactivated but functionally compromised state is correlated with the limited presence of the naïve DCs required to present tumor neoantigens. Furthermore, *GDF15* expression in resistant tumor cells is associated with reduced infiltration of potential peripheral tumor-reactive CD8^+^ T cells into the TME. Consequently, the immune response following ICI therapy is observed to be “entrapped”; that is, expansion is confined predominantly to pre-existing intratumoral TCR clonotypes already partially expanded under chronic inflammation, rather than novel, high-effector peripheral clones. This lack of recruitment and expansion of novel peripheral clones indicates restricted TCR repertoire diversification, characterizing the clinical resistance observed in these patients (Fig. 7a, b).

### Radiotherapy effectively reduces the abundance of THBS2^+^ CAFs, enabling immune infiltration during subsequent immunotherapy

Finally, we focused on the third question: The remodeling effects of radiotherapy on fibroblast subtypes and how these differ from those of chemotherapy-induced remodeling, given that chemoimmunotherapy remains one of the most commonly used neoadjuvant treatment strategies^57,58^. Radiotherapy effectively reduces the proportion of fibroblasts in the TME, which is important because cancer-associated fibroblasts (CAFs) promote the formation of “cold tumors” and contribute to tumor progression^59^. On the basis of marker gene expression, we classified all the fibroblasts in our dataset into the following 11 subtypes: Two normal fibroblast precursor subtypes (pFB_*COL15A1* and pFB_*PI16*), one myofibroblast subtype (myCAF_*WNT2*), three inflammatory fibroblast subtypes (iCAF_*AREG*, iCAF_*CXCL5*, and iCAF_*CXCL10*), and five other subtypes (including CAF_*SOX6* and CAF_*ADAMDEC1*, which are considered to be specific to the gastrointestinal microenvironment^60^) (Supplementary Fig. S8a, b).

Radiotherapy effectively reduced the proportion of or even eliminated the myCAF subtype (Cluster 9, myCAF_*WNT2*) in the TME, which has been associated with poor prognosis in CRC^61,62^. We further analyzed the expression of the myCAF marker gene *CTHRC1* and the pro-tumorigenic factors *WNT2* and *THBS2*, which are secreted by myCAFs, across the treatment timeline. The expression of these genes significantly decreased in the CR and PR groups after radiotherapy (*P* < 0.0001); however, baseline expression levels of these factors were higher in the PR group than in the CR group before treatment (Supplementary Fig. S8c). Following immunotherapy, CAFs were largely remodeled toward a normal fibroblast state, characterized by high expression of *ADAM28* and *ADAMDEC1*. These findings were corroborated by data from two public CRC scRNA-seq datasets (SMC and KUL), which included adjacent normal samples and tumor-normal junction samples^63^ (Supplementary Fig. S9b–d).

Given recent reports that *THBS2*^+^ CAFs contribute to chemotherapy resistance in the context of CRC^64^, we analyzed a scRNA-seq dataset from 29 patients with pMMR/MSS RC treated exclusively with chemotherapy, among which paired tumor samples were collected from 27 patients before and after treatment^38^. Moreover, we obtained pre- and post-chemotherapy LARC samples from two other patients (P26 and P28). We mapped the CAF subtypes from our dataset to these data and discovered that the previously identified myCAF_*WNT2* subtype could be further divided into two distinct populations: myCAF_*THBS2* (*CTHRC1*, *THBS2*, and *INHBA*) and myCAF_*WNT4* (*WNT4* and *WNT5A*), both of which express myCAF marker genes, including *ACTA2*, *TAGLN*, and *WNT2* (Supplementary Fig. S8d, e).

Comparative analysis revealed that chemotherapy significantly reduced the proportion of the myCAF_*WNT4* subtype (*P* < 0.0001) but had a minimal effect on the myCAF_*THBS2* subtype (*P* = 0.13). In contrast, radiotherapy significantly reduced the proportion of both subtypes (myCAF_*WNT4*: *P* < 0.0001; myCAF_*THBS2*: *P* < 0.0001) (Supplementary Fig. S8f, g), suggesting that radiotherapy is more effective than chemotherapy at targeting CAF populations associated with tumor progression and drug resistance, particularly the myCAF_*THBS2* subtype, and that this effect was consistently observed in both patients who achieved a CR and those who achieved a PR (Supplementary Fig. S8h, i; Supplementary Fig. S9a). We also compared the effects of radiotherapy and chemotherapy on endothelial cell dynamics. Radiotherapy more effectively induced the expression of *CD74* and various MHC-II molecules in endothelial cells, facilitating their conversion into non-professional antigen-presenting cells. The upregulation of adhesion molecules such as MADCAM1 and ICAM1 in endothelial cells after radiotherapy also effectively promoted lymphocyte infiltration into tumor tissue, providing a favorable environment for subsequent immunotherapy (Supplementary Fig. S9e, f).

## Discussion

Previous studies on basal cell carcinoma and lung cancer have reported a systematic analysis of the clonal architecture of tumor-specific CD8^+^ T cells following anti-PD-1 immunotherapy and have introduced the concepts of clonal replacement and clonal revival^24,25^. Clonal replacement refers to the emergence of entirely new tumor-specific CD8^+^ T cell clones after treatment that are not derived from pre-existing tumor-infiltrating CD8^+^ T cells. The expansion of peripheral T cells within the TME is associated with improved responses to ICI therapy^65^. In contrast, clonal revival proposes that both new peripheral clones and pre-existing intratumoral clones can be recruited into the tumor and become functionally active during treatment. Our study extends these concepts to combination radiotherapy followed by immunotherapy and proposes a refined interpretation of clonal revival. In patients who achieved a CR, CD8^+^ T cell clones that expanded in the tumor microenvironment after treatment were largely derived from newly recruited peripheral TCR clonotypes via radiotherapy-induced inflammatory cues and exhibited enhanced effector function. In contrast, the dominance of pre-existing TCR clonotypes in patients who achieved a PR may explain their suboptimal response, as these clonotypes likely targeted tumor antigens that had already undergone immune editing or immune escape prior to treatment. These findings highlight the essential role of novel TCR clonotype recruitment or generation in successful treatment responses and provide a mechanistic explanation for why patients with pMMR/MSS LARC typically do not benefit from immunotherapy alone: In the absence of radiotherapy, dominant CD8^+^ T cell expansion is confined to TCR clonotypes that already reside in the TME, which have limited opportunity for clonal diversification. However, radiotherapy induces inflammatory signals that promote the recruitment of novel peripheral TCR clonotypes or the generation of new tumor-specific clones within the TME, and subsequent immunotherapy can then selectively expand these newly introduced or generated T cell clones, thereby increasing anti-tumor efficacy and conferring benefits to patients with pMMR/MSS LARC.

In the current study, we introduce the concept of clonal entrapment to describe the phenomenon observed in patients with poor therapeutic responses following sequential radiotherapy and immunotherapy. This state appears to be characterized by two key factors: (1) pre-existing local immune inflammation within the TME, marked by an abundance of *HLA-DQA2*^+^ mature DCs and terminally exhausted CD8^+^ T cells, which correlates with a limited presence of naïve DCs capable of presenting tumor neoantigens; and (2) a chronic immune inflammatory environment in pretreatment PBMCs, which is associated with reduced migration and transition potential of tumor-reactive CD8^+^ T cells. Furthermore, *GDF15* secretion by resistant tumor cells is linked to a further reduction in the infiltration of these cells into the TME. Consequently, the recovery of functional tumor-reactive CD8^+^ T cells after immunotherapy involves predominantly pre-existing TCR clonotypes within the TME — particularly those already partially expanded under chronic inflammation — which exhibit weaker antitumor activity than PBMC-derived clones. This limited diversification of the TCR repertoire reflects the features of clonal entrapment, where the post-treatment microenvironment is characterized by insufficient recruitment or expansion of novel tumor-reactive CD8^+^ T cell clonotypes, potentially constraining the overall immune response. Of course, it is important to note that if radiotherapy was to directly eliminate all tumor cells, even patients with strong local and peripheral immune inflammation prior to treatment would still exhibit a good therapeutic response following combination therapy. However, these patients would inherently not require immunotherapy; therefore, they are beyond the scope of our discussion.

To further explain why only a subset of patients exhibited recruitment or expansion of novel tumor-reactive TCR clonotypes, we examined potential baseline determinants that may influence this process. Notably, lower expression of *HLA-DQA2* in dendritic cells (DCs) before treatment was consistently associated with favorable responses, both in our cohort and in multiple external cohorts of MSI-H CRC patients receiving ICI monotherapy. Interestingly, patients with higher DC expression of *HLA-DQA2* often exhibited a more pro-inflammatory peripheral blood immune profile at baseline. This pattern aligns with previous findings in viral infection models. Specifically, higher systemic *HLA-DQA2* expression is associated with milder or asymptomatic SARS-CoV-2 infections, highlighting the inherent inter-individual variability in *HLA-DQA2*–mediated immune regulation^43,44^.

However, in the context of cancer, our results revealed that patients with higher *HLA-DQA2* expression tended to have poorer responses to combined radiotherapy and immunotherapy than those with lower *HLA-DQA2* expression. This paradox may reflect a shift in *HLA-DQA2* function from facilitating antiviral immunity to promoting chronic or dysregulated immune activation, which may compromise the ability to mount an effective immune response following radiotherapy. In this scenario, radiotherapy-induced inflammatory cues may fail to efficiently generate new tumor-reactive TCR clonotypes, resulting instead in the expansion of pre-existing intratumoral clones, particularly those that have already undergone partial expansion under the chronic state of inflammation, with limited antitumor potential after ICI immunotherapy. This interpretation aligns with our findings in a separate cohort of LARC patients treated with radiotherapy but not immunotherapy, in which patients with the poorest pathological response (TRG3) paradoxically exhibited higher baseline levels of exhausted T cells and increased MHC class II antigen presentation activity than those with intermediate response (TRG2). These observations suggest that a hyperactivated but functionally compromised immune baseline state, characterized by high *HLA-DQA2* expression, may intrinsically constrain therapeutic efficacy by restricting the generation and expansion of novel, tumor-reactive T cell clones within the TME during treatment.

Large-scale genomic analyses involving thousands of patients with CRC have revealed that compared with patients with dMMR/MSI-H tumors, those with pMMR/MSS tumors exhibit significantly greater numbers of CNVs^66^. In our study, during combination therapy, CNV levels in residual, treatment-resistant tumor cells gradually decreased, which coincided with a progressive increase in *GDF15* expression at the transcriptomic level. Previous studies suggest that tumors with more CNVs are associated with worse prognosis and immune evasion following immunotherapy^49,50^. However, in the context of combined radiotherapy and immunotherapy, the results of our study and previous reports suggest that these cells may be more susceptible to elimination^51^. Furthermore, GDF15 suppresses CD8^+^ T cell infiltration and is associated with poor prognosis. Recent findings also indicate that targeting GDF15 reverses resistance to PD-1/PD-L1 blockade in other cancers^54,55^. In our study, the concomitant reduction in CNVs and upregulation of GDF15 expression in treatment-resistant tumor cells suggest a potential adaptive resistance mechanism in which tumors shift from a genomically unstable state to a more immune-evasive, GDF15-driven phenotype under therapeutic pressure. Regardless of whether GDF15 expression is pre-existing or induced by therapy, its persistent enrichment in residual lesions creates a sustained barrier to T cell infiltration. Thus, targeting GDF15 represents a strategic opportunity to dismantle this immune barrier and reverse clonal entrapment, ultimately increasing the efficacy of radiotherapy and immunotherapy combinations in patients with MSS LARC.

Notably, a key limitation of our study is that the cohort size was limited by the clinical reality that immune checkpoint inhibitors are not currently a first-line treatment for patients with pMMR/MSS LARC. As a result, the use of combined radiotherapy and immunotherapy remains limited by regulatory and financial barriers, thereby restricting the number of eligible participants. To address this limitation, we systematically integrated multiple published datasets to validate our findings. Another limitation is the lack of clinical evaluations of therapeutic strategies targeting GDF15. While our findings suggest that GDF15 may act as a mediator of treatment resistance, further functional studies are needed to formally dissect the underlying causal drivers. Additionally, dedicated clinical trials are needed to evaluate the efficacy and safety of GDF15-targeted combination therapies. Despite these limitations, our study provides a comprehensive longitudinal analysis of pMMR/MSS LARC patients receiving combined radiotherapy and immunotherapy, with multi-dimensional paired sampling of tumor tissues and peripheral blood across distinct treatment stages.

## Materials and methods

### Patient enrollment

A total of 22 patients aged 18–80 years with previously untreated rectal adenocarcinoma were enrolled between January 2023 and August 2024 at Peking University People’s Hospital. All patients were confirmed to have mismatch repair-proficient and microsatellite-stable (pMMR/MSS) tumors on the basis of professional pathological assessment. Magnetic resonance imaging (MRI) revealed the clinical stage as T2–T4bN + , with the distal tumor margin located within 10 cm of the anal verge. All participants had an Eastern Cooperative Oncology Group (ECOG) performance status of 0–2 and no known autoimmune or immune-related disorders.

### Ethics approval

All research involving human participants, human material, and clinical data in this study was performed in accordance with the Declaration of Helsinki. The study protocols were reviewed and approved by the Research and Ethics Committee of Peking University People’s Hospital and Peking Union Medical College Hospital. Specifically, the prospective cohort receiving long-term radiotherapy combined with immunotherapy (*n* = 22) was approved by the Research and Ethics Committee of Peking University People’s Hospital (Approval ID: 2023PHB222-001) and was registered at ClinicalTrials.gov (NCT06493240).

### Consent to participate

Written informed consent to participate was obtained from all participants in both cohorts prior to enrollment in accordance with institutional ethical guidelines.

### Treatment protocol and sample collection

Among the 22 participants enrolled in this study, 20 were assigned, on the basis of multidisciplinary team consultation, to receive long-term radiotherapy combined with immunotherapy (radioimmunotherapy). In contrast, the remaining two patients (P21 and P22) received chemotherapy alone. Prior to treatment initiation, tumor tissue samples were collected via colonoscopy, and 5 mL of peripheral blood was also collected. The radioimmunotherapy regimen included long-term radiotherapy (45–50.4 Gy in 25 fractions, administered five times per week for 5 weeks), followed by intravenous administration of the anti-PD-1 monoclonal antibody sintilimab (200 mg once a week for three weeks). One week after completing radiotherapy, a second tumor biopsy was performed via colonoscopy. Surgery was scheduled approximately eight weeks after radiotherapy, during which additional tumor tissue and 5 mL peripheral blood samples were collected. Collected tumor tissues were either enzymatically dissociated into single-cell suspensions or fixed in formalin and embedded in paraffin to generate formalin-fixed paraffin-embedded (FFPE) blocks. Moreover, 10 postoperative FFPE tumor samples from LARC patients treated with a similar radioimmunotherapy protocol at Peking University Cancer Hospital were included for validation.

Treatment efficacy was assessed through postoperative histopathological evaluation on the basis of the RECIST 1.1 criteria. The tumor regression grade (TRG) was determined according to the American Joint Committee on Cancer (AJCC) classification: TRG0 (complete response, no viable tumor cells), TRG1–3 (partial response with varying degrees of residual tumor cells), and TRG4 (no response). Moreover, tumor tissue samples from an additional cohort of 83 treatment-naïve patients with LARC, collected between 2018 and 2019 at Peking University People’s Hospital and Peking Union Medical College Hospital, were included in the bulk RNA-seq analysis. These patients underwent long-term radiotherapy combined with chemotherapy, and their TRG status was evaluated similarly after treatment.

### Whole-exome sequencing

Total DNA was extracted from LARC tumor tissue and peripheral blood samples using the QIAGEN DNeasy Blood & Tissue Kit (for fresh tissue and blood). The extracted DNA was fragmented using a Covaris M220 Focused-ultrasonicator to achieve optimal fragment sizes for sequencing. Sequencing libraries were constructed following standard protocols. Exome capture was performed using Human Exome 2.0 Plus (Twist Bioscience) according to the manufacturer’s recommended procedure. The final libraries were sequenced using paired-end (150 bp) reads on the Illumina NovaSeq 6000 sequencing system (Illumina). Sequencing was conducted at LC-Bio Technology Co., Ltd. (Hangzhou, China), ensuring high-quality data for analysis.

Prior to alignment, low-quality reads, including those containing sequencing adaptors or those with quality scores below 20, were removed using fastp. Retained reads were aligned to the hg19 reference genome using the Burrows–Wheeler Aligner (BWA). Picard tools were then used to identify and mark duplicate reads in the BAM file, followed by local realignment around the indels to correct alignment errors. Base quality score recalibration was performed to reduce systematic biases prior to variant calling. Somatic mutations were called using Mutect2, and the resulting VCF files were annotated with ANNOVAR. VCF files were converted into MAF files for downstream analysis, and maftools (v2.8.5) was used for further interpretation.

### Cell type enrichment across treatment stages

To assess the distributional preferences of various cell populations across treatment stages (pre-, in-, and post-treatment), we used a chi-square-based method adapted from the STARTRAC framework. We calculated the ratio of observed to expected cell proportions (*R*_*o/e*_) for each cell type in each treatment group^47^, thereby quantifying whether specific cell subsets were over- or under-represented relative to random expectation. The resulting *R*_*o/e*_ values were visualized as a heatmap to facilitate interpretation of temporal changes in cell composition during treatment. We defined five levels of enrichment on the basis of the *R*_*o/e*_ values: +++ (*R*_*o/e*_ > 1), indicating significant enrichment; ++ (0.8 < *R*_*o/e*_ ≤ 1); + (0.2 ≤ *R*_*o/e*_ ≤ 0.8); +/− (0 < *R*_*o/e*_ < 0.2); and − (*R*_*o/e*_ = 0), indicating complete depletion.

### Batch correction and integration of scRNA-seq data

To integrate single-cell RNA sequencing data across different batches or conditions, we used the Harmony algorithm^67^, which effectively corrects batch effects while preserving true biological variation. Harmony operates in low-dimensional principal component (PC) space, iteratively adjusting cell embeddings to align shared cell types across batches without requiring explicit matching. This method uses an objective function to simultaneously maximize clustering within biological groups and minimize separation by batch. Integrated embeddings generated by Harmony were subsequently used for downstream clustering analyses and visualization.

### Tissue dissociation and preparation of single-cell suspensions

Fresh tissue samples were transferred to Petri dishes containing ice-cold 1× PBS (free of RNase, Ca^2+^, and Mg^2+^), followed by thorough rinsing to remove blood, fat, and surface debris. The tissues were then minced into ~0.5 mm^2^ fragments and washed again with 1× PBS. For enzymatic digestion, tissue fragments were incubated at 37 °C for 20 min in a water bath with shaking (100 rpm) with digestion buffer containing 0.35% collagenase IV, 2 mg/mL papain, and 120 U/mL DNase I. Digestion was terminated by adding 1× PBS supplemented with 10% fetal bovine serum (FBS), and the suspension was gently triturated 5–10 times using a pipette to release single cells. The resulting cell suspension was sequentially filtered through 70 µm and 30 μm strainers and then centrifuged at 300 × *g* for 5 min at 4 °C. The cell pellets were resuspended in 100 μL of 1× PBS supplemented with 0.04% BSA. Red blood cells were lysed using 1 mL of 1× RBC lysis buffer (MACS, 130-094-183) for 2–10 min at room temperature or on ice. After lysis, the cells were centrifuged at 300 × *g* for 5 min at 4 °C and then resuspended in 100 μL of dead cell removal microbeads (MACS, 130–090–101), followed by incubation at room temperature for 15 min. Dead cells and debris were removed using an MS column (MACS, 130-042-201) according to the manufacturer’s protocol. The cell pellet was subsequently washed twice with 1× PBS containing 0.04% BSA by centrifugation at 300 × *g* for 5 min at 4 °C. Finally, single cells were resuspended in 100 μL of 1× PBS containing 0.04% BSA. Cell viability was assessed using the trypan blue exclusion method, and only samples whose viability was greater than 85% were retained. Cell counts were determined using a hemocytometer or an automated cell counter (Countess II Automated Cell Counter), with the final concentration adjusted to 700–1200 cells/μL.

### Single-cell library preparation and sequencing

Single-cell suspensions were processed using the Chromium Next GEM Single-Cell 5′ Kit v2 (10x Genomics) according to the manufacturer’s protocol. Approximately 8000 cells were loaded per channel onto a Chromium microfluidic chip to generate Gel Bead-In Emulsions (GEMs), where cell barcoding, reverse transcription, and cDNA tagging were performed in nanoliter droplets. Following droplet fragmentation and cDNA purification, amplification and library construction were performed according to the standard workflow, enabling the capture of cell-specific transcriptomes with unique molecular identifiers (UMIs). Libraries were sequenced on an Illumina NovaSeq 6000 platform (150 bp paired-end reads) by LC-Bio Technology (Hangzhou, China), targeting a minimum depth of 20,000 reads per cell to ensure sufficient transcript coverage for downstream analysis.

### Processing of the raw scRNA-seq data and quality control

The sequencing results generated by the Illumina platform were converted into FASTQ format using bcl2fastq (v5.0.1). FASTQ files were processed using CellRanger (v7.0.0; 10x Genomics) for alignment to the reference genome, transcript counting, and cell barcode assignment on the basis of the 5′ ends of the captured transcripts. The resulting matrices were imported into the Seurat package (v4.0.1) in R (v4.0.3) for downstream analysis. Basic quality control (QC) metrics for each sample are presented in Supplementary Table S3.

Rigorous QC procedures were applied to remove low-quality cells. Cells with fewer than 500 unique molecular identifiers (UMIs) or fewer than 300 detected genes were excluded. Moreover, cells with a high mitochondrial gene content, which is indicative of dying or damaged cells, were also excluded. To eliminate doublets, automated and manual filtering strategies were applied. The automated method used DoubletFinder (v2.0.3), which predicts doublets on the basis of artificial nearest neighbors^68^. Additionally, manual curation excluded cells co-expressing multiple lineage markers (e.g., *CD3E*^+^*CD3D*^+^ and *CD79A*^+^*MS4A1*^+^), which likely represent doublets formed by co-encapsulation of distinct cell types during droplet formation.

The gene expression matrices were subjected to log-normalization using NormalizeData, and highly variable genes were identified with FindVariableFeatures (nfeatures = 3000). Data were scaled using ScaleData, followed by dimensionality reduction with RunPCA. Principal components were used to construct neighborhood graphs and perform clustering with FindNeighbors and FindClusters, respectively. UMAP was then applied for two-dimensional visualization using RunUMAP.

Several samples, including the post-radiotherapy (P6_In and P12_In) and post-immunotherapy (P6_Post and P12_Post) samples from patients P6 and P12 and the post-immunotherapy (P19_Post) sample from patient P19, were excluded from subsequent analysis because of the extremely low or nearly zero number of epithelial cells detected. Since these patients did not achieve a clinical complete response (TRG 2), the absence of epithelial cells in their samples may have resulted from sampling error or an imbalance in cell populations during the sequencing process.

### Cell type annotation

Cell type annotation was performed manually on the basis of the canonical marker gene expression profiles. The lymphoid cells included B cells (*CD79A* and *MS4A1*), plasma cells (*MZB1* and *IGHG1*), CD4^+^ T cells (*CD4*), CD8^+^ T cells (*CD8A*), γδ T cells (*TRDC*), and NK cells (*NCAM1* and *GNLY*). Myeloid cells included mast cells (*CPA3* and *GATA2*), plasmacytoid dendritic cells (*IRF7* and *LILRA4*), conventional dendritic cells (*CD1C* and *LAMP3*), monocytes (*FCN1* and *VCAN*), macrophages (*CD68* and *C1QC*), and neutrophils (*G0S2* and *CSF3R*). Stromal cells included fibroblasts (*COL1A1* and *COL3A1*), pericytes (*RGS5*), smooth muscle cells (*MYH11*), and endothelial cells (*PECAM1* and *VWF*). Epithelial cells were identified on the basis of *EPCAM* and *KRT19* expression. Other populations included proliferating cells (*MKI67* and *STMN1*), hematopoietic stem-like cells (*CD34* and *SOX4*), and platelets (*PPBP*). Notably, hematopoietic stem-like cells (HSCs) and platelets were detected exclusively in peripheral blood samples, whereas epithelial and stromal cells were detected in tumor tissue samples only.

### Developmental trajectory analysis

We applied diffusion mapping using the R package destiny (v3.4.0) to infer dendritic cell (DC) maturation trajectories from the scRNA-seq data. Distances between DCs were calculated from their expression profiles to construct an affinity matrix, followed by eigendecomposition to generate diffusion components. This reduced-dimensional representation captured the continuous progression of DCs from the naïve state to the activated state.

### TRB k-mer analysis

TRB sequences were first divided into overlapping k-mers with lengths of 1–6 using a sliding-window approach, and for each sequence, the absolute counts and relative frequencies of all k-mers were calculated. The presence of a given k-mer in a sample was defined as the presence of at least one occurrence of that k-mer. To avoid excessively sparse features and ensure robust statistical inference, k-mers present in fewer than 2% of sequences were excluded from further analysis, and the remaining k-mers were assembled into a feature matrix. For each k-mer, we constructed a 2 × 2 contingency table and then fitted a univariate logistic regression model with the binary label as the outcome and the relative frequency of the k-mer (scaled by a factor of 10 to increase numerical stability) as the predictor, using a binomial family to calculate the odds ratio using the Haldane–Anscombe continuity correction with a 95% confidence interval. Statistical significance was determined using Fisher’s exact test when the expected count of any cell was < 5; otherwise, the Pearson chi-square test was employed. The estimated coefficient β was exponentiated to yield the odds ratio (OR) for that k-mer. Finally, *P* values across all k-mers were adjusted via the Benjamini–Hochberg procedure.

### Bray‒Curtis similarity index calculation

The Bray‒Curtis dissimilarity index indicates the difference in clonotypes between two groups, where a value close to 0 indicates high overlap (clonal expansion), and a value close to 1 indicates greater clonotypic diversity. Bray‒Curtis dissimilarity is calculated as follows:

$${{BCdis}}_{{ij}}=1-\frac{2{\sum }_{k=1}^{K}\min ({N}_{{ki}},{N}_{{kj}})}{{\sum }_{k=1}^{K}\left({N}_{{ki}}+{N}_{{kj}}\right)}$$

where ${N}_{{ki}}$ and ${N}_{{kj}}$ represent the number of clonotypes $k$ in groups $i$ and $j$, respectively, and $K$ denotes the total number of clonotypes in the corresponding groups. The Bray‒Curtis similarity ${{BCsim}}_{{ij}}$ is defined as follows.

$${{BCsim}}_{{ij}}=1-{{BCdis}}_{{ij}}$$

The Bray‒Curtis similarity index was calculated to compare the CD8^+^ T cell population of each group within our dataset and was validated using publicly available datasets.

### Morisita index calculation

To quantify the clonal overlap and diversity of CD8⁺ T cells before and after treatment, we calculated the Morisita index, a measure of clonality, using T-cell receptor (TCR) sequencing data. The Morisita index indicates the similarity of clonotypes between two samples, where values closer to 1 indicate greater overlap (clonal expansion), and values closer to 0 indicate greater clonotypic diversity. The calculation is based on the following function:

$${MorisitaIndex}=\frac{2\sum {p}_{i}\cdot {p}_{j}}{\left(\frac{\sum {p}_{i}^{2}}{{X}^{2}}+\frac{\sum {p}_{j}^{2}}{{Y}^{2}}\right)\cdot X\cdot Y}$$

where ${p}_{i}$ and ${p}_{j}$ represent the proportions of clonotypes in groups $i$ and $j$, respectively, and $X$ and $Y$ denote the total clonotype counts in the corresponding samples. The Morisita index was computed for each patient’s pre-treatment and post-treatment CD8^+^ T cell population within our dataset and validated using publicly available datasets.

### LG-based evolutionary distance determination

All TRB amino acid sequences of new tumor-reactive clones were aligned using ClustalW. To construct a comprehensive distance matrix, we estimated pairwise evolutionary distances using maximum likelihood estimation based on the Le and Gascuel amino acid substitution model. The evolutionary distance between a TRB amino acid sequence and PBMCs is defined as the minimum value of the evolutionary distance between the TRB amino acid sequence and all sequences from PBMCs. A greater evolutionary distance indicates a more distant phylogenetic relationship. Owing to the existence of identical TRB sequences, the evolutionary distance between PBMC-derived TRB and PBMCs is 0.

### Definitions of the Tumor_score and Normal_score

To calculate the Tumor_score and Normal_score for each epithelial cell, we downloaded COAD bulk RNA-seq data from the TCGA dataset and performed differential gene expression analysis using DESeq2 (v1.32.0). Genes with an adjusted *P* value < 0.05 were selected, and from these, the top 200 upregulated genes in both tumor and normal tissues were identified on the basis of the log_2_FoldChange value. These genes were subsequently used to construct two gene sets: one representing tumor tissue and the other representing normal tissue. The Tumor_score and Normal_score values for each epithelial cell were calculated using the AddModuleScore function in the Seurat package on the basis of the expression of the respective gene sets. The diff_score was obtained by subtracting the Normal_score from the Tumor_score. To address potential skewness caused by extreme values, the diff_score was normalized by setting thresholds at the top and bottom 10th percentiles (q1 and q2) and adjusting values below q1 to q1 and those above q2 to q2.

### CNV analysis

To identify genomic alterations at single-cell resolution and gain insight into tumor cell heterogeneity and its potential role in treatment resistance, we used the infercnv package (v1.8.1) to infer large-scale CNVs from the scRNA-seq data. The CNV score was calculated by subtracting 1 from the inferCNV matrix, squaring the result, computing the average change in CNV per cell across all genes, and multiplying by the total number of genes. To account for substantial variability in CNV alterations among tumor cells and potential skewness from extreme values, CNV scores were normalized by setting thresholds at the top and bottom 20th percentiles (q1 and q2). The values below q1 were set to q1, and those above q2 were adjusted to q2 to ensure a more consistent representation of CNV distribution across all cells.

### Hotspot-based identification of gene expression modules in tumor cells

To investigate gene programs associated with therapeutic resistance in tumor cells, we applied Hotspot (https://github.com/yoseflab/hotspot), a graph-based statistical framework designed to identify genes whose expression patterns exhibit significant local structure across a cell–cell similarity graph^69^. In this context, Hotspot operates under the assumption that genes varying in a spatially or transcriptionally coherent manner across neighboring cells are more likely to reflect biologically meaningful programs. The input graph was constructed using tumor cells derived from PCA embeddings, and genes were selected on the basis of the FindVariableFeatures output from Seurat and further filtered to retain genes whose average expression was greater than 0.01. This approach ensured a sufficient expression signal while minimizing noise from sparsely expressed genes. Hotspot first quantifies the degree of local autocorrelation for each gene across the neighborhood graph and then organizes genes that exhibit significant variability into co-expressed modules on the basis of pairwise expression similarity. These modules were subsequently used to characterize transcriptional programs that may be involved in therapeutic resistance.

### Cell culture and transfection

The murine colorectal cancer cell line CT26 was obtained from the American Type Culture Collection (ATCC) and maintained in the recommended medium (Gibco) supplemented with 10% fetal bovine serum (FBS) and 1% penicillin–streptomycin. Full-length murine PL-Gdf15 and LV-Gdf15-RNAi plasmids were custom designed and synthesized by GeneChem (Shanghai, China). Lentiviruses carrying either the target constructs or empty vector controls were produced by co-transfecting 293 T cells using the Lenti-Pac™ HIV Expression Packaging Kit (GeneCopoeia, Rockville, USA). Viral supernatants were collected 48 h post-transfection and filtered through a 0.45 μm membrane.

To generate GDF15-overexpressing and GDF15-knockdown CT26 cell lines, CT26 cells were infected with the respective viral supernatants in the presence of 10 μg/mL polybrene for 72 h, followed by 24-h of recovery in fresh culture medium. Stable clones were selected using 2 μg/mL puromycin over an additional 72 h.

### RNA isolation and quantitative RT-PCR

Total RNA was extracted from cells using TRIzol reagent (Thermo Fisher, Waltham, USA) according to the manufacturer’s instructions. Complementary DNA (cDNA) was synthesized, and quantitative real-time PCR (qRT-PCR) was performed using SYBR Green Supermix (Bio-Rad, Delaware, USA). GAPDH was used as the internal reference gene for normalization. Relative gene expression levels were calculated using the ΔΔCt method, with values normalized to those of the control group.

### Western blotting

Cellular proteins were extracted using standard lysis procedures and separated by SDS‒PAGE. The proteins were transferred to membranes and detected using enhanced chemiluminescence. The primary antibodies used included anti-mouse GDF15 (Rockland, Limerick, USA; 1:2000) and anti-mouse β-actin (Proteintech, Wuhan, China; 1:5000), with β-actin serving as a loading control.

### Animal models

All animal experiments were approved by the Institutional Animal Care and Use Committee of Peking University People’s Hospital (Approval No. 2025PHE052). Male BALB/c mice (4–6 weeks old) were purchased from HFK Bioscience (Beijing, China). GDF15-overexpressing, GDF15-knockdown, or parental CT26 cells (8 × 10^6^ cells per mouse) mixed with 25% (v/v) Matrigel (Corning, New York, USA) were subcutaneously injected into the mice. Once the tumors were established, the mice received localized irradiation using an RS 2000 Pro irradiator (Rad Source, Buford, USA) following a long-term radiotherapy protocol: 3 Gy each day and six sessions per week, with a one-day interval. Non-tumor-bearing areas were protected using lead shielding. An anti-PD-1 monoclonal antibody (10 mg/kg, i.p.) was administered starting one day after radiotherapy and administered again every three days for a total of three doses. Tumor growth was monitored every two days using digital calipers, and tumor volume was calculated using the following formula: volume = (length × width²)/2. Mice were euthanized by carbon dioxide inhalation at the end of treatment or when the tumor length reached 1.5 cm. The tumors were removed, weighed and subjected to subsequent immunohistochemical analysis.

### Immunohistochemistry and multiplex immunohistochemistry

IHC and multiplex IHC (mIHC) staining were performed on FFPE tissue sections. Slides were deparaffinized in xylene and rehydrated through a graded ethanol series (100%, 100%, 95%, and 70%). Antigen retrieval was performed using heat-induced EDTA buffer, followed by blocking of nonspecific binding sites.

For IHC staining, sections were incubated with primary antibodies, including rabbit anti-human GDF15 (Abcam, Cat# ab206141, 1:100), rabbit anti-mouse GDF15 (Rockland, Cat# 600-401-B07, 1:2000), and rabbit anti-mouse CD8A (Abcam, Cat# ab217344, 1:4000), at room temperature for 1 h, followed by incubation with secondary antibodies and detection using DAB and hematoxylin. Whole-slide images were acquired using a slide scanning system (Shenzhen, China). For quantification, three randomly selected regions from each tissue sample were analyzed at 200× magnification, and areas with positive staining were quantified using ImageJ.

For mIHC staining, sections were sequentially incubated with the following primary antibodies: rabbit anti-human MAdCAM1 (Abcam, Cat# ab307734, 1:800), rabbit anti-human CD49D (Abcam, Cat# ab202969, 1:100), rabbit anti-human CD3 (Abcam, Cat# ab135372, 1:100), rabbit anti-human ADAM28 (Abcam, Cat# ab198902, 1:50), mouse anti-human CD31 (Abcam, Cat# ab9498, 1:1000), mouse anti-human α-SMA (Abcam, Cat# ab7817, 1:20000), and rabbit anti-human Thrombospondin 2 (GeneTex, Cat# GTX636411, 1:100). After each primary antibody incubation, the slides were treated with secondary antibodies and fluorescent dyes. Antigen retrieval and blocking were performed before incubation with each subsequent antibody. The nuclei were counterstained with DAPI as the final step. Stained slides were scanned using an Olympus VS200 and analyzed with OlyVIA (Version 3.3).

### DNA damage assay

GDF15-overexpressing and GDF15-knockdown CT26 cells (1 × 10^5^) were seeded in 6-well plates and incubated for 24 h. The cells were then irradiated with 4 Gy of irradiation using an RS 2000 Pro (Rad Source, Buford, USA). DNA damage was assessed 24 h later using a DNA damage assay kit with γ-H2AX immunofluorescence (Beyotime, Shanghai, China). Following fixation and blocking of nonspecific binding, the cells were incubated with a rabbit anti-mouse H2AX antibody at room temperature for 1 h. After the primary antibody was removed, the cells were incubated with an anti-rabbit Cy3 secondary antibody at room temperature for 1 h. The cells were then stained with DAPI for 5 min at room temperature. Each step was followed by three washes with PBS (3 min each). Cells were imaged under a laser confocal microscope (ZEISS LSM 800) at 100× magnification, and nine random regions were selected for calculation of the mean fluorescence intensity.

### Statistics

The statistical methods and thresholds used for each analysis are described in the Results section, figure legends, and Methods section. All the statistical analyses were performed using GraphPad Prism 8 or R (v4.0.3). The Wilcoxon rank-sum test was used to analyze differences between two groups. The Wilcoxon signed-rank test was used to assess differences between paired samples before and after treatment. For normally distributed data, a two-tailed unpaired Student’s *t*-test was used to compare groups. Data are presented as the means ± standard deviations (SDs) or means ± standard errors of the means (SEMs), unless otherwise specified. Statistical significance was defined as *P < 0.05, **P < 0.01, ***P < 0.001, and ****P < 0.0001, with “ns” indicating that the difference was not statistically significant (*P* > 0.05). For multiple comparisons, the false discovery rate (FDR) was controlled through appropriate statistical adjustments.

## Supplementary information

Supplementary Figures

Supplementary Tables

Clinical trial protocol

## Data Availability

The raw scRNA-seq and TCR sequencing data reported in this study have been deposited in the Genome Sequence Archive of the BIG Data Center at the Beijing Institute of Genomics, Chinese Academy of Sciences, under accession number HRA010884. The public scRNA-seq datasets used in this study include GSE236581, GSE205506, CNP0004138, and GSE246613; detailed information is provided in the relevant sections of the article. The bulk RNA-seq matrix used in this study is provided in Supplementary Table S4. The code used for analysis is available from the corresponding author upon reasonable request. The analysis software is described in the Methods section and Supplementary Table S5.
