# Author Correction: Longitudinal single-cell and TCR repertoire profiling characterizes clonal entrapment in patients with pMMR/MSS locally advanced rectal cancer

**DOI:** 10.1038/s41421-026-00920-6

**Published:** 2026-07-17

**Authors:** Zhichun Tang, Mingxuan Zhu, Shidong Zhao, Dazhi Pang, Yancheng Cui, Guole Lin, Aiwen Wu, Yilin Lin, Xinyang Jiang, Wei Zhang, Nan Xiong, Changjiang Yang, Caihong Wang, Shan Wang, Yingjiang Ye, Fan Bai, Zhanlong Shen

**Affiliations:** 1 Biomedical Pioneering Innovation Center (BIOPIC) and School of Life Sciences, Peking-Tsinghua Center for Life Sciences (CLS), State Key Laboratory of Metabolic Dysregulation & Prevention and Treatment of Esophageal Cancer, Peking University, Beijing, China; 2 Department of Gastroenterological Surgery, Peking University People’s Hospital, Beijing, China; 3 Laboratory of Surgical Oncology, Beijing Key Laboratory of Precision Diagnosis, Treatment and Translational Research for Colorectal Cancer, Peking University People’s Hospital, Beijing, China; 4 School of Basic Medical Sciences, Tsinghua University, Beijing, China; 5 Department of General Surgery, Peking Union Medical College Hospital, Chinese Academy of Medical Sciences & Peking Union Medical College, Beijing, China; 6 State Key Laboratory of Holistic Integrative Management of Gastrointestinal Cancers, Beijing Key Laboratory of Carcinogenesis and Translational Research, Unit III, Gastrointestinal Cancer Center, Peking University Cancer Hospital & Institute, Beijing, China; 7 Department of Thoracic Surgery, the First Affiliated Hospital of Fujian Medical University, Fuzhou, Fujian China; 8 Vanke School of Public Health, Tsinghua University, Beijing, China; 9 Peking University Beijing-Tianjin-Hebei Biomedical Pioneering Innovation Center, Tianjin, China; 10 Beijing Advanced Center of Cellular Homeostasis and Aging-Related Diseases, Institute of Advanced Clinical Medicine, Peking University, Beijing, China

**Keywords:** Colorectal cancer, Cancer therapeutic resistance

Correction to: *Cell Discovery* 10.1038/s41421-026-00900-w, published online 30 June 2026

In this article, we identified an oversight in Treatment protocol and sample collection in the Materials and methods section. The purpose of this modification is exclusively to rectify the dosing regimen and sampling schedule (bold in the following paragraph); no other aspects of the study are affected.

The radioimmunotherapy regimen included long-course radiotherapy (45–50.4 Gy in 25 fractions, administered five times per week for 5 weeks), followed by intravenous administration of the anti-PD-1 monoclonal antibody sintilimab **(200 mg intravenously guttae for three cycles at a 3-week interval**). **Three or four days** after completing radiotherapy, a second tumor biopsy was performed via colonoscopy. Surgery was scheduled approximately **nine weeks** after radiotherapy, during which additional tumor tissue and 5 mL peripheral blood samples were collected.

We sincerely apologize for any inconvenience caused by our proofreading oversight.

