# Supplementary material for: Longitudinal single-cell and TCR repertoire profiling characterizes clonal entrapment in patients with pMMR/MSS locally advanced rectal cancer
Source: Cell Discov. 2026 Jun 30;12:46. doi: 10.1038/s41421-026-00900-w (PMC13315945; doi:10.1038/s41421-026-00900-w)
Supplement: Supplementary file 1 — Supplementary Figures [file 41421_2026_900_MOESM1_ESM.pdf]

Supplemental Material

Supplementary Tables

Supplementary Table 1. Clinical characteristics of 83 patients with rectal cancer in the bulk RNA-seq cohort.
Supplementary Table 2. Clinical characteristics of 22 patients with rectal cancer in the scRNA-seq cohort.
Supplementary Table 3. Basic QC metrics for each scRNA-seq sample.
Supplementary Table 4. Bulk RNA-seq expression matrix.
Supplementary Table 5. Key resources.

Supplementary Figures and Legends

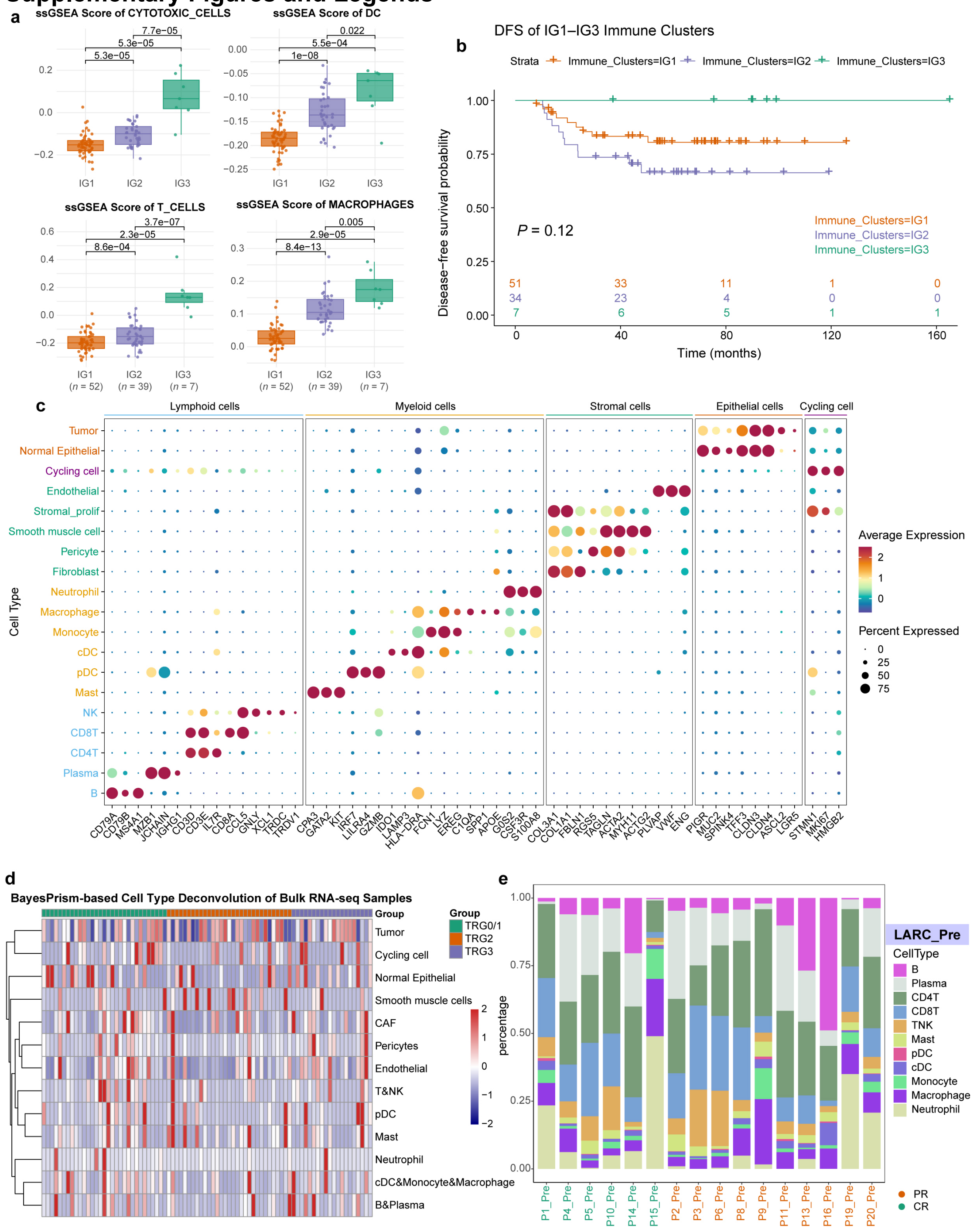

Figure S1

(**a**) Box plots showing immune cell scores derived from ssGSEA across the 3 immune groups in the GSE209746 public dataset. *P*-values were calculated using the Wilcoxon rank-sum test. (**b**) Survival curves showing disease-free survival (DFS) across the 3 immune groups in the GSE209746 public dataset. (**c**) Bubble plot showing the expression of marker genes for all major cell types, with each cell type highlighted in its corresponding category color. (**d**) Heatmap showing the deconvolution of in-house bulk RNA-seq results based on in-house LARC scRNA-seq data. (**e**) Bar plot showing the proportion of various immune cell types in each sample before treatment in CR and PR groups.

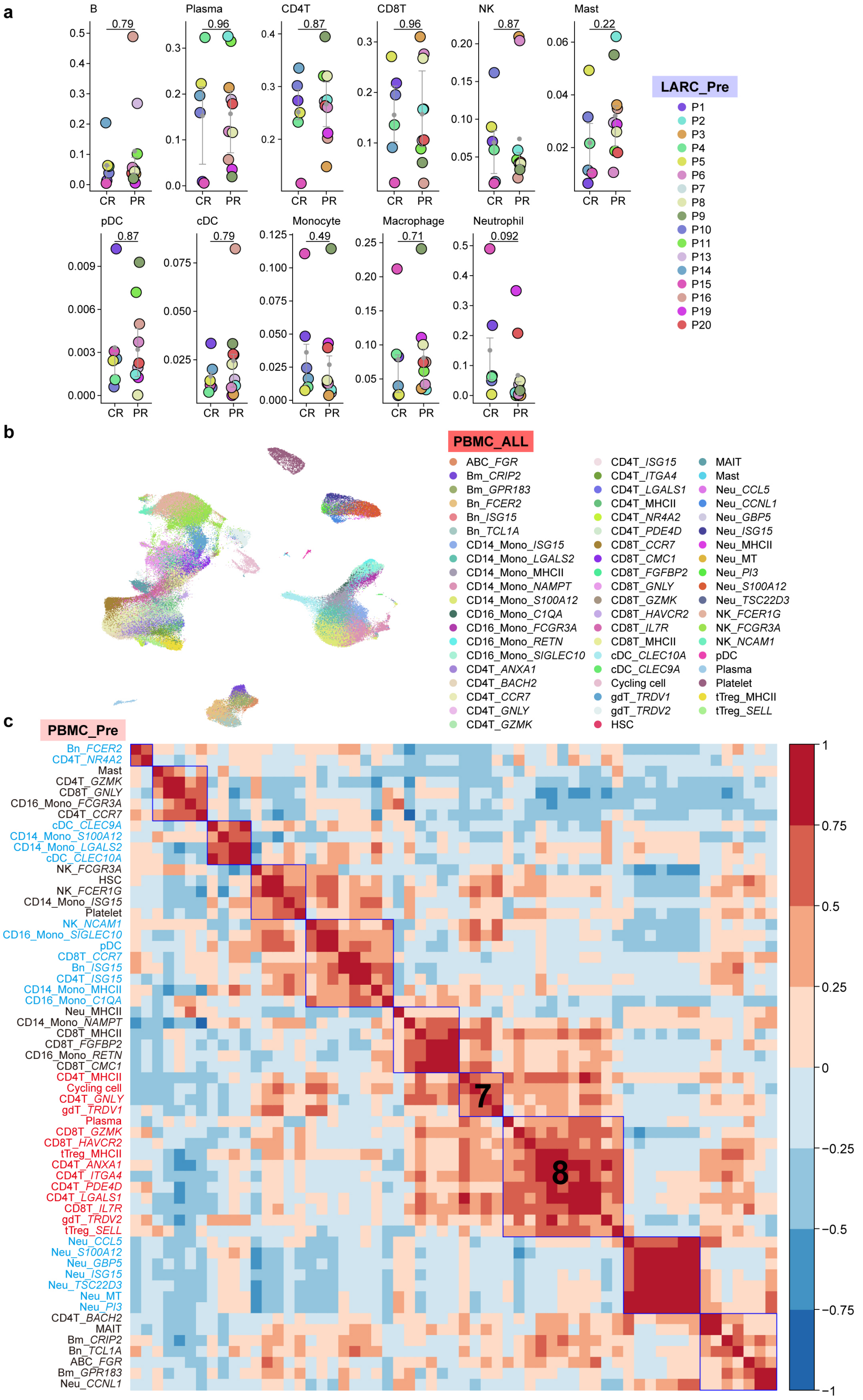

Figure S2

(**a**) Boxplots comparing the proportions of immune cell types between the CR and PR groups in LARC tumor samples at baseline. *P*-values were calculated using the Wilcoxon rank-sum test. (**b**) UMAP plot showing the distribution of 59 PBMC subtypes. (**c**) Heatmap showing the correlation of the proportion of various PBMC subtypes in each sample before treatment. Ten co-occurrence clusters are outlined in the plot, with cluster 7 and cluster 8 prominently highlighted.

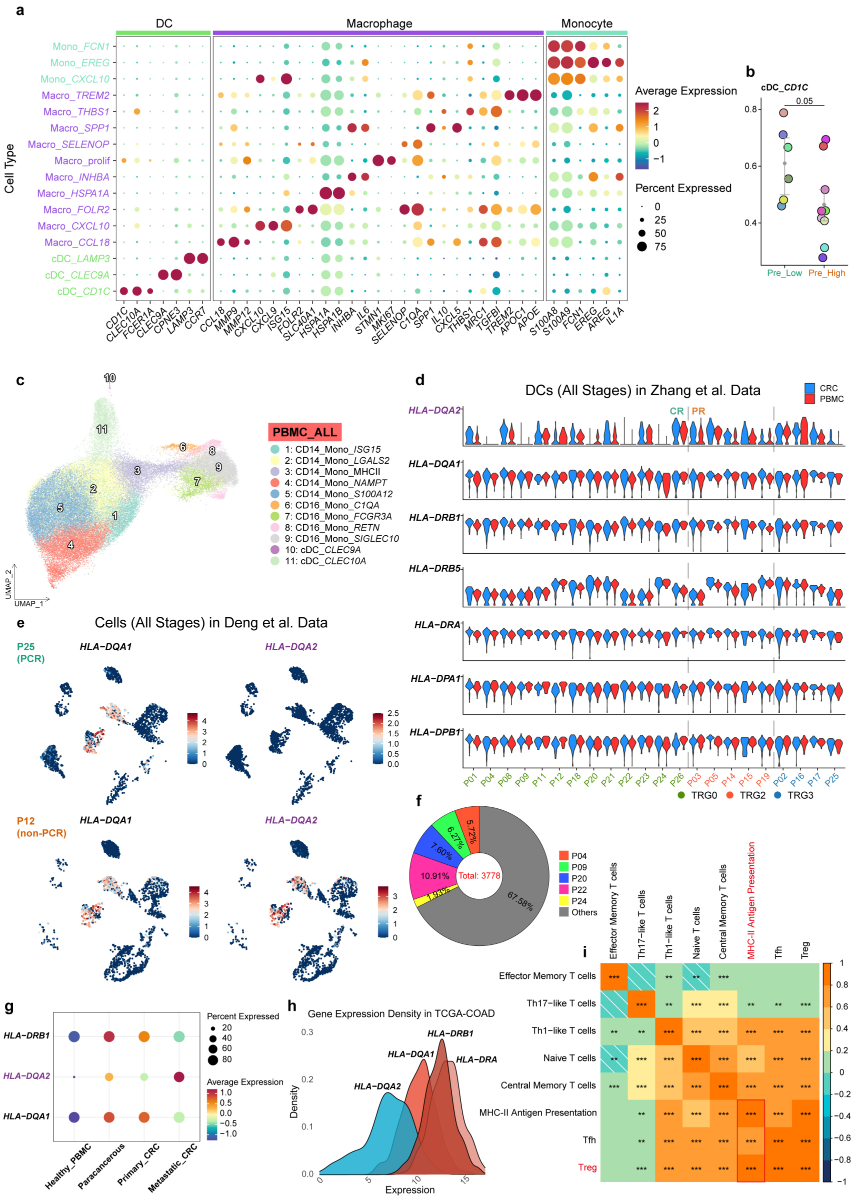

Figure S3

(**a**) Bubble plot showing the expression of marker genes for all myeloid cell subtypes, with each cell type highlighted in its corresponding category color. (**b**) Boxplot showing difference in the proportion of the cDC_*CD1C* subtype between low- and high-*HLA-DQA2* groups at baseline. (**c**) UMAP projection of macrophages, monocytes, and cDCs in all PBMC samples. (**d**) Violin plot showing the expression of various MHC-II molecules, including *HLA-DQA2*, in cDCs from CRC tissue (blue) and peripheral blood (red) at all treatment stages for each patient in the Zhang et al. dataset. CR patients are shown on the left, PR patients on the right. (**e**) UMAP plots showing the expression of *HLA-DQA1* and *HLA-DQA2* in all cells from CRC tissue of representative Pathological Complete Response (PCR) and non-PCR patients in the Deng et al. dataset. (**f**) Pie chart showing the number of cDCs contributed by patients with low *HLA-DQA2* expression in the Zhang et al dataset. (**g**) Bubble plot showing the expression of *HLA-DQA1*, *HLA-DQA2*, and *HLA-DRB1* in DCs from different tissue sources. (**h**) Ridge plots showing expression of MHC-II molecules in the TCGA-COAD dataset. (**i**) Heatmap showing correlations between MHC-II antigen presentation pathway scores and various CD4^{+} T cell subset scores in the CRC bulk RNA-seq public dataset. ***$P < 0.001$, by Spearman correlation analysis.

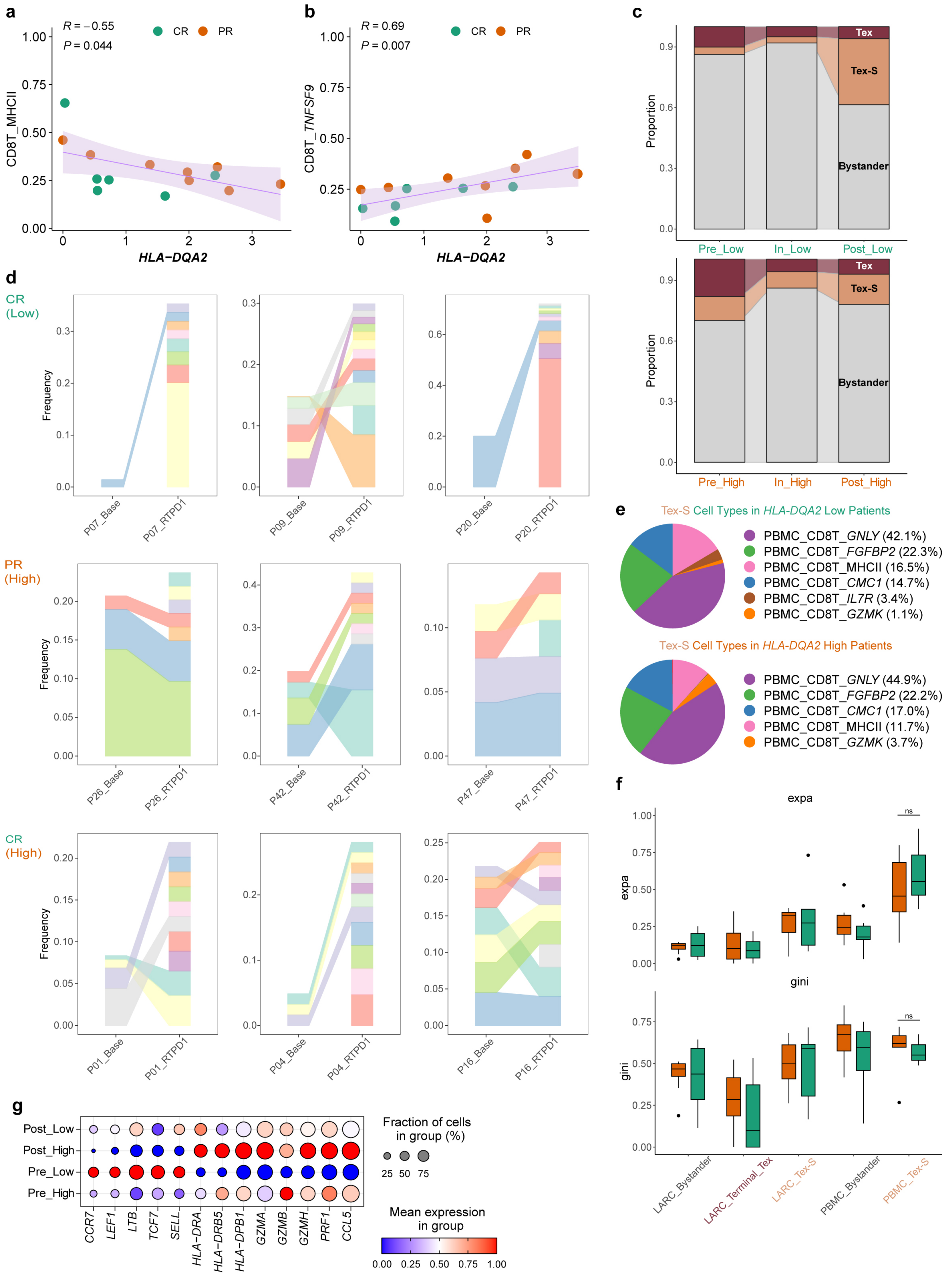

Figure S4

(**a**) Scatter plot showing the correlation between *HLA-DQA2* expression and the proportion of CD8T_MHCII cells among $CD8^+$ T cells after immunotherapy. (**b**) Scatter plot showing the correlation between *HLA-DQA2* expression and the proportion of CD8T_*TNFSF9* cells among $CD8^+$ T cells after immunotherapy. (**c**) Bar plot showing changes in the proportions of tumor-reactive $CD8^+$ T cells in LARC tissue across Pre, In, and Post groups with low and high *HLA-DQA2* expression. (**d**) Sankey diagrams showing the proportion of CDR3 amino acid sequences of the top ten $CD8^+$ T cell clones in TNBC after treatment for representative CR patients and non-CR patients, along with their pre-treatment frequency. (**e**) Pie charts showing the distribution of $CD8^+$ T cell subtypes within PBMC-derived Tex-S in low-*HLA-DQA2* (top) and high-*HLA-DQA2* (bottom) groups. (**f**) Boxplots comparing expansion (expa) and gini indices of tumor-reactive and bystander $CD8^+$ T cells from LARC tissues and PBMCs between low- and high-*HLA-DQA2* groups. (**g**) Bubble plot showing the expression of marker genes in peripheral blood $CD8^+$ T cells before and after treatment across two groups.

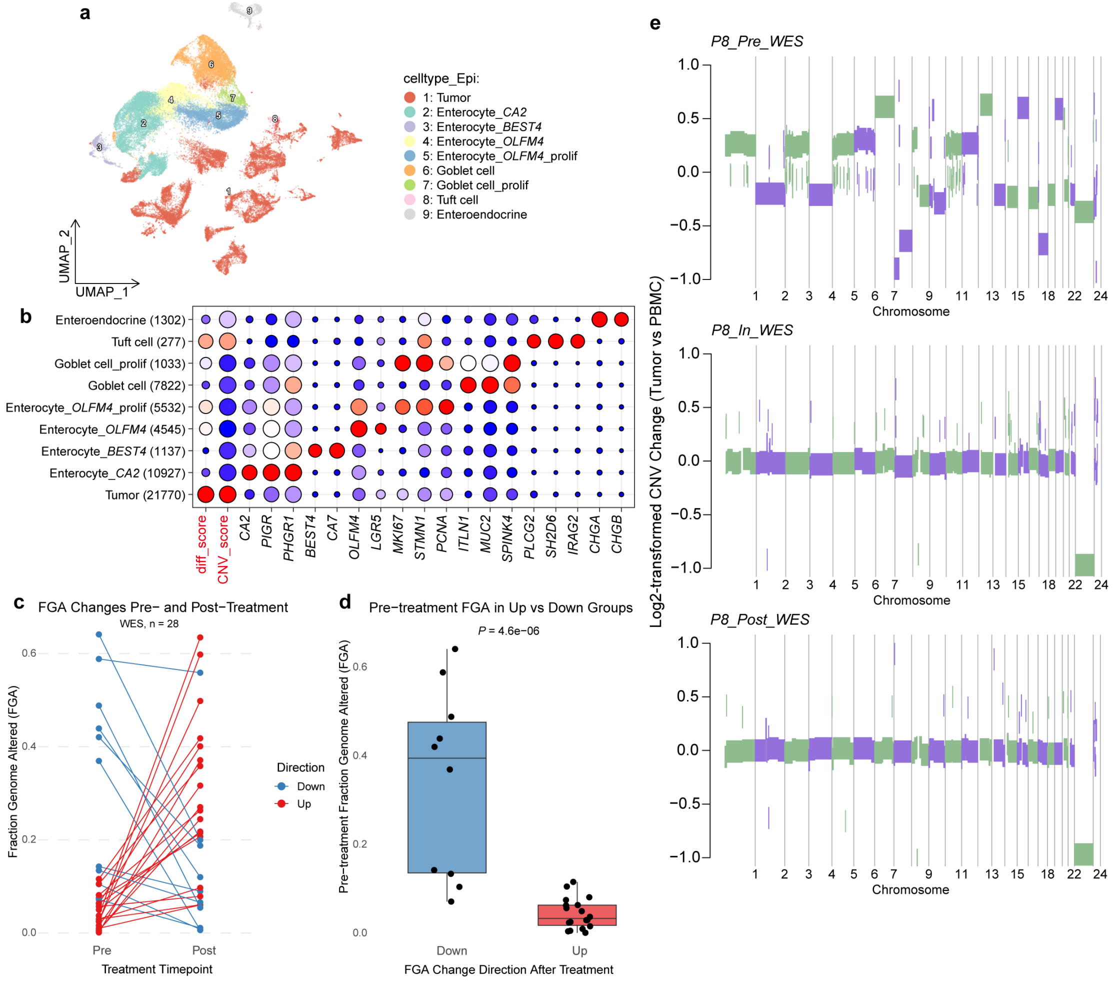

Figure S5

(**a**) UMAP projection of epithelial cells from all LARC samples. Each dot represents a single cell, colored according to its epithelial cell type. (**b**) Bubble plot showing the expression of marker genes for all epithelial cell types. (**c**) Scatter plot showing overall FGA changes in tumor tissue from the LARC WES cohort (n = 28) before and after chemoradiotherapy. Paired samples from the same patient are connected by lines, with blue indicating decreased FGA levels after treatment (Down). (**d**) Boxplot showing pre-treatment FGA levels in patients with decreased (Down) versus increased (Up) FGA after treatment. *P*-values were calculated using the Wilcoxon rank-sum test. (**e**) Log2-transformed CNV changes comparing tumor tissue with peripheral blood from patient P8 at different time points.

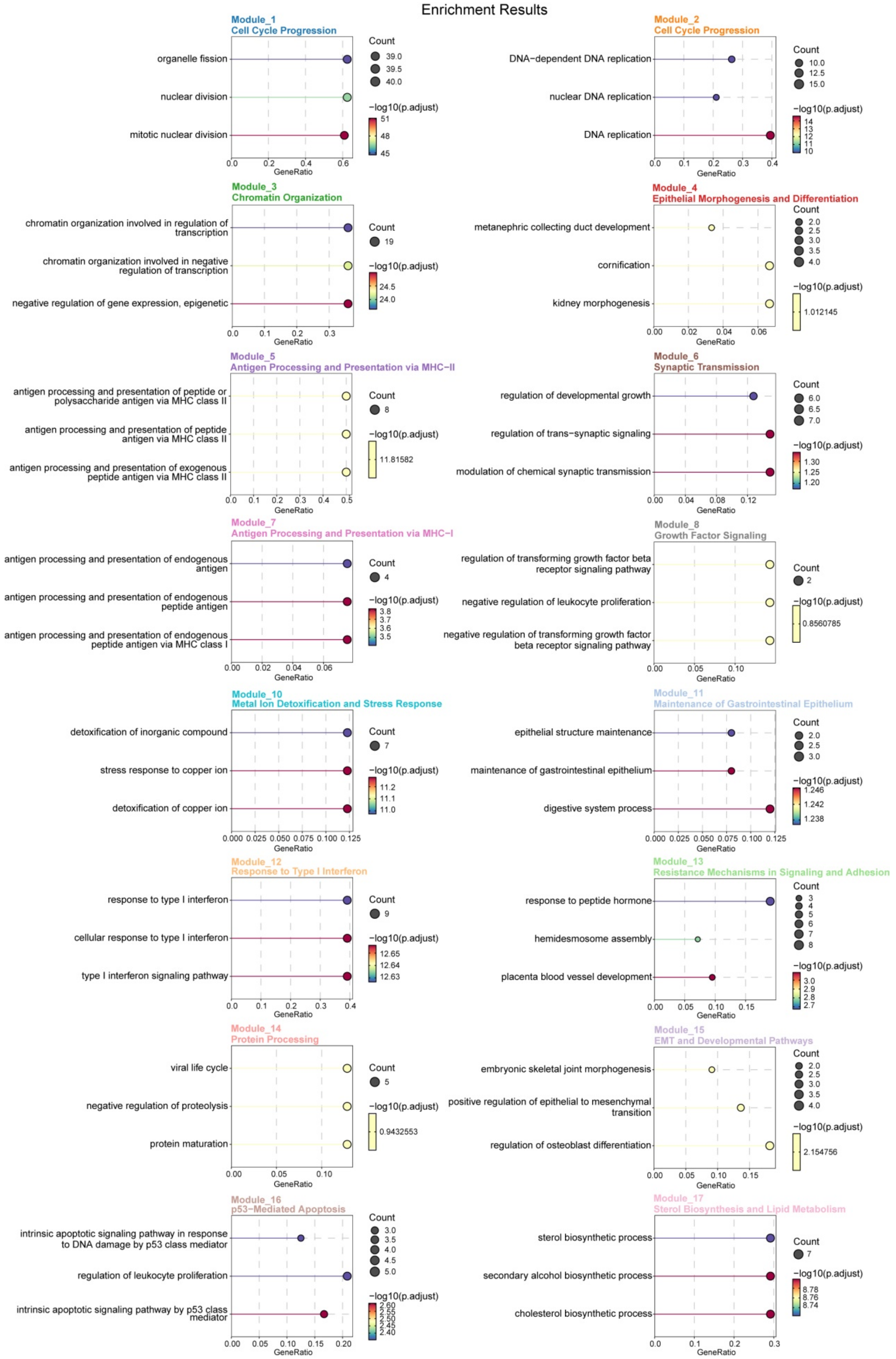

Figure S6 Pathway enrichment results of major genes in each module.

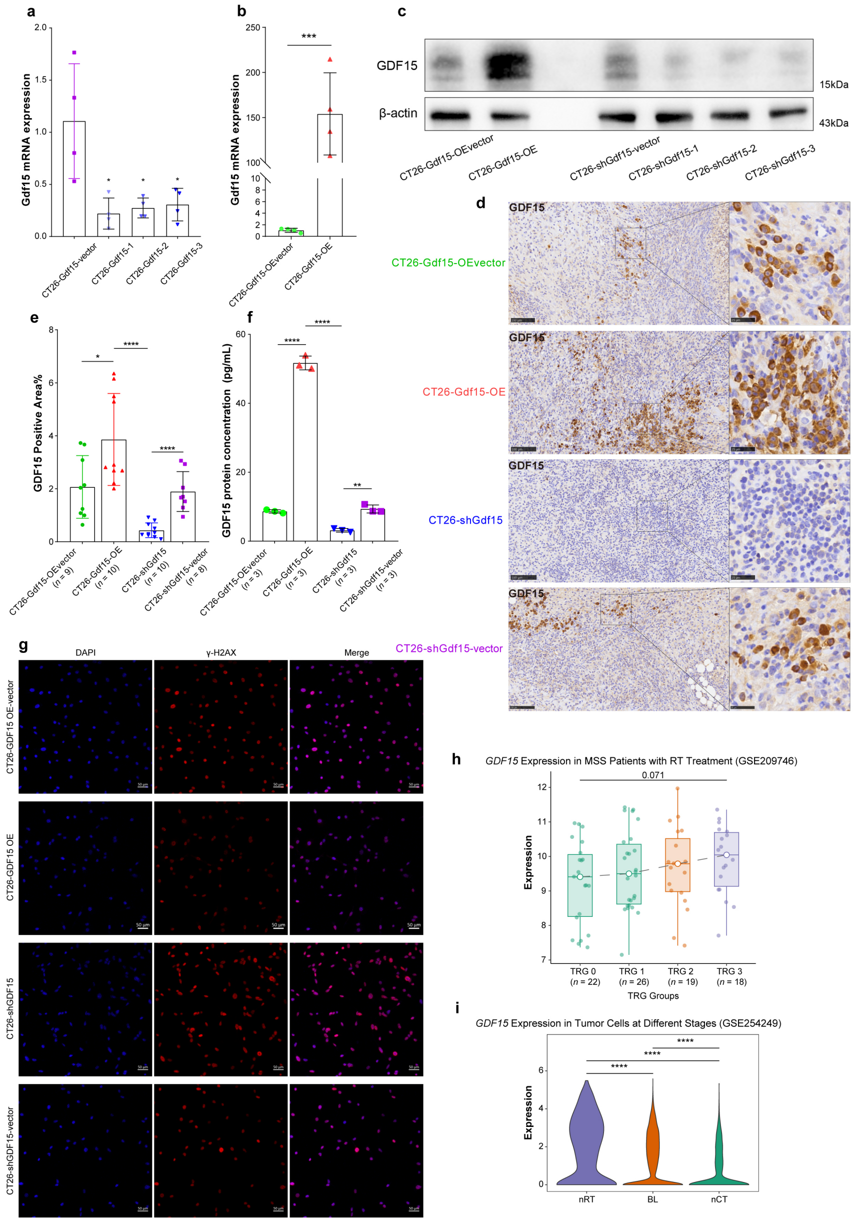

Figure S7

(**a**) Validation of GDF15 knockdown in CT26 cells by RT-PCR. **P* < 0.1, by two-tailed unpaired Student's *t*-test. (**b**) Validation of GDF15 stably overexpressing in CT26 cells by RT-PCR. ****P* < 0.001, by two-tailed unpaired Student's *t*-test. (**c**) Validation of GDF15 stably overexpressing and knockdown in CT26 cells by Western blotting. (**d**) GDF15 staining in mouse tumor tissues (or regions) after treatment from CT26-Gdf15-OE, CT26-shGdf15, and their respective controls. Scale bars, 100 μm (left) and 25 μm (right). (**e**) The average GDF15 positive Area% in each IHC staining was quantified using ImageJ. Data are shown as mean ± SD. ***P* < 0.01, *****P* < 0.0001, by Wilcoxon rank-sum test. (**f**) ELISA of CT26-conditioned media confirming distinct GDF15 levels. (**g**) Representative immunofluorescence images of the DNA damage marker γ-H2AX (red) in three cell lines 24h after exposure to 4 Gy radiation. Scale bars, 50μm. (**h**) Boxplots of baseline *GDF15* expression in MSS LARC patients from the bulk RNA-seq cohort, stratified by treatment response after radiotherapy. *P*-value was calculated using the two-tailed unpaired Student's *t*-test. (**i**) Violin plots of *GDF15* expression in tumor cells at different stages (nRT: post-radiotherapy, BL: baseline, nCT: post-chemotherapy) in the scRNA-seq cohort.

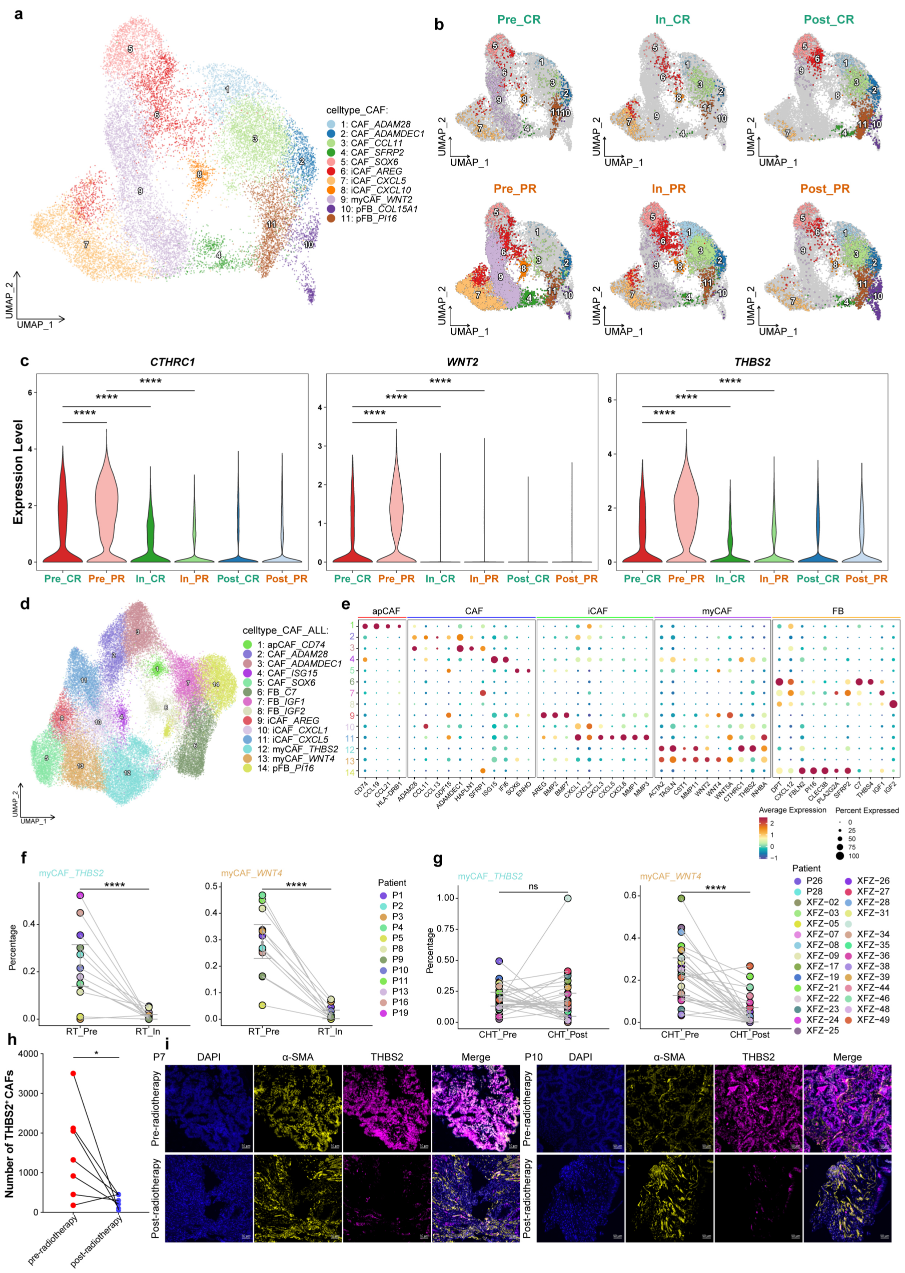

Figure S8

(**a**) UMAP projection of fibroblasts from all LARC samples. (**b**) UMAP plots showing the distribution of CAF subtypes across all groups. (**c**) Violin plots showing changes in *CTHRC1*, *WNT2*, and *THBS2* expression in the CR and PR groups before treatment, post-radiotherapy, and post-immunotherapy. (**d**) UMAP plot showing fibroblasts integrated from our dataset and the Lin et al. cohort, which received only chemotherapy. (**e**) Bubble plot showing the expression of marker genes across all CAF subtypes after integration. (**f**) Scatter plot showing changes in the proportions of myCAF_*THBS2* and myCAF_*WNT4* before and after radiotherapy in paired samples from this dataset. *P*-values were calculated using the Wilcoxon signed-rank test. (**g**) Scatter plot showing changes in the proportions of myCAF_THBS2 and myCAF_WNT4 before and after chemotherapy in paired samples from the Lin et al. dataset. ****$P < 0.0001$, by the Wilcoxon rank-sum test. (**h**) Number of THBS2^{+} CAFs in FFPE sections from seven patients before and after radiotherapy. Data are shown as mean ± SD. *$P < 0.1$, by the Wilcoxon rank-sum test. (**i**) Representative multiplex immunohistochemistry staining of THBS2^{+} CAFs in FFPE sections from patients P7 (left) and P10 (right), pre- and post-radiotherapy. Scale bars: 50 µm.

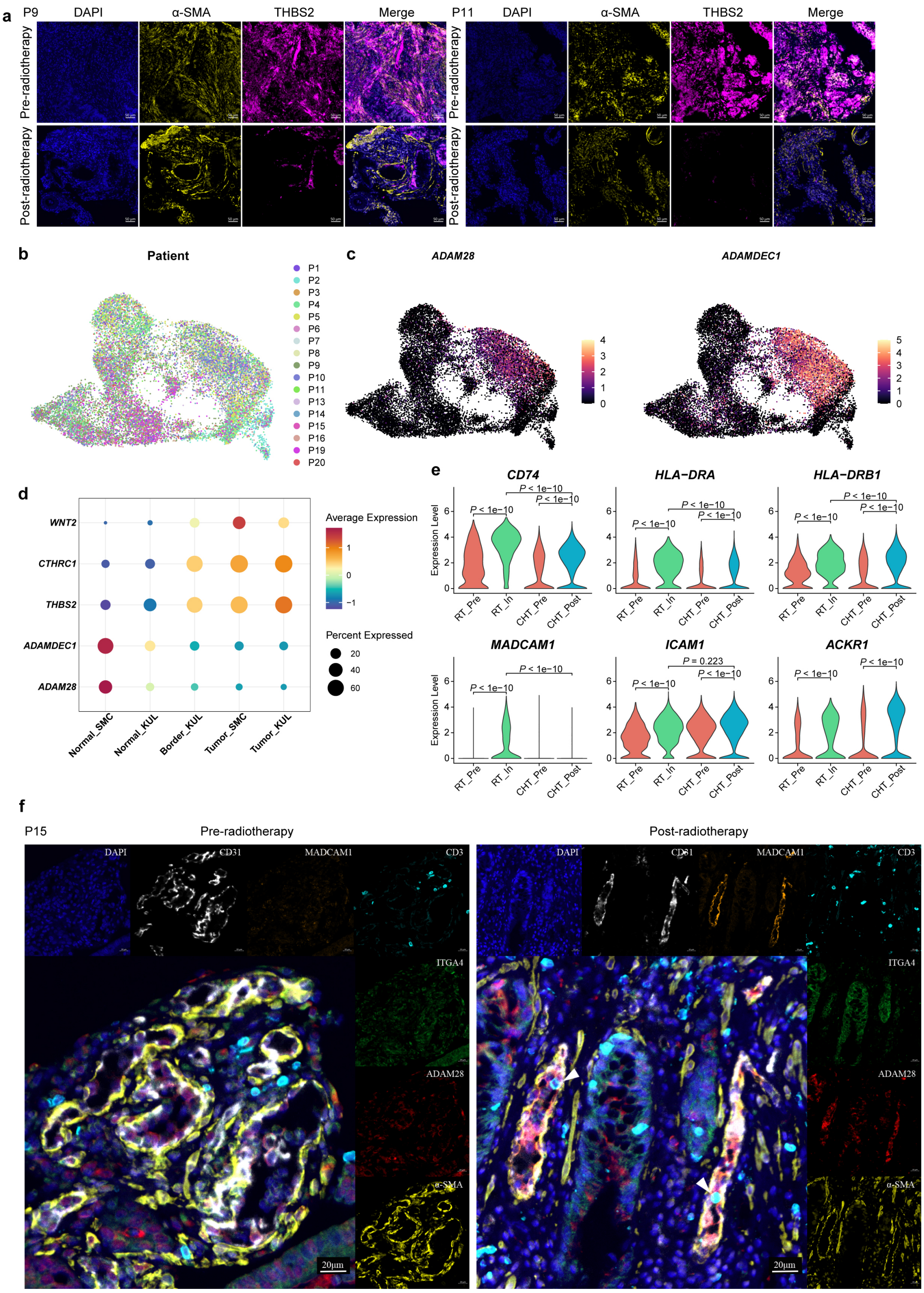

Figure S9

(**a**) Immunofluorescence staining shows the expression of α-SMA and THBS2 in pre-treatment and post-treatment samples. Scale bars, 50 μm. (**b**) The UMAP plot shows the patient origin of fibroblasts in all LARC samples. (**c**) The UMAP plot shows the expression of *ADAM28* and *ADAMDEC1* in all fibroblasts. (**d**) The bubble plot shows the expression of various marker genes in fibroblasts from the SMC and KUL public datasets. (**e**) Violin plots show the expression of various marker genes in endothelial cells before and after radiotherapy, and before and after chemotherapy, in the integrated dataset. *P*-values were obtained by Wilcoxon rank-sum test. (**f**) Immunofluorescence staining shows the expression of CD31, MADCAM1, CD3, ITGA4, ADAM28, and α-SMA before and after radiotherapy. Scale bars, 20 μm.
