# Supplementary material for: Longitudinal single-cell and TCR repertoire profiling characterizes clonal entrapment in patients with pMMR/MSS locally advanced rectal cancer
Source: Cell Discov. 2026 Jun 30;12:46. doi: 10.1038/s41421-026-00900-w (PMC13315945; doi:10.1038/s41421-026-00900-w)
Supplement: Supplementary file 3 — Clinical trial protocol [file 41421_2026_900_MOESM3_ESM.docx]

Neoadjuvant Long-course Chemoradiotherapy Followed by Immunotherapy for Locally Advanced Mid-low Rectal Cancer (NLCCRIT-LARC)

|  |  |
| --- | --- |
| Leading site of clinical trial: | Peking University People’s Hospital |
| Principal Investigator: | Prof. Zhanlong Shen |
| Version No.: | 1.0 |
| Version Date: | Jan 1, 2023 |

**Sponsor**: Peking University People’s Hospital

No.11 Xizhimen South Street, Xicheng District, Beijing

CONTENTS

[Protocol Summary 4](#_Toc213065683)

[1. Study background and scientific rationale 8](#_Toc213065684)

[1.1. Background information 8](#_Toc213065685)

[1.1.1. Overview of rectal cancer 8](#_Toc213065686)

[1.1.2. Treatment strategies for rectal cancer 8](#_Toc213065687)

[2. Study Endpoints 10](#_Toc213065688)

[2.1. Primary Endpoints 10](#_Toc213065689)

[2.2. Secondary Endpoints 10](#_Toc213065690)

[2.3. Exploratory Endpoints 11](#_Toc213065691)

[3. Research design 11](#_Toc213065692)

[3.1. Overall Design 11](#_Toc213065693)

[4. Subject selection and withdrawal 13](#_Toc213065694)

[4.1. Inclusion Criteria 13](#_Toc213065695)

[4.2. Exclusion criteria 14](#_Toc213065696)

[4.3. Subject withdrawal of study treatment 14](#_Toc213065697)

[5. Study medication 15](#_Toc213065698)

[5.1. Overview of investigational product 15](#_Toc213065699)

[5.1.1. Drug storage and stability 15](#_Toc213065700)

[5.1.2. Dosing Regimen 16](#_Toc213065701)

[5.1.3. Dose Adjustments 16](#_Toc213065702)

[5.2. Screening Period 17](#_Toc213065703)

[5.3. Treatment period and end of treatment 19](#_Toc213065704)

[5.3.1. Surgery records and pathology assessment 20](#_Toc213065705)

[5.4. Follow-up Period 21](#_Toc213065706)

[6. Evaluation 22](#_Toc213065707)

[6.1. Efficacy Evaluation 22](#_Toc213065708)

[6.2. Efficacy Evaluation Method 22](#_Toc213065709)

[6.2.1. Tumor evaluation based on RECIST v1.1 22](#_Toc213065710)

[6.2.2. Pathological efficacy evaluation 23](#_Toc213065711)

[6.3. Safety Evaluation 24](#_Toc213065712)

[6.3.1. Surgical safety 24](#_Toc213065713)

[6.3.2. Adverse Event 24](#_Toc213065714)

[7. Adverse Event Reporting 24](#_Toc213065715)

[7.1. Adverse events (AE) 24](#_Toc213065716)

[7.1.1. Definition of Adverse Events 24](#_Toc213065717)

[7.1.2. Criteria for determining the severity of adverse events 25](#_Toc213065718)

[7.1.3. Criteria for judging the relationship between adverse event and study drug 25](#_Toc213065719)

[7.2. Serious Adverse Event (SAE) 26](#_Toc213065720)

[7.2.1. Definition of Serious Adverse Event 26](#_Toc213065721)

[7.2.2. Hospitalisation 26](#_Toc213065722)

[7.2.3. Disease progression and death 27](#_Toc213065723)

[7.2.4. Reporting system of serious adverse events 28](#_Toc213065724)

[ADVERSE EVENT RECONCILIATION PROCESS 35](#_Toc213065725)

[8. Study Management 36](#_Toc213065726)

[8.1. Ethical guidelines and informed consent form 36](#_Toc213065727)

[8.2. Protocol amendment 37](#_Toc213065728)

[8.3. Quality assurance of clinical trial 38](#_Toc213065729)

[9. Statistical analysis of data 38](#_Toc213065730)

[9.1. Sample Size 38](#_Toc213065731)

[9.2. Analysis Population 38](#_Toc213065732)

[9.3. Statistical Methods 39](#_Toc213065733)

[10. Protection of subjects 39](#_Toc213065734)

[11. Clinical study progress 39](#_Toc213065735)

# Protocol Summary

| **Study Title** | Neoadjuvant Long-course Chemoradiotherapy Followed by Immunotherapy for Locally Advanced Mid-low Rectal Cancer (NLCCRIT-LARC) |
| --- | --- |
| **Protocol No.** | NCT06493240 |
| **Version No** | 1.0 |
| **Sponsor** | Peking University People’s Hospital |
| **Principal Investigator** | Prof. Zhanlong Shen |
| **Participating sites** |  |
| **Research design** | A single-arm, open-label, phase II trial |
| **Study objective** | **Primary Objective**   - pathological complete response (pCR) rate   **Secondary Objectives**   - Sphincter preserving rate - Immune-related adverse event rate - Treatment-related adverse event rate - Incidence rate of surgical complications   **Exploratory Objectives**   - Longitudinal single-cell RNA-seq (scRNA-seq) and single-cell TCR-seq (scTCR-seq) of tumor tissue and matched peripheral blood to identify immune programs of response vs resistance |
| **Research subjects** | Previously untreated and confirmed by biopsy as rectal adenocarcinoma and MRI shows no distant metastasis, stage II/III (except T4b) |
| **Treatment Regimen** | The treatment regimen is 45-50.4Gy/25 fractions + 5-FU long-course radiotherapy and chemotherapy (5 times per week for 5 weeks, total dose of 45 to 50.4Gy). On the 15th day after the end of radiotherapy and chemotherapy, tumor tissue was biopsied and PD-1 monoclonal antibody (Sintilimab, 200mg, once a week, for 3 weeks) treatment was performed. The surgery was scheduled 8-12 weeks after radiotherapy completion. |
| **Inclusion Criteria** | Patients must meet all of the following inclusion criteria to be enrolled in the study:   1. Age: 18-80 years old 2. Previously untreated and confirmed by biopsy as rectal adenocarcinoma 3. MRI shows no distant metastasis, stage II/III (except T4b) 4. The lower edge of the tumor must be within 10 cm of the anal margin 5. ECOG performance status 0-2 6. Willing and able to comply with the research protocol 7. Agree to use blood and tissue samples for research 8. No history of anti-tumor treatment (such as radiotherapy, chemotherapy, immunotherapy, biology, herbal medicine, etc.) 9. No immune system diseases (such as systemic lupus erythematosus, rheumatoid arthritis, systemic vasculitis, scleroderma, pemphigus, dermatomyositis, mixed connective tissue disease, autoimmune hemolytic anemia, hyperthyroidism/hypothyroidism, ulcerative colitis, autoimmune hemolytic anemia, HIV infection, etc.) 10. No significant functional impairment of major organs (such as heart, lungs, liver, kidneys, etc.) 11. No jaundice or gastrointestinal obstruction; No acute/chronic infection 12. No obvious abnormalities in blood routine and biochemical tests: neutrophils≥1.5×109/L, hemoglobin≥80g/L, platelets≥100×109/L, total bilirubin≤1.5×ULN, ALT, AST≤2.5×ULN 13. No social or mental disorders 14. For women of childbearing age, the serological pregnancy test result needs to be negative, and effective contraception measures need to be taken within 60 days |
| **Exclusion criteria** | Patients with any of the following are not to be enrolled in the study:   1. Multiple types of cancer, or accompanied by tumors other than rectal cancer 2. Having received any anti-cancer treatment (surgery, medication, etc.) within the past 5 years 3. Recent history of major surgery 4. Conditions affecting the absorption of capecitabine through the gastrointestinal tract (such as inability to swallow, nausea, vomiting, chronic diarrhea, etc.) 5. Having any type of uncontrolled, severe, accompanying disease, allergic to any component of the study; estimated survival period≤5 years due to any reason 6. Preparing or previously having undergone organ or bone marrow transplantation 7. Receiving immunosuppressive or systemic hormone therapy within 1 month before inclusion for immunosuppression 8. For patients with a history of central nervous system diseases, the investigator needs to judge whether the clinical severity hinders signing the informed consent or affects the patient's oral medication compliance 9. Other situations/problems that may affect the research results or lead to the termination of the study (such as alcohol abuse, drug abuse, etc.) 10. Pregnant or lactating women, or women planning to conceive during treatment |
| **Statistical considerations** | The primary endpoint is pathological complete response (pCR). The trial is designed as a single-arm Phase II study with 80% power to detect an increase in the pCR rate from 5% (historical control for pMMR/MSS LARC after standard long-course radiotherapy) to 20% with a one-sided alpha of 0.05. Accounting for an anticipated 10% drop-out rate, a total of 30 patients will be enrolled. |
| **Period during which study was conducted** | First patient enrolled: 2023-01-01.  Planned enrollment duration: approximately 24 months to accrue ~30 patients.  Planned follow-up after treatment: 5 years (for disease-free survival and overall survival assessments). |

# Study background and scientific rationale

## Background information

### Overview of rectal cancer

Colorectal cancer is one of the most common malignant tumors of the digestive tract worldwide. According to the 2020 Beijing Cancer Registry Annual Report, the incidence rate of colorectal cancer among Beijing's registered residents ranked second among men and fourth among women in 2017. Regardless of gender, the mortality rate of colorectal cancer ranked second among malignant tumors. Among them, rectal cancer accounts for approximately 50% of all colorectal cancers and is a key research object in clinical diagnosis and treatment. However, the early clinical manifestations of rectal cancer are not typical. Most patients seek medical attention due to symptoms such as anemia, changes in stool consistency, abdominal pain, and abdominal masses, at which point the tumor has often progressed to an advanced stage. Currently, radical surgery is the main treatment method for rectal cancer in clinical practice. However, for locally advanced patients, it is difficult to completely remove the lesion through surgery alone, and recurrence is common after surgery. Therefore, it is necessary to explore individualized comprehensive treatment strategies for locally advanced rectal cancer patients to improve their prognosis.

### Treatment strategies for rectal cancer

At present, clinical treatment guidelines for rectal cancer both domestically and internationally recommend preoperative radiotherapy and chemotherapy for patients with locally advanced middle and low rectal cancer (clinical stage ≥T3N+). Numerous studies have shown that the local recurrence rate of locally advanced rectal cancer significantly decreases after preoperative radiotherapy. In clinical research, Rodel et al classified the degree of pathological response after rectal cancer radiotherapy into three grades: complete pathological response, partial pathological response, and poor pathological response. The results showed that in patients with rectal cancer who received preoperative radiotherapy and radical surgical resection, the 5-year disease-free survival rate (DFS) was 86% for the complete pathological response group, 75% for the partial pathological response group, and 63% for the poor pathological response group (P = 0.006). Fokas et al also obtained similar results: in 386 patients who received rectal cancer radiotherapy and chemotherapy, the 10-year cumulative DFS of the complete pathological response group, partial pathological response group, and poor pathological response group were 89.5%, 73.6%, and 63% respectively, with statistical significance (P = 0.008). These studies indicate that the degree of tumor regression after preoperative radiotherapy for rectal cancer significantly affects the prognosis of patients. Therefore, for patients with rectal cancer, how to further improve the degree of pathological response after radiotherapy is a key issue for improving the prognosis of rectal cancer.

The research found that patients with higher infiltration density of CD4+ and CD8+ T lymphocytes in tumor tissues before radiotherapy for rectal cancer had a better response to radiotherapy for rectal cancer. Anitei et al further analyzed the enrichment score of T lymphocytes in tumor tissues of rectal cancer patients before radiotherapy, and the results showed that patients without a response to radiotherapy had a lower immune score, and the study found that the mutation load in tumor tissues before radiotherapy was positively correlated with the sensitivity to neoadjuvant therapy, and patients with higher mutation load had higher sensitivity to radiotherapy and chemoradiotherapy, while the characteristics of tumor mutation load and neoantigens could be indirectly reflected through the tumor immune microenvironment. The research team earlier also found through bulk sequencing analysis that the activation of related immune pathways was widely enriched in different radiotherapy-sensitive samples of rectal cancer.

The characteristics of the tumor microenvironment of rectal cancer patients may be correlated with the efficacy of radiotherapy, and in radiotherapy-resistant tumor tissues, abnormal tumor blood vessels, more myeloid-derived suppressive cells (MDSCs) and tumor-associated macrophages (TAMs) infiltration, the latter inhibit the proliferation of dendritic cells by secreting cytokines, leading to the downregulation of co-stimulation and activation of Tregs cells to suppress the enrichment of cytotoxic T lymphocytes. Therefore, for patients with rectal cancer undergoing radiotherapy, the target for relieving immune suppression may be a key entry point for further improving the efficacy of radiotherapy and improving prognosis.

A study reported at ASCO in 2019 showed that the pCR rate of MSS-type locally advanced rectal cancer patients treated with preoperative long-course concurrent radiotherapy combined with nivolumab (PD-1 inhibitor) reached 30%, and the pCR rate of MSI-H patients reached 60%. A study at the ASCO conference in 2021 reported that 27 cases of rectal cancer received preoperative radiochemotherapy combined with immunotherapy, and the overall pCR rate was as high as 48%, the downstaging rate reached 70%, and the rate of preservation of anus reached 89%. These studies suggest that preoperative radiotherapy combined with immune checkpoint inhibitors can effectively improve the efficacy of rectal cancer radiotherapy and has important clinical application prospects. However, it is still unclear which rectal cancer patients can benefit from radiotherapy combined with immune checkpoint inhibitor treatment, and the biomarkers for treatment benefit and new targets have not yet been clarified, and further research is needed.

# Study Endpoints

## Primary Endpoints

Pathologic complete response rate (pCR) based on TRG scoring criteria: refers to the percentage of subjects with no viable tumor cells (including lymph nodes, stage ypT0N0M0) seen under light microscopy.

## Secondary Endpoints

- Sphincter preserving rate：proportion of patients with preserved anal sphincter

Time Frame: instantly after surgery;

- Immune-related adverse event rate: adverse event rate that is deemed to be associated with PD-1 inhibition

Time Frame: from commencing of PD-1 inhibition to the 30th day after surgery;

- Treatment-related adverse event rate: adverse event rate that is deemed to be associated with all treatments

Time Frame: from commencing of treatment to the 30th day after surgery;

- Incidence rate of surgical complications: incidence rate of surgical complications within 30 days after surgery

Time Frame: within 30 days after surgery;

Exploratory Endpoints

- Longitudinal single-cell RNA-seq (scRNA-seq) and single-cell TCR-seq (scTCR-seq) of tumor tissue and matched peripheral blood to identify immune programs of response vs resistance

# Research design

## Overall Design

This study is a single-arm, open-label, phase II trial to observe and evaluate the efficacy and safety of capecitabine-based long-term radiotherapy followed by 3 cycles Sintilimab (PD-1 inhibitor) for locally advanced rectal cancer.

The study aims at the treatment-naïve patients with gastric or gastroesophageal junction adenocarcinoma at the staging of cT3-4 or N+, M0 (staging according to AJCC version 8).

With pathological complete response rate (pCR) as the primary study endpoint, 46 patients with resectable locally advanced gastric adenocarcinoma and gastroesophageal junction adenocarcinoma who have not received treatment are planned to be enrolled.

Subjects are enrolled after signing informed consent and passing screening.

Subjects will receive the following treatments:

45-50.4Gy/25 fractions + 5-FU long-course radiotherapy and chemotherapy (5 times per week for 5 weeks, total dose of 45 to 50.4Gy) before surgery →PD-1 monoclonal antibody (Sintilimab, 200mg, once a week, for 3 weeks) →radical surgery

The surgery was scheduled 8-12 weeks after radiotherapy completion.

Radiographic assessment:

All lesions are recorded and assessed according to RECIST 1.1. Imaging examinations include chest CT, enhanced CT scans of the abdomen and pelvis, and chest CT plain scan + abdominal and pelvic MRI scans could be performed if allergic to enhanced CT contrast agents; brain enhanced MRI or enhanced CT is also required to rule out brain metastases in patients with suspected brain metastases. Bone scan should be performed when bone metastasis is confirmed or clinically suspected. Colonoscopy examinations should be conducted before the start of radiotherapy, after the completion of radiotherapy, and after immunotherapy to evaluate the therapeutic effects.

Within 1 weeks after the radiotherapy; within 1 weeks after the frist dose of PD-1 monoclonal antibody in Cycle 3; at the end of treatment; and imaging assessments at least every 3 months in subsequent Year 1 and at least every 6 months from Year 2 until radiographic progression or recurrence, receipt of other antineoplastic therapy, withdrawal of informed consent, loss to follow-up, or death.

If treatment is discontinued during the treatment period for reasons other than radiographic progression, imaging should be performed at the end of treatment unless imaging has already been performed within 28 days.

Pathological assessment: According to the operation guidelines specified in the protocol, pathological efficacy, degree of eradication, ypT staging and ypN staging are evaluated.

Subjects will be followed for survival every 3 months in the first year and every 6 months from the second year after the end of treatment, and their survival status and subsequent anti-tumor treatment will be collected and recorded until death or loss to follow-up.

# Subject selection and withdrawal

Subjects must meet the following criteria to be allowed to participate in this study. All medical or non-medical conditions of each subject are taken into account for their compliance or non-compliance with the trial criteria.

Before a subject is enrolled in the study, the investigator should review, confirm and document whether the subject is suitable for the study.

## Inclusion Criteria

Patients must meet all of the following inclusion criteria to be enrolled in the study:

1. Patients voluntarily participate in the study and sign the informed consent form;
2. Age ≥ 18 years and ≤ 80 years;
3. Previously untreated and confirmed by biopsy as rectal adenocarcinoma;
4. MRI shows no distant metastasis, stage II/III (except T4b)
5. The lower edge of the tumor must be within 10 cm of the anal margin;
6. Willing and able to comply with the research protocol;
7. Agree to use blood and tissue samples for research;
8. No history of anti-tumor treatment (such as radiotherapy, chemotherapy, immunotherapy, biology, herbal medicine, etc.);
9. ECOG score 0-2;
10. No immune system diseases (such as systemic lupus erythematosus, rheumatoid arthritis, systemic vasculitis, scleroderma, pemphigus, dermatomyositis, mixed connective tissue disease, autoimmune hemolytic anemia, hyperthyroidism/hypothyroidism, ulcerative colitis, autoimmune hemolytic anemia, HIV infection, etc.);
11. No significant functional impairment of major organs (such as heart, lungs, liver, kidneys, etc.);
12. No jaundice or gastrointestinal obstruction; No acute/chronic infection;
13. No obvious abnormalities in blood routine and biochemical tests: neutrophils≥1.5×109/L, hemoglobin≥80g/L, platelets≥100×109/L, total bilirubin≤1.5×ULN, ALT, AST≤2.5×ULN;
14. No social or mental disorders;
15. For women of childbearing age, the serological pregnancy test result needs to be negative, and effective contraception measures need to be taken within 60 days;

## Exclusion criteria

Patients with any of the following are not to be enrolled in the study:

1. Patients with multiple types of cancer, or accompanied by tumors other than rectal cancer;
2. Having received any anti-cancer treatment (surgery, medication, etc.) within the past 5 years;
3. Recent history of major surgery;
4. Conditions affecting the absorption of capecitabine through the gastrointestinal tract (such as inability to swallow, nausea, vomiting, chronic diarrhea, etc.);
5. Having any type of uncontrolled, severe, accompanying disease, allergic to any component of the study; estimated survival period ≤ 5 years due to any reason;
6. Preparing or previously having undergone organ or bone marrow transplantation;
7. Receiving immunosuppressive or systemic hormone therapy within 1 month before inclusion for immunosuppression;
8. For patients with a history of central nervous system diseases, the investigator needs to judge whether the clinical severity hinders signing the informed consent or affects the patient's oral medication compliance;
9. Other situations/problems that may affect the research results or lead to the termination of the study (such as alcohol abuse, drug abuse, etc.);
10. Pregnant or lactating women, or women planning to conceive during treatment;

## Subject withdrawal of study treatment

Subjects can withdraw from the trial at any time on their own, or are withdrawn by the investigator due to safety or behavioral reasons, or because they are unable to comply with protocol-required study visit times or procedures at their site.

Reasons for withdrawal of a subject from the study may include:

1. Patients for whom combined organ resection is necessary during the operation;
2. Patients with distant metastasis confirmed during the operation, including liver, pelvic cavity, ovaries, peritoneum, and distant lymph node metastasis;
3. Patients who require emergency surgical resection due to intestinal obstruction, intestinal perforation, intestinal bleeding, etc;
4. Patients who request to withdraw from the study cohort for various reasons after being included in the study;
5. Patients with other non-tumor conditions that prevent them from continuing to receive this treatment plan;
6. Patients who cannot complete the study plan for various reasons after being included in the study;

# Study medication

## Overview of investigational product

In the study, study drugs are defined as Sintilimab.

|  | Sintilimab |
| --- | --- |
| Manufacturer | Innovent, China |
| Dose Form | Injection fluid |
| Strength | 100 mg |
| Route of Administration | Intravenous |

### Drug storage and stability

Sintilimab Injection: must be stored at 2°C to 8°C (36°F to 46°F) and protected from light, shock and freezing. The unopened vials can be stored at room temperature (up to 25°C, 77°F) and room light for up to 48 hours. The administration of Sintilimab infusion must be completed within 24 hours of preparation. If not used immediately, the infusion solution may be stored under refrigeration conditions (2°C to 8°C, 36°F to 46°F) for up to 24 hours, and a maximum of 8 hours of the total 24 hours can be at room temperature (up to 25°C, 77°F) and room light. The maximum of 8 hours under room temperature and room light conditions includes the product administration period.

The investigator, or his/her authorized representative (eg, pharmacist), will ensure that all study drug is stored in a secure area with controlled access that meets storage conditions and is stored in compliance with applicable regulatory requirements.

### Dosing Regimen

Sintilimab monotherapy after radiotherapy (3 cycles)

**The specific dose and mode of administration are as follows:**

Sintilimab: 200 mg, every 3 weeks, with 21 days as a cycle. This product is for intravenous injection only. Intravenous infusion over 30 minutes. The infusion tube used in the infusion must be equipped with a sterile, pyrogen-free, low-protein-binding infusion tube filter (pore size 0.2-1.2 μm). This product should not be administered by intravenous bolus or single rapid intravenous injection. This product can be directly infused with 10mg/ml solution, or diluted with sodium chloride solution for injection (9mg/ml, 0.9%) or glucose solution for injection (50mg/ml, 5%), the concentration can be as low as 1mg/ml.

The study drug is administered until the protocol-specified treatment termination criteria occur.

Imaging assessment is performed for the subjects within 1 weeks of the last dose of Sintilimab therapy; surgery is performed for operable subjects 2 weeks after the last dose.

### Dose Adjustments

Every effort should be made to give each subject access to the study drug according to the protocol. If study treatment interruption and/or dose adjustment are required due to adverse drug reactions, toxicity should be classified according to National Cancer Institute (NCI) Common Toxicity Criteria (CTC) Version 5.0, and the reasons for interruption/adjustment, actions taken and results should be recorded.

General principles for dose modification:

- Dose delays/modifications due to the toxicity of chemotherapy agents or Sintilimab may not affect the planned use of other study agents.
- The investigator may delay the full combination treatment (up to 14 days) to maintain synchronization of study treatments, as appropriate.
- Sintilimab should be administered no less than 12 days apart;

#### Dose modifications for nivolumab

For serious adverse reactions with Sintilimab, dosing may be interrupted or permanently discontinued based on the safety and tolerability of individual patient. Dose escalation or reduction is not recommended.

Immune-related adverse reactions: See the study procedures in Guidelines for the Management of Toxicity Associated with CSCO Immune Checkpoint Inhibitors (2021.V1) for specific management principles

## Screening Period

Tumor assessment, including tumor imaging assessment (CT/MRI/PET), endoscopy and pathological assessment, should be performed within 21 days of enrollment. Imaging examinations include chest CT, enhanced CT scan of abdomen and pelvis (CT scan slice thickness ≤ 5 mm). If allergic to enhanced CT contrast agent, chest CT plain scan + abdominal and pelvic MRI scan can be performed; if brain metastasis is suspected, brain enhanced MRI or enhanced CT should also be performed to rule out brain metastasis. Bone scan should be performed when bone metastasis is clinically suspected.

The following information will be collected within 14 days of enrollment:

- Blood routine: WBC, neutrophils, lymphocytes, RBC, hemoglobin and platelets;
- Blood biochemistry: including total bilirubin, direct bilirubin, ALT, AST, AKP, γ-GT, total protein, albumin, urea nitrogen/urea nitrogen, creatinine, endogenous creatinine clearance rate, uric acid, fasting blood glucose, triglyceride, cholesterol, potassium, sodium, chloride, calcium and phosphorus;
- Serum amylase: lipase test should be performed in case of abnormal serum amylase with clinical significance;
- Urinalysis: including urine protein, urine glucose, urine occult blood, urine red blood cells, white blood cells. If 2 consecutive semiquantitative assays show protein 2+, 24-hour urinary protein quantification test is performed, and if semiquantitative assay shows protein > 2+, 24-hour urinary protein quantification test is performed;
- Stool occult blood test: If stool occult blood is positive, reexamination should be performed. After reexamination, stool occult blood is still positive. Combined with clinical judgment, gastroscopy should be performed when necessary
- Coagulation function: including INR, APTT, PT and FIB;
- For subjects of childbearing potential, serum human chorionic gonadotropin (HCG) test is performed within 72 hours before enrollment;
- Cardiac enzymes: creatine kinase MB isoenzyme (CK-MB), troponin I (cTnI), lactate dehydrogenase;
- Vital signs (performed after sitting quietly for 5 minutes): including body temperature, blood pressure, pulse and respiratory rate;
- A complete physical examination is performed: general condition, head and face, skin, lymph nodes, eyes, ears, nose and throat, oral cavity, respiratory system, cardiovascular system, abdomen, genito-urinary system, musculoskeletal system, nervous system, and mental status;
- Height and weight measurement;
- ECOG scoring;
- 12-lead ECG (performed after sitting quietly for at least 5 minutes): heart rate, PR interval and QTc interval;
- Echocardiogram, including LVEF assessment;
- Blood pressure monitoring;
- Collection of concomitant medications/treatments;
- Collection of AEs;
- Verification of inclusion and exclusion criteria.
- Subjects may be enrolled if all inclusion criteria are met and none of the exclusion criteria are met.

## Treatment period and end of treatment

Study medication is detailed in 5.1.2 Regimen.

Relevant physical examinations and laboratory tests are performed during treatment to collect AEs and concomitant medication information.

During the treatment period, imaging examinations should be performed under the same conditions as the baseline examination (slice thickness of the scan, use of contrast agent, etc.), within 1 weeks after radiotherapy; within 1 weeks after the frist dose of PD-1 monoclonal antibody in Cycle 3; imaging assessment should be performed at the end of treatment; if treatment is discontinued due to reasons other than radiographic progression during the treatment period, imaging examination should be performed at the end of treatment, unless imaging examination has been performed within 28 days.

The following tests should be completed before administration on Day 1 of each treatment cycle, at the preoperative assessment and at the end of treatment. If the scheduled visit tests before the first dose have been performed during the screening period and within 3 days before the first dose, they may not be repeated before administration in Cycle 1:

- Blood routine: WBC, neutrophils, lymphocytes, RBC, hemoglobin and platelets;
- Blood biochemistry: including total bilirubin, direct bilirubin, ALT, AST, AKP, γ-GT, total protein, albumin, urea nitrogen/urea nitrogen, creatinine, endogenous creatinine clearance rate, uric acid, fasting blood glucose, triglyceride, cholesterol, potassium, sodium, chloride, calcium and phosphorus;
- Urinalysis: including urine protein, urine glucose, urine occult blood, urine red blood cells, white blood cells. If 2 consecutive semiquantitative assays show protein 2+, 24-hour urinary protein quantification test is performed, and if semiquantitative assay shows protein > 2+, 24-hour urinary protein quantification test is performed;
- Blood occult blood test: performed if necessary;
- Coagulation function: including INR, APTT, PT and FIB;
- For subjects of childbearing potential, pregnancy tests are performed as necessary during the treatment period.
- Cardiac enzymes: performed as necessary, including creatine kinase MB isoenzyme (CK-MB), troponin I (cTnI), lactate dehydrogenase;
- Vital signs (performed after sitting quietly for 5 minutes): including body temperature, blood pressure, pulse and respiratory rate;
- Physical examination: Targeted physical examination is performed as clinically indicated;
- Height and weight measurement;
- ECOG score;
- 12-lead ECG (performed after sitting quietly for at least 5 minutes): performed as necessary, including heart rate, PR interval and QTc interval;
- Echocardiogram: performed as necessary, including assessment of LVEF;
- Blood pressure monitoring

Subjects receiving nivolumab will undergo the following tests before dosing on Day 1 of each cycle and at the end of treatment in addition to the above tests:

- Serum amylase: lipase test should be performed in case of abnormal serum amylase with clinical significance;
- Thyroid function test (C1D1 not required);

### Surgery records and pathology assessment

The surgical specimens are preserved and processed according to the operation guidelines for pathological evaluation, and the pathological efficacy is assessed according to the TRG scoring criteria; the degree of radical cure and ypN staging are assessed according to the AJCC Version 8; at least the following information needed to be recorded:

- Length of operation
- Surgical method
- Tumor size
- Tumor site and the distance from the lower edge to the anus
- Number of lymph nodes detected
- Number of lymph node metastases
- Degree of eradication (RX/R0/R1/R2)
- Histological Type
- Microsatellite status (MSS/MSI-L/MSI-H)
- YpTMN staging (AJCC Version 8)
- Residual percentage of viable tumor cells in primary tumor (%)
- Pathologic response grading (TRG Criteria)
- Length of procedure is recorded
- Discharge time after surgery is recorded

## Follow-up Period

Following the end of treatment visit, the follow-up period is entered, which includes safety follow-up, tumor progression/recurrence, and survival follow-up.

Concomitant medications and concomitant therapies used for new or unresolved AEs related to trial treatment should be recorded after the end of treatment, up to 30 days after the last dose.

Adverse events should also be recorded until 30 days after the last dose after the end of treatment and followed up until resolution or stabilization of the adverse event.

Safety follow-up:

At 30 days (± 7 days) after the last dose of study drug, whether or not the subject starts a new anticancer therapy, the subject is required to return to the study site for safety follow-up or by telephone.

Follow-up of time to progression/relapse:

For subjects who do not have radiographic progression or recurrence at the end of treatment, radiographic assessments are performed at least every 3 months in Year 1 and at least every 6 months starting at Year 2 until radiographic progression or recurrence, receipt of other antineoplastic therapy, withdrawal of consent, loss to follow-up, or death.

Survival follow-up:

After the end of treatment, subjects will be followed for survival every 6 months until death, loss to follow-up, or the end of the study, whichever occurs first.

# Evaluation

## Efficacy Evaluation

Efficacy endpoints include the following:

- Pathological complete response (pCR) rate based on TRG scoring criteria: refers to the percentage of subjects with TRG grade 0 who have no viable tumor cells remaining in the primary tumor;
- Major pathological response rate (mPR) based on TRG scoring criteria: refers to the percentage of subjects with no vialbe tumor cells (including lymph nodes, stage ypT0N0M0) and single cell or small clusters of tumor cells scattered through fibrosis seen under light microscope;
- Post-neoadjuvant lymph node status (stage ypN) assessed based on AJCC version 8;
- R0 resection rate assessed based on AJCC version 8;
- Disease-free survival (DFS): refers to the time from the start of the postoperative baseline radiographic assessment to the time of disease recurrence or death, whichever occurs first, in subjects who are disease-free following surgery.
- OS: time from the date of enrollment to death due to any cause.

## Efficacy Evaluation Method

### Tumor evaluation based on RECIST v1.1

In this study, RECIST v1.1 is used to evaluate the tumor response of subjects.

Selection criteria of target lesions: At the premise of no more than 2 target lesions per organ and no more than 5 total lesions, more organs with tumor involvement should be covered as far as possible. The number of target lesions, site description, long diameter of each target lesion (except lymph nodes), short diameter of lymph node lesions, and the sum of diameters of all lesions are recorded at baseline.

In particular, it should be noted that if gastric lesions need to be selected as target lesions, imaging should be performed at baseline and follow-up when the degree of gastric distension is the same.

Baseline and post-treatment efficacy assessments should be performed using the same method and by the same investigator whenever possible.

### Pathological efficacy evaluation

The surgical specimens are preserved and processed, and the pathological efficacy is assessed according to the TRG scoring criteria; the degree of eradication and ypN staging were assessed according to the AJCC Version 8;

Pathological response evaluation: Based on surgically resected primary lesions, the sites of assessment should include all primary lesions seen by imaging before neoadjuvant therapy, and sections with possible residual tumor cells should be selected for assessment. Viable tumor cells are defined as cells with the ability to proliferate and are graded as follows:

Pathological response evaluation criteria (TRG scoring criteria)*

| Grade 0 (Complete regression) | No viable tumor cells seen under light microscope (including lymph nodes, staging ypT0N0M0) |
| --- | --- |
| Grade 1 (well regression) | Single cell or small clusters of tumor cells scattered through fibrosis |
| Grade 2 (partial regression) | Fibrosis predominates, outgrowing residual tumor |
| Grade 3 (no regression) | Extensive residual tumor, no or little tumor cell necrosis |
| *Remarks: 1) TGR score is limited to the primary tumor bed;  2) Tumor cells refer to viable tumor cells, excluding degenerative and necrotic cells;  3) Large acellular mucinous lakes may appear after radiotherapy/chemotherapy and cannot be considered as residual tumor. | |

Major pathological response is defined as a grade greater than or equal to 1, i.e., grade 0, 1.

## Safety Evaluation

### Surgical safety

Including 30-day operative mortality, operative complication rate (surgical site infection, anastomotic leakage, gastrointestinal bleeding, incision dehiscence, adhesive intestinal obstruction, biliary fistula and chylous fistula, fever of unknown origin with body temperature ≥ 37.5℃, etc.), reoperation rate, hospital stay, operation time.

### Adverse Event

The evaluation of AEs includes type, incidence, severity (graded according to NCI-CTCAE v5.0), onset and end time, whether it is an SAE and the correlation and outcome.

# Adverse Event Reporting

## Adverse events (AE)

### Definition of Adverse Events

An adverse event is any untoward medical occurrence in a clinical trial subject who has signed informed consent form (ICF). It may be any unfavorable and unintended symptom, sign, laboratory abnormality, or disease, but it does not necessarily have to have a causal relationship with this treatment. In this trial, any adverse medical events occur from the time the subject signs the informed consent form to 30 days after treatment are collected and followed up until the adverse event is relieved or stable. Adverse events include the following:

1) Worsening of preexisting (before entering the clinical trial) medical conditions/diseases (including worsening of symptoms, signs and laboratory abnormalities);

2) Any new adverse medical conditions (including symptoms, signs and newly diagnosed diseases);

3) Clinically significant abnormal laboratory values or results that are not attributable to concurrent illness.

The investigator shall record in detail any adverse event occurred to the subject, including: name of adverse event, description of all related symptoms, occurrence time, severity, correlation with the investigational drug, duration, actions taken for the investigational drug, final result and outcome.

### Criteria for determining the severity of adverse events

Refer to NCI-CTCAE 5.0 grading criteria for adverse drug events. For adverse events not listed in the table of NCI-CTCAE 5.0, the following criteria can be referred to:

| **Level** | **Clinical Description of Severity** |
| --- | --- |
| 1 | Mild; asymptomatic or mild symptoms; clinical or diagnostic observations only; intervention not indicated. |
| 2 | Moderate; minimal, local or noninvasive intervention indicated; limiting age-appropriate instrumental activities of daily living (instrumental ADL, referring to preparing meals, shopping for groceries or clothes, using the telephone, managing money, etc.) |
| 3 | Severe or medically significant but not immediately life-threatening; hospitalization or prolongation of hospitalization indicated; disabling; limiting self care ADL. Self care ADL refer to: bathing, dressing and undressing, feeding self, using the toilet, taking medications, and not bedridden. |
| 4 | Life-threatening consequences; urgent intervention indicated |
| 5 | Results in death |

### Criteria for judging the relationship between adverse event and study drug

The collection of adverse events begins from the time of informed consent and continues through the end of the safety follow-up period. All adverse events must be collected and reported in the form of clinical report, regardless of the relationship between the event and investigational drug and whether the drug is used or not. Any discomfort or abnormal changes in objective laboratory test indicators complained of during treatment should be recorded truthfully, and the severity, duration, treatment measures and outcome of adverse events should be indicated. The investigator should comprehensively determine the relationship between adverse events and the investigational drug, such as whether the occurrence of adverse events has a reasonable time sequence with the medication, the characteristics of the investigational drug, the toxicological and pharmacological effects of the investigational drug, whether the subject uses other concomitant drugs, the subject's underlying diseases, medical history, family history, and challenge and rechallenge reactions. The possible association between adverse events and investigational drug is evaluated according to the five-level classification of "definitely related, possibly related, possibly unrelated, definitely unrelated and indeterminable".

## Serious Adverse Event (SAE)

### Definition of Serious Adverse Event

An SAE refers to any medical event that occurs during the course of a clinical trial and requires inpatient hospitalization or prolongation of existing hospitalization, causes disability/incapacity, jeopardizes work ability, is life-threatening or results in death, or causes congenital anomaly, etc. It includes the following medical events:

- Events resulting in death;
- Life-threatening event (defined as an event in which the subject is at immediate risk of death);
- Events requiring inpatient hospitalization or prolongation of existing hospitalization;
- Events which can result in permanent or significant disability/incapacity or jeopardize work ability;
- A congenital anomaly or birth defect;
- Other important medical events (defined as an event which jeopardizes the subject or requires intervention to prevent any of the conditions listed above).

### Hospitalisation

Adverse events that result in hospitalization (even if less than 24 hours) or prolonged hospitalization in a clinical study should be considered serious.

Hospitalization does not include the following:

• Rehabilitation facility

• Nursing home

• Routine emergency room admission (less than 24 hours)

• Day surgery (e.g. outpatients/day/ambulatory surgery)

• Social reasons (medical insurance reimbursement, etc.)

Hospitalization or prolongation of existing hospitalization that is unrelated to the deterioration of an AE is not an SAE itself. For example:

- Hospitalization due to underlying disease, without the occurrence of any new AE or aggravation of underlying disease (e.g.for the purpose of investigating lab abnormalities existing before the trial and up to now);
- Hospitalization for administrative reasons (e.g.routine annual physical examination);
- Hospitalization which occurs in the clinical trial period and is specified in the trial protocol (e.g.: procedure per trial protocol);
- Elective hospitalization unrelated to deterioration of AE (e.g.elective plastic surgery);
- Predetermined treatment or surgical procedure should be recorded in the entire trial protocol and/or subject’s personal baseline data.
- Hospitalization for blood product use only.

Diagnostic or therapeutic invasive (e.g., surgical), non-invasive operations should not be reported as adverse events, but should be reported when the disease condition that leads to the procedure meets the definition of an adverse event. If acute appendicitis develops during the adverse event reporting period, it should be reported as an adverse event, and the resulting appendectomy should be recorded as the treatment for that adverse event.

### Disease progression and death

Disease progression is defined as the deterioration of a subject's condition caused by the indication for which the investigational drug is administered, including radiographic progression and progression of clinical symptoms and signs. Both the occurrence of new metastasis relative to the primary neoplasm and the progression of original metastasis are considered progressive disease. Any event which is life-threatening, requires inpatient hospitalization or prolongation of existing hospitalization, or results in permanent or significant disability/incapacity or jeopardizes work ability, or is a congenital anomaly/birth defect, and is caused by the signs and symptoms of progressive disease should not be reported as an SAE in an expedited manner. Death caused by the signs and symptoms of progressive disease should be reported an SAE in an expedited manner. If there is any uncertainty as to whether an SAE is due to disease progression, it should be reported as an SAE.

Disease progression is expected in this study population and the word "disease progression" cannot be reported as an AE term. When disease progression occurs, the event used to confirm disease progression should be reported as an AE. For example, if a subject has a seizure that is determined to be related to brain metastasis, the AE term should be recorded as "seizure" and not "disease progression" or "brain metastasis".

"Death" is not defined as an AE or SAE term, but should be recorded as the outcome of the event; the event that caused or contributed to death should be recorded as the AE or SAE. If the cause of death is unknown and could not be determined at the time of reporting, the AE or SAE term is recorded as "death of unknown cause".

### Reporting system of serious adverse events

It starts from signing of the informed consent until 30 days (inclusive) after the last dose of study drug. During the trial, in case of any serious adverse event, no matter initial report or follow-up report, the investigator should immediately report it to the relevant unit according to the regulatory requirements within 24 hours after being informed of the SAE.

For serious adverse events, the symptoms, severity, correlation with the investigational drug, time-of-onset, handled time, action taken, follow-up time and method, and outcome should be recorded in detail. All serious adverse events should be followed up until they disappear, resolve to baseline or ≤ Grade 1, or reach a stable state, or are reasonably explained, or subjects are lost to follow-up, or die.

Below is supplemental information that is included in BMS-Sponsored trials. It is the responsibility of the Sponsor-Investigator to determine if this information regarding hospitalization is considered SAEs or not and whether it should be included in the protocol.

- - a visit to the emergency room or other hospital department < 24 hours, that does not result in admission (unless considered an important medical or life-threatening event)
  - elective surgery, planned prior to signing consent
  - admissions as per protocol for a planned medical/surgical procedure
  - routine health assessment requiring admission for baseline/trending of health status (eg, routine colonoscopy)
  - Medical/surgical admission other than to remedy ill health and planned prior to entry into the study. Appropriate documentation is required in these cases.
  - Admission encountered for another life circumstance that carries no bearing on health status and requires no medical/surgical intervention (eg, lack of housing, economic inadequacy, caregiver respite, family circumstances, administrative reason).
  - Admission for administration of anticancer therapy in the absence of any other SAEs (applies to oncology protocols)

**NOTE**: Although pregnancy and potential drug-induced liver injury (DILI), are not always serious by regulatory definition, these events must be reported within the SAEs timeline.

**NOTE**: Any component of a study endpoint that is considered related to study therapy should be reported as an SAE (eg, death is an endpoint, if death occurred due to anaphylaxis, anaphylaxis must be reported).

The **causal relationship** to study drug is determined by a physician and should be used to assess all adverse events (AE). The casual relationship can be one of the following:

**Related**: There is a reasonable causal relationship between study drug administration and the AE.

**Not related**: There is not a reasonable causal relationship between study drug administration and the AE.

The term "reasonable causal relationship" means there is evidence to suggest a causal relationship.

An appropriate SAE form (e.g. ex-US = CIOMS form or USA = Medwatch form) should be used to report SAEs to BMS. If the Sponsor-Investigator prefers to a study specific/Institutional form, it must be sent to the BMS ISR Trial Manager prior to study initiation for internal BMS review to ensure that at a minimum all of the data elements on the CIOMS form are present.

Please include the BMS Protocol number on the SAE form or on the cover sheet with the SAE form transmission.

- - The CIOMS form is available at: <https://cioms.ch/cioms-i-form/>
  - The MedWatch form is available at: https://www.fda.gov/safety/medical-product-safety-information/medwatch-forms-fda-safety-reporting

**NOTE**: If a CIOMS I or Medwatch form is selected for reporting, the investigator causal assessment must be provided in the narrative section, otherwise the BMS form must be used.**NOTE**: For studies with long-term follow-up period in which safety data are being reported, include the definition of end date of SAE collection.

SERIOUS ADVERSE EVENT COLLECTION AND REPORTING

All Serious Adverse Events (SAEs) that occur following the subject’s written consent to participate in the study through *100* days of discontinuation of dosing must be reported to BMS Worldwide Safety, whether related or not related to study drug**. If applicable,** SAEs must be collected that relate to any follow-up protocol-specified procedure (eg, a follow-up skin biopsy).

- Following the subject’s written consent to participate in the study, all SAEs, whether related or not related to study drug, are collected, including those thought to be associated with protocol-specified procedures. The Investigator should report any SAE occurring after these aforementioned time periods, which is believed to be related to study drug or protocol-specified procedure.
- An SAE report should be completed for any event where doubt exists regarding its seriousness;
- If the Investigator believes that an SAE is not related to study drug, but is potentially related to the conditions of the study (such as withdrawal of previous therapy or a complication of a study procedure), the relationship should be specified in the narrative section of the SAE Report Form.

SAEs, whether related or not related to study drug, and pregnancies must be reported to BMS within 24 hours \ 1 Business Day of becoming aware of the event. SAEs must be recorded on either CIOMS, MedWatch, or approved study specifi/institutional SAE form.

**SAE Email Address:**  Worldwide.Safety@BMS.com

**SAE Facsimile Number:** +1-609-818-3804

If only limited information is initially available, follow-up reports are required. (**Note**: Follow-up SAE reports should include the same investigator term(s) initially reported.)

If an ongoing SAE changes in its intensity or relationship to study drug or if new information becomes available, a follow-up SAE report should be sent within 24 hours \ 1 Business Day to BMS using the same procedure used for transmitting the initial SAE report.

All SAEs should be followed to resolution or stabilization.**NOTE:** The Sponsor-Investigator must provide a single centralized e-mail address (not an individual’s e-mail address) to be used by BMS to send AE/SAE report related queries. The single centralized e-mail address must be provided in the Study Contract and sent to aepbusinessprocess@bms.com.

**Expedited and periodic safety update reporting by BMS:**

It is the Sponsor-Investigator’s responsibility to report events to their Local HA. In addition, suspected serious adverse reactions (whether expected or unexpected) shall be reported by BMS to the relevant competent health authorities in all concerned countries according to local regulations (either as expedited and/or in aggregate reports).

In accordance with local regulations, BMS will notify Sponsor-Investigator of all reported SAEs that are suspected (related to the investigational product) and unexpected (ie, not previously described in the IB). An event meeting these criteria is termed a **Suspected, Unexpected Serious Adverse Reaction (SUSAR)**. Sponsor-Investigator notification of these events will be in the form of either a SUSAR Report or a Semi-Annual SUSAR Report. Sponsor-Investigator (or delegate) will receive these reports through the FastTrack portal.

Other important findings which may be reported by BMS as an Expedited Safety Report (ESR) include: increased frequency of a clinically significant expected SAE, an SAE considered associated with study procedures that could modify the conduct of the study, lack of efficacy that poses significant hazard to study subjects, clinically significant safety finding from a nonclinical (eg, animal) study, important safety recommendations from a study data monitoring committee, or Sponsor-Investigator or BMS decision to end or temporarily halt a clinical study for safety reasons.

Upon receiving an ESR from BMS, the Sponsor-Investigator **must review and retain the ESR with the IB**. Where required by local regulations or when there is a central IRB/IEC for the study, the Sponsor-Investigator will submit the ESR to the appropriate IRB/IEC. The investigator and IRB/IEC will determine if the informed consent requires revision. The investigator should also comply with the IRB/IEC procedures for reporting any other safety information.

NON-SERIOUS ADVERSE EVENT COLLECTION AND REPORTING

The collection of non-serious AE information should begin following the subject’s written consent to participate in the study. All non‑serious adverse events (not only those deemed to be treatment-related) should be collected continuously during the treatment period and for a minimum of 100 days following the last dose of study treatment.

Non-serious AEs should be followed to resolution or stabilization, or reported as SAEs if they become serious. Follow-up is also required for non-serious AEs that cause interruption or discontinuation of study drug and for those present at the end of study treatment as appropriate.

Non-serious Adverse Events (AE) are to be provided to BMS in aggregate via interim or final study reports as specified in the agreement or, if a regulatory requirement [eg, IND US trial] as part of an annual reporting requirement.

PREGNANCY

If, following initiation of the investigational product, it is subsequently discovered that a study participant is pregnant or may have been pregnant at the time of investigational product exposure, including during at least 5 half-lives after product administration, the investigational product will be permanently discontinued in an appropriate manner (eg, dose tapering if necessary for participant).

The Sponsor-Investigator must immediately notify [**Worldwide.Safety@bms.com**](mailto:Worldwide.Safety@bms.com) of this event and complete one of the following forms **within 24 hours of awareness** of the event via either the CIOMS, MedWatch or appropriate Pregnancy Surveillance Form in accordance with **SAE reporting procedures.**

Protocol-required procedures for study discontinuation and follow-up must be performed on the participant.

Follow-up information regarding the course of the pregnancy, including perinatal and neonatal outcome and, where applicable, offspring information must be reported on the **CIOMS, MedWatch, BMS Pregnancy Surveillance Form, or approved site SAE form**. A BMS Pregnancy Surveillance Form may be provided upon request.

Any pregnancy that occurs in a female partner of a male study participant should be reported to BMS. Information on this pregnancy will be collected on the Pregnancy Surveillance Form. In order for Sponsor-Investigator or designee to collect any pregnancy surveillance information from the female partner, the female partner must sign an informed consent form for disclosure of this information.

LABORATORY TEST ABNORMALITIES

All laboratory test results captured as part of the study should be recorded following institutional procedures. Test results that constitute SAEs should be documented and reported to BMS as such.

The following laboratory abnormalities should be documented and reported appropriately:

- any laboratory test result that is clinically significant or meets the definition of an SAE
- any laboratory abnormality that required the participant to have study drug discontinued or interrupted
- any laboratory abnormality that required the subject to receive specific corrective therapy.

It is expected that wherever possible, the clinical rather than laboratory term would be used by the reporting investigator (eg, anemia versus low hemoglobin value).

OTHER SAFETY CONSIDERATIONS

Any significant worsening noted during interim or final physical examinations, electrocardiograms, X-rays, and any other potential safety assessments, whether or not these procedures are required by the protocol, should also be recorded as a non-serious or serious AE, as appropriate, and reported accordingly.

ADVERSE EVENT REPORTING FOR SPECIFI SITUATIONS (remove if not applicable):

- **Immune-Mediated AEs (Product Specific, Usually a regulatory requirement)**

Immune-Mediated AEs are required in **ISR Protocols with Registrational Intent**.

Every AE must be assessed by the investigator with regard to whether it is considered immune-mediated. For events which are potentially immune-mediated, additional information must be collected.

Immune-mediated adverse events (IMAEs) are AEs consistent with an immune-mediated mechanism or immune-mediated component for which non-inflammatory etiologies (eg, infection or tumor progression) have been ruled out. IMAEs can include events with an alternate etiology which were exacerbated by the induction of autoimmunity. Information supporting the assessment will be collected.

- **AEs of Special Interest (Product Specific. Usually a regulatory requirement)**

ADVERSE EVENT RECONCILIATION PROCESS

The Sponsor-Investigator (or designee) will reconcile the clinical database AE cases (case level only) transmitted to BMS Global Pharmacovigilance ([Worldwide.Safety@bms.com](mailto:Worldwide.Safety@bms.com)).

- The Sponsor-Investigator will request the SAE reconciliation report (and include the BMS protocol number) from BMS GPV&E ([aepbusinessprocess@bms.com](mailto:aepbusinessprocess@bms.com)) every 3 months and prior to data base lock or final data summary
- GPV&E will send the Sponsor-Investigator the report to verify and confirm all AE and SAEs have been transmitted to BMS GPV&E.
- The data elements listed on the GPV&E reconciliation report will be used for case identification purposes. If the Sponsor-Investigator determines a case was not transmitted to BMS GPV&E, the case should be sent immediately to BMS ([Worldwide.Safety@bms.com](mailto:Worldwide.Safety@bms.com)).

PRODUCT QUALITY COMPLAINTS (PQCs)

**Definition**

Any communication about a **BMS Product** that alleges deficiencies related to identity, quality, durability, reliability, safety, effectiveness, performance, tampering, diversion, and/or counterfeiting/falsification of a drug, combination product, or device after it is released for distribution to market or clinic by either: (1) BMS or (2) distributors or partners for whom BMS manufactures the material. This includes all components co-packaged with the drug, such as drug containers, delivery system, labelling, and inserts.

**BMS product:** Commercial or investigational materials (i.e., drugs, devices, biologics or any combination thereof) and their packaging components, whether they are produced or distributed by BMS or by third parties under contract with BMS, and products that are being manufactured for BMS by third parties.

**Reporting**

Product Quality Complaints must be reported to BMS **within one (1) business day** of awareness to [**IMPQualityComplaints@bms.com**](mailto:IMPQualityComplaints@bms.com)**.**

In the event of a suspected product quality issue, the affected product must be quarantined immediately at the Investigational site.

The affected product should not be disposed unless retention presents a risk to personnel (e.g., cytotoxic, risk of injury from broken glass or sharps).

When reporting, as much product information as possible should be reported. At a minimum, but not limited to, include:

- ISR Study number, site reference, product description, impacted batch number, container number(s), photographs, and any other supporting information

# Study Management

## Ethical guidelines and informed consent form

This clinical trial must comply with the Declaration of Helsinki (1996 Edition), Good Clinical Practice (GCP) promulgated by the National Medical Products Administration (NMPA), and relevant regulations. The trial must be approved by the ethics committee prior to the start; Any amendment to the protocol during the trial should be reported to the Ethics Committee and approved.

The clinical investigators will follow all applicable rules and regulations to protect subjects. The informed consent form used during the informed consent process must be approved by the ethics review board and available for inspection.

The clinical investigator must inform the subject that participation in the clinical trial is voluntary, that the subject has the right to withdraw from the trial at any stage of the trial at any time without discrimination or retaliation, and that his/her medical treatment and rights and interests are not affected, and that he/she can continue to receive other forms of treatment. The subjects must be informed that their personal data in the trial will be kept confidential. The subjects shall also be informed of the nature of clinical trial, trial objective, expected possible benefits and possible risks and inconveniences, other alternative treatment options and the rights and obligations of the subjects conforming to the provisions of Declaration of Helsinki, so as to give the subjects sufficient time to consider whether they are willing to participate in the trial and sign the informed consent form.

Prior to performing any protocol-required procedures, the subjects must:

- Be informed of the study as well as all contents and terms of the informed consent form.
- Be given adequate time to ask questions and consider participation.
- Voluntary consent to participate in the study.
- Sign and date the IRB/IEC-approved informed consent form.

Any significant changes in the study require a protocol amendment. The investigator should not make any changes to the study without prior IRB/IEC approval unless it is necessary to eliminate an obvious and immediate hazard to the subject. Changes to the protocol to eliminate an apparent immediate hazard to subjects may be implemented immediately, but must be documented in the protocol amendment, reported to the IRB/IEC, and submitted to the appropriate regulatory agencies within the required timeline. All protocol modifications must be subject to the same review and approval process as the original protocol.

The investigator is responsible for submitting the interim report/annual review report regularly according to the relevant requirements of the Ethics Committee, and notifying the Ethics Committee that the trial has ended after the trial is completed

## Protocol amendment

This "Clinical Study Protocol" and "Clinical Study Case Report Form" are developed by the principal investigator and will be implemented after approval by the Ethics Committee of this hospital. During the clinical trial, any amendment to the trial protocol should be consulted by the investigator and approved by the Ethics Committee.

## Quality assurance of clinical trial

In order to ensure the quality of clinical trials, the principal investigator will jointly discuss and develop the clinical study plan before the formal trial is initiated. Protocol training should be performed for all relevant study personnel participating in the clinical trial.

Investigational medicinal products must be managed according to SOPs, including drug supply, storage, dispensing and recovery, and destruction.

According to the GCP guidelines, necessary procedures should be adopted during study design and conduct to ensure the accuracy, consistency, completeness, and authenticity of the data collected. All results and abnormal findings observed in the clinical trial should be timely and seriously verified and recorded to ensure the data integrity. All instruments, equipment, reagents, and standard substances used for various examination items in the clinical trial should have strict specifications and must work in a normal state.

# Statistical analysis of data

## Sample Size

- The primary endpoint is pCR. This study has 80% power to detect an increase in pCR rate from 5% to 20% with a one-sided alpha of 0.05. Considering a 10% drop-out rate, 46 patients will be enrolled.

## Analysis Population

- Intent to treat (ITT): It is defined as all subjects who follow the ITT principle.
- Per-Protocol Set (PPS): PPS is a subset of ITT. It is defined as the subjects who have no major protocol deviations or protocol deviations that have major impact on the study results during the study.
- Safety Set (SS): It refers to the subjects who are enrolled and receive at least one dose of the investigational drug and constitute the SS for this study.
- The efficacy evaluation in this study will be based on ITT and PPS, where ITT is the primary analysis set. Safety analysis will be based on SS.

## Statistical Methods

This study is a single-arm design. Unless otherwise specified, all data will be summarized by treatment group and appropriate statistics will be used according to data type: measurement data are mean, standard deviation STD, median, minimum and maximum; count data are frequency and proportion for descriptive statistics; time-event data will be estimated by Kaplan-Meier (KM) product limit method for survival time; if necessary, survival curve will be plotted and 95% confidence interval of survival time will be estimated.

# Protection of subjects

Subject information will be kept strictly confidential and will not reveal the patient's personal information unless required by applicable law. If necessary, the government administration department, hospital ethics committee and relevant personnel may consult the patient data as needed.

# Clinical study progress

The estimated enrollment date of the first subject: January 2023

Enrollment duration: 1 year

Follow-up after treatment: 5 years
